# Synthesis of hydroxytyrosol analogs with enhanced antioxidant and cytostatic properties against MG‐63 human osteoblast‐like cells and their potential implications for bone health

**DOI:** 10.1002/ardp.202400469

**Published:** 2024-11-16

**Authors:** Eleftheria A. Georgiou, Ioanna Kalpaktsi, Katerina Gioti, Maria Choleva, Elizabeth Fragopoulou, Alexios‐Leandros Skaltsounis, Roxane Tenta, Ioannis K. Kostakis

**Affiliations:** ^1^ Division of Pharmaceutical Chemistry, Department of Pharmacy National and Kapodistrian University of Athens Athens Greece; ^2^ Department of Nutrition & Dietetics, School of Health Sciences and Education Harokopio University Athens Greece; ^3^ Division of Pharmacognosy and Natural Products Chemistry, Department of Pharmacy National and Kapodistrian University of Athens Athens Greece

**Keywords:** antioxidant, hydroxytyrosol (HT), osteoblasts, polyphenols

## Abstract

Sixteen novel hydroxytyrosol (HT) analogs with substitutions at the C‐1 position of the HT aliphatic side chain were synthesized and evaluated for their cytostatic activity against MG‐63 human osteoblast‐like cells and for their antioxidant properties. The results revealed that these analogs exhibited significantly higher inhibitory activity compared with HT, which served as the positive control. Among these, the cyclo‐substituted compounds stood out as particularly potent, demonstrating strong radical scavenging abilities and notable cytostatic effects against MG‐63 cells. These findings suggest that the cyclo‐substituted HT analogs hold considerable promise for the development of novel antioxidants with potential applications in bone physiology.

## Graphical abstract

New hydroxytyrosol derivatives, substituted at the C‐1 position of the side chain, were synthesized using a multistep reaction procedure. These cyclo‐substituted compounds demonstrated significantly higher antioxidant activity, showcasing strong radical scavenging abilities. They exhibited notable cytostatic effects against MG‐63 human osteoblast‐like cells.

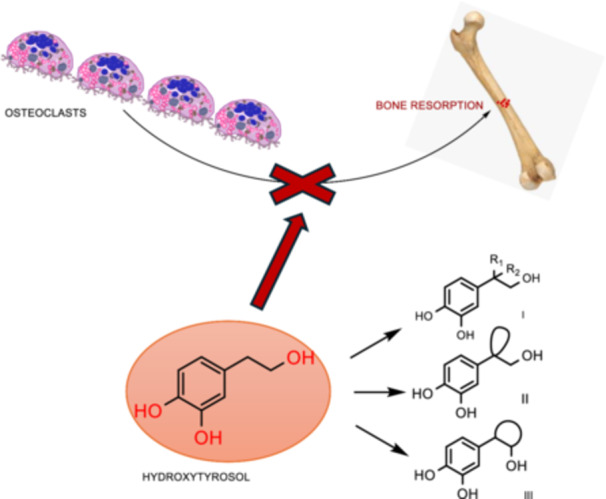

## 1 INTRODUCTION

**Figure 1:**
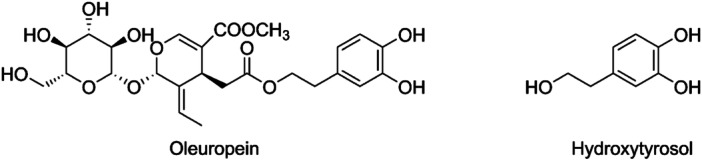
Structure of Oleuropein and hydroxytyrosol.

Polyphenols constitute a diverse family of compounds found abundantly in plant‐based foods such as cranberries, grapes/wine, olives, and walnuts, each showcasing various biological activities. Polyphenols reportedly exert physiological effects against diseases such as cancer, arteriosclerosis, hyperlipidemia, and osteoporosis.^[^ ^1^, ^2^ ^]^ Within the polyphenol family, hydroxytyrosol (HT) stands out as one of the most significant components, predominantly sourced from olive leaves and pulp. HT is a metabolite of oleuropein (OE), the major glycoside present in olive products (Figure 1).

HT exhibits a wide array of biological properties owing to its potent antioxidant activity.^[^ ^3^, ^4^, ^5^, ^6^, ^7^ ^]^ Free radicals, which are formed as a result of metabolic processes in the body, can inflict cellular damage, leading to premature aging. HT's remarkable capacity for scavenging free radicals allows it to mitigate this process, known as oxidative stress, which has emerged as a critical lifestyle risk factor associated with the loss of bone mineral density.^[^ ^8^, ^9^, ^10^, ^11^, ^12^ ^]^ Bone tissue is continuously remodeled through the concerted actions of bone cells, which include bone resorption by osteoclasts and bone formation by osteoblasts, whereas osteocytes (differentiated osteoblasts embedded inside mature bone tissue) act as mechanosensors of the bone remodeling process. This process is under the control of local and systemic factors, contributing to bone homeostasis. An imbalance between bone resorption and bone formation can result in bone diseases, including osteoporosis. The antioxidant activity that has been reported to have a positive impact on bone remodeling is particularly vital in the context of osteoporosis development. Various studies indicate that targeting the production of reactive oxygen species (ROS) may be a sufficient approach for future therapies related to bone diseases.^[^ ^13^, ^14^, ^15^ ^]^

Moreover, several studies have highlighted HT's ability to enhance calcium deposition and hinder the formation of osteoclasts in a dose‐dependent manner.^[^ ^2^, ^16^, ^17^, ^18^, ^19^ ^]^ By reducing osteoclast activity, HT can mitigate bone weakening and reabsorption, ultimately leading to a potential reduction in age‐related bone loss.^[^ ^20^, ^21^ ^]^ Therefore, HT holds promise as a beneficial compound for individuals suffering from age‐related bone loss and osteoporosis.^[^ ^17^, ^22^ ^]^

Furthermore, this research area could have wide applications such as in the field of human health in space; it is known that in the microgravity environment, this equilibrium is lost due to reduced loading stimuli. Bone resorption prevails on bone formation, leading to bone mass loss at a rate of about 10 times that of osteoporosis occurrence on Earth. Microgravity‐induced osteopenia is, therefore, a significant and unresolved health risk for space travelers, which may lead to irreversible changes that weaken skeletal integrity and increase the onset of fracture injuries.^[^ ^23^, ^24^ ^]^

**Figure 2:**
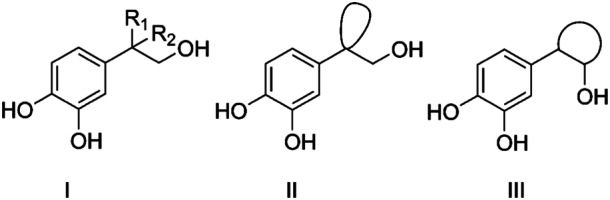
Structure of hydroxytyrosol (HT) analogs.

Based on the available data, we synthesized HT analogs to investigate their potential antioxidant activity and their biological effects on human osteoblasts. The new compounds are HT derivatives substituted on the aliphatic side chain, categorized into three distinct series (Figure 2). Specifically, compounds of general formulas I and II are substituted on the α‐carbon of the side chain with an alkyl and a cycloalkyl group, respectively; compounds of general formula III have a cycloalkyl group on both carbons of the side chain. Given that most HT analogs are lipophilic esters of the aliphatic hydroxyl group, we focused on modifying the α‐carbon of the aliphatic side chain, aiming to increase the sp3 fraction. With this work, we intend to study the influence of these structural modifications on both the antioxidant properties and biological activity in human osteoblasts.

## 2 RESULTS AND DISCUSSION

### 2.1 Chemistry

**Scheme 1:**
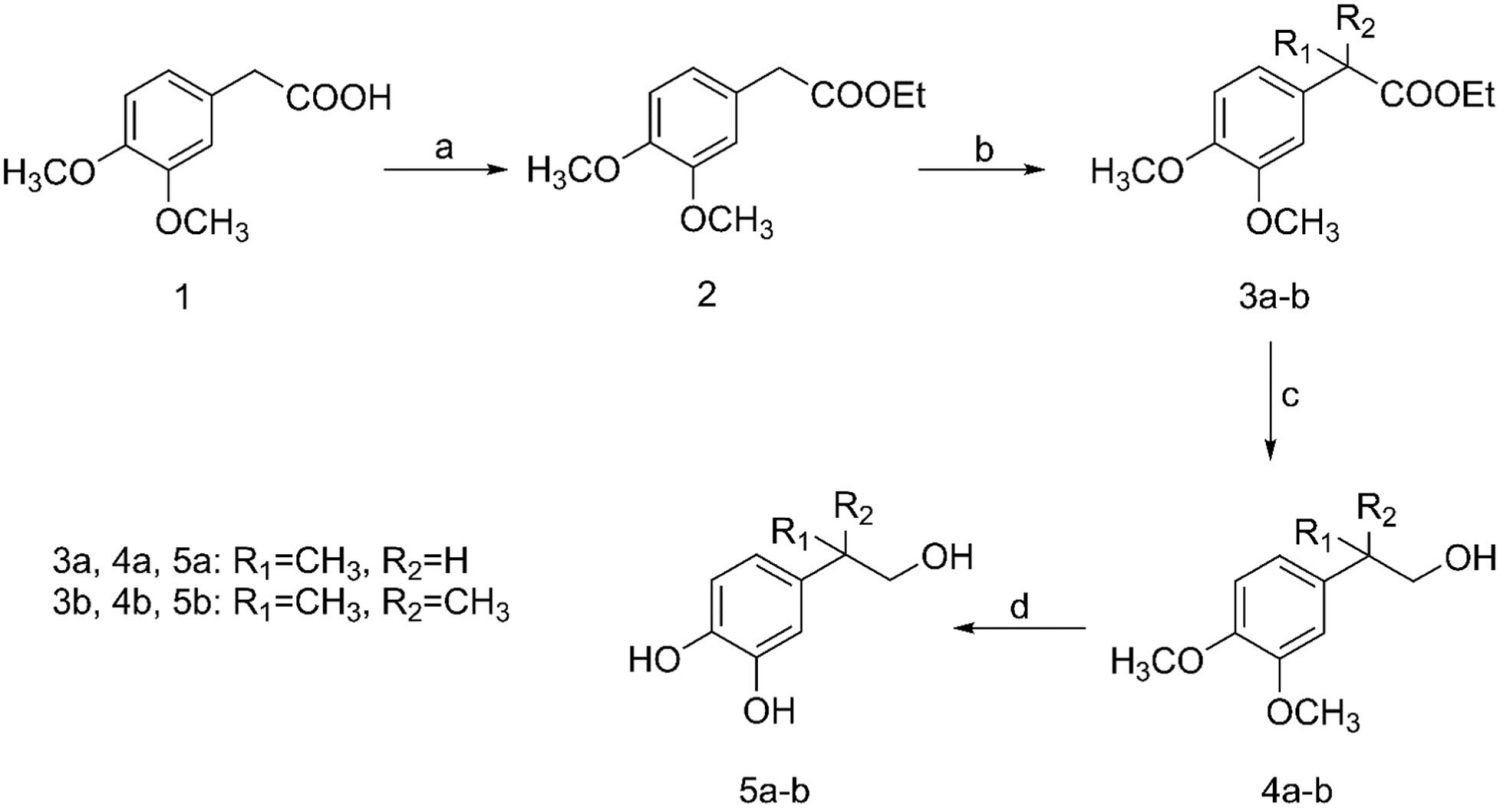
Reagents and conditions: (a) ethanol abs, c. H_2_SO_4_, reflux; (b) NaH, CH_3_I, dimethylformmide (DMF) dry; (c) LiAlH_4_, tetrahydrofurane (THF) dry; (d) pyridine hydrochloride, 180°C.

The desired compounds **5a–b** were prepared using 3,4‐dimethoxyphenylacetic acid (**1**) as the starting material, which upon acid‐catalyzed esterification with ethanol afforded ester **2**. Subsequent treatment with methyl iodide under basic conditions provided the desired mono and disubstituted esters **3a–b**, which upon reduction by LiAlH_4_ afforded the corresponding alcohols **4a–b** (Scheme 1). Finally, deprotection of the later compounds using pyridine hydrochloride furnished the HT derivatives **5a–b**.

**Scheme 2:**
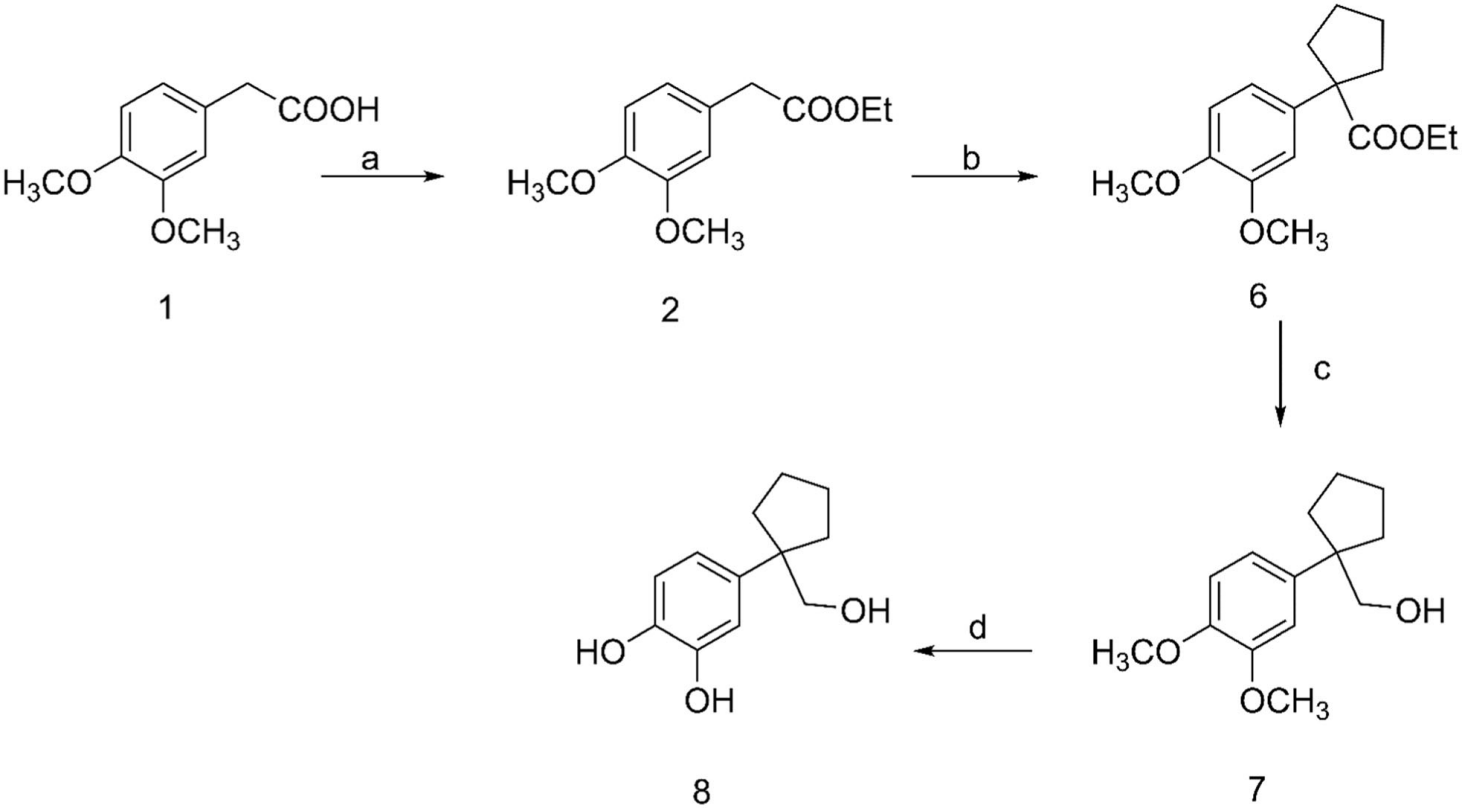
Reagents and conditions: (a) ethanol abs, c. H_2_SO_4_, reflux; (b) NaH, Br(CH_2_)_5_Br, DMF dry; (c) LiAlH_4_, THF dry; (d) ΒBr_3_, CH_2_Cl_2_ dry, −40°C.

To prepare the cyclopentano‐substituted compound **8**, ethyl ester **2** was reacted with 1,5‐dibromopentane to provide ester **6**, which upon reduction with LiAlH_4_ yielded the corresponding alcohol **7** (Scheme 2). Deprotection of compound **7** was achieved by treatment with BBr_3_ at −40°C, resulting in the desired compound **8**.

**Scheme 3:**
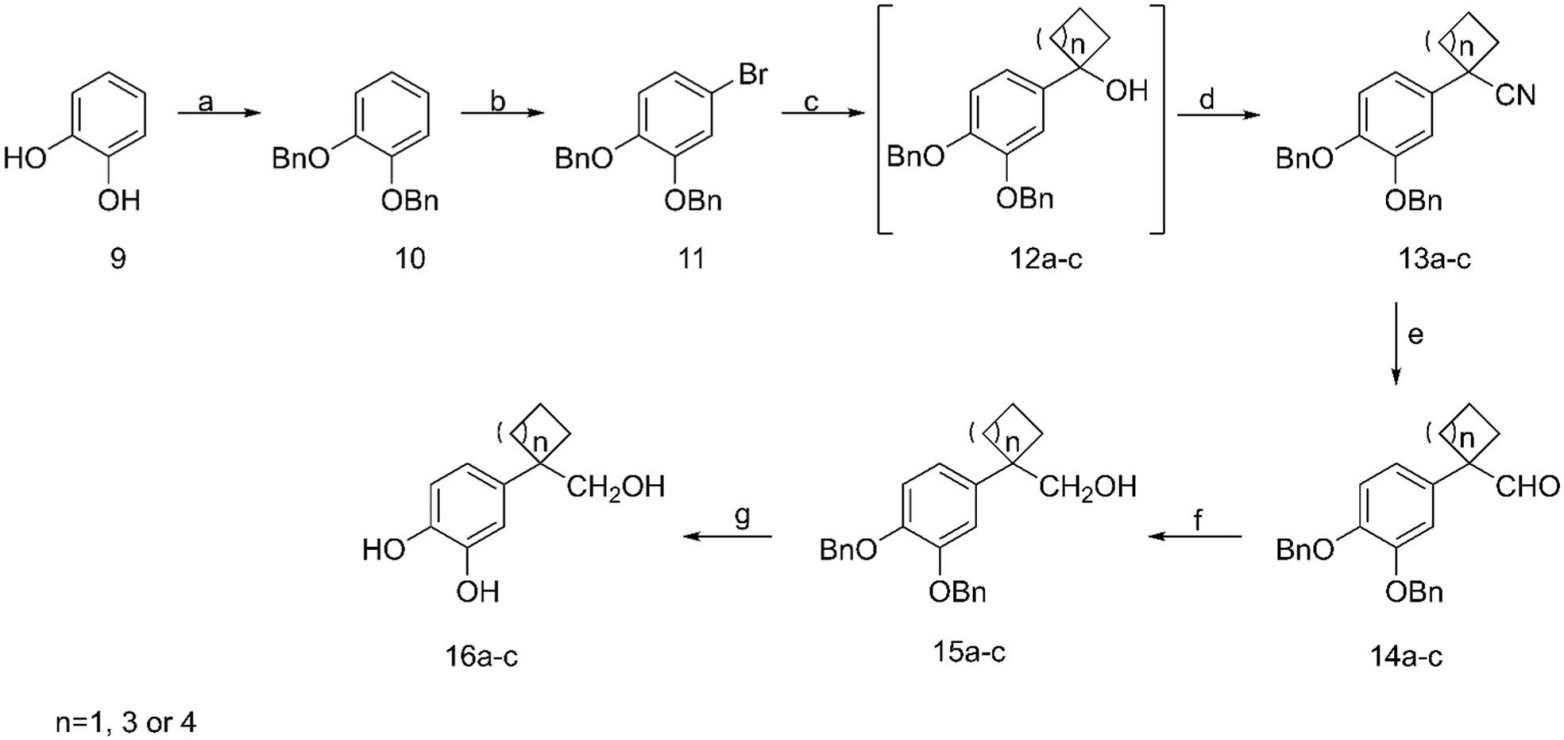
Reagents and conditions: (a) K_2_CO_3_, BnBr, acetone, reflux, 4 h; (b) N‐bromosuccinimide (NBS), acetonitrile (ACN), rt, 2.5 h; (c) *n*‐BuLi, proper ketone, THF dry, −78°C –0°C, 2 h; (d) trimethylsilyl cyanide (TMSCN), InCl_3_, CH_2_Cl_2_ dry, 0°C – rt, 2 h; (e) DIBAL (1 M in DCM), CH_2_Cl_2_ dry, −70°C, 1 h; (f) NaBH_4_, abs. MeOH, 0°C–rt, 2 h; (g) (k) BBr_3_, dry CH_2_Cl_2_, −40°C, 4 h or H_2_, Pd/C, 50 psi, abs. EtOH, rt, 4 h.

Our attempts to apply a similar methodology for the preparation of the corresponding cyclobutano, cyclohexyl, or cycloheptyl analogs were not successful. This could be due to the instability of these compounds under the harsh conditions of the final step of deprotection. Consequently, we developed a new methodology for the preparation of the desired derivatives. We used catechol **9**, which was treated with benzyl bromide to yield the dibenzyloxy derivative **10** (Scheme 3). Treatment of compound **10** with N‐bromosuccinimide in acetonitrile resulted in the desired bromide **11**. The reaction of **11** with n‐BuLi at −78°C, followed by the appropriate ketone, provided the desired cycloalcohols **12a–c**. Since these derivatives were highly unstable due to their dehydration to the corresponding olefins, they were not isolated or identified, but converted to the corresponding nitriles **13a–c** by treatment with trimethylsilyl cyanide and InCl. Reduction of the nitriles **13a–c**, with diisobutylaluminium hydride (DIBAL), at −70°C, resulted in the corresponding aldehydes **14a–c**. Then, after the reduction of aldehydes **14a–c** with sodium borohydride, the corresponding alcohols **15a–c** obtained excellent yield (more than 80%), which upon catalytic hydrogenation yielded the desired compounds **16a–c** (Scheme 3).

**Scheme 4:**
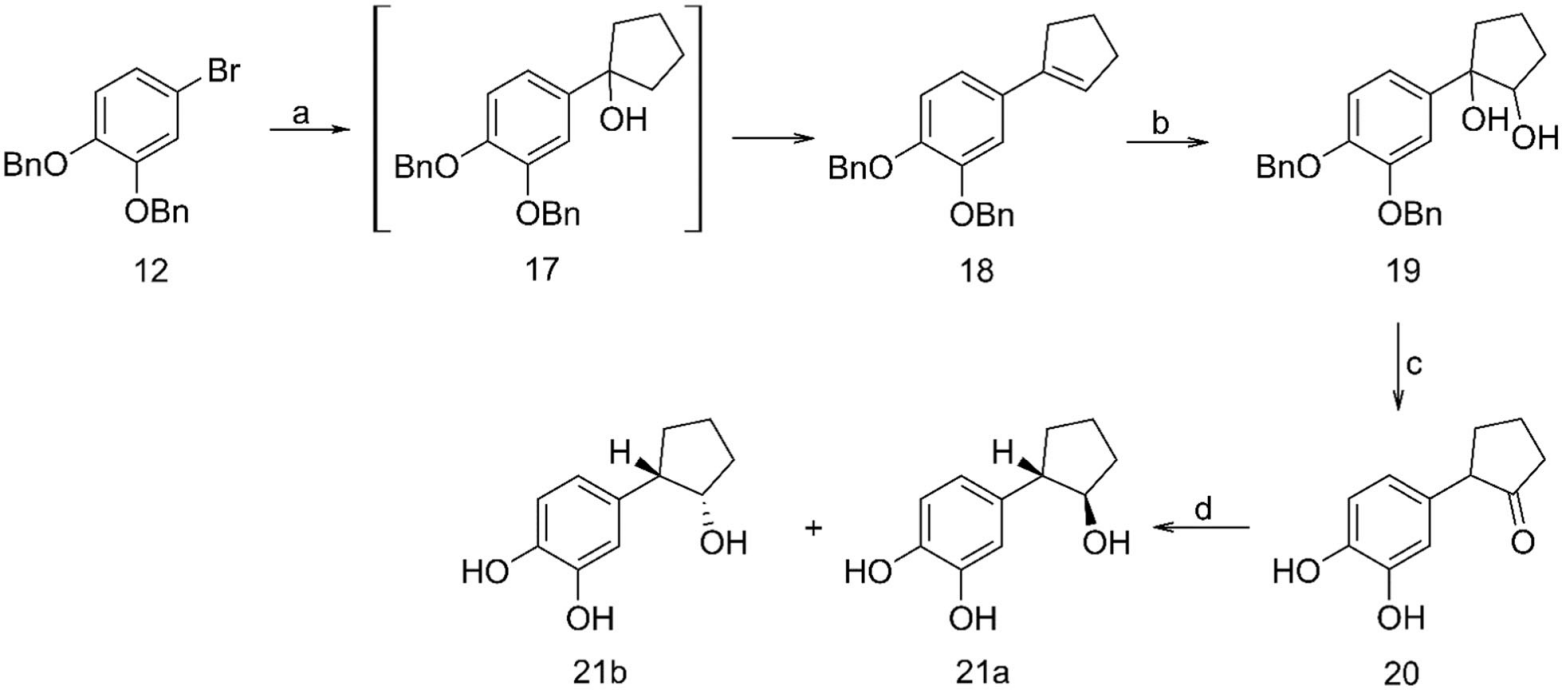
Reagents and conditions: (a) n‐BuLi, cyclopentanone, THF dry −78°C; (b) OsO_4_, NMO, t‐BuOH, acetone, H_2_O; (c) H_2_, Pd/C, 50 psi, EtOH abs.; (d) NaBH_4_, MeOH dry.

For the synthesis of the cyclopentyl derivative **21**, the intermediate compound **17** was spontaneously dehydrated to afford olefin **18**, which upon reaction with OsO_4_, in the presence of N‐methyl‐morpholine N‐oxide, yielded diol **19**. Compound **19** was then easily deprotected by hydrogenation over palladium on activated carbon to provide, in one step, ketone **20** (Scheme 4). This compound likely resulted from the spontaneous dehydration of the intermediate deprotected diol. The desired alcohols **21a–b** were obtained via reduction of ketone **20** with sodium borohydride in anhydrous methanol (Scheme 4).

**Scheme 5:**
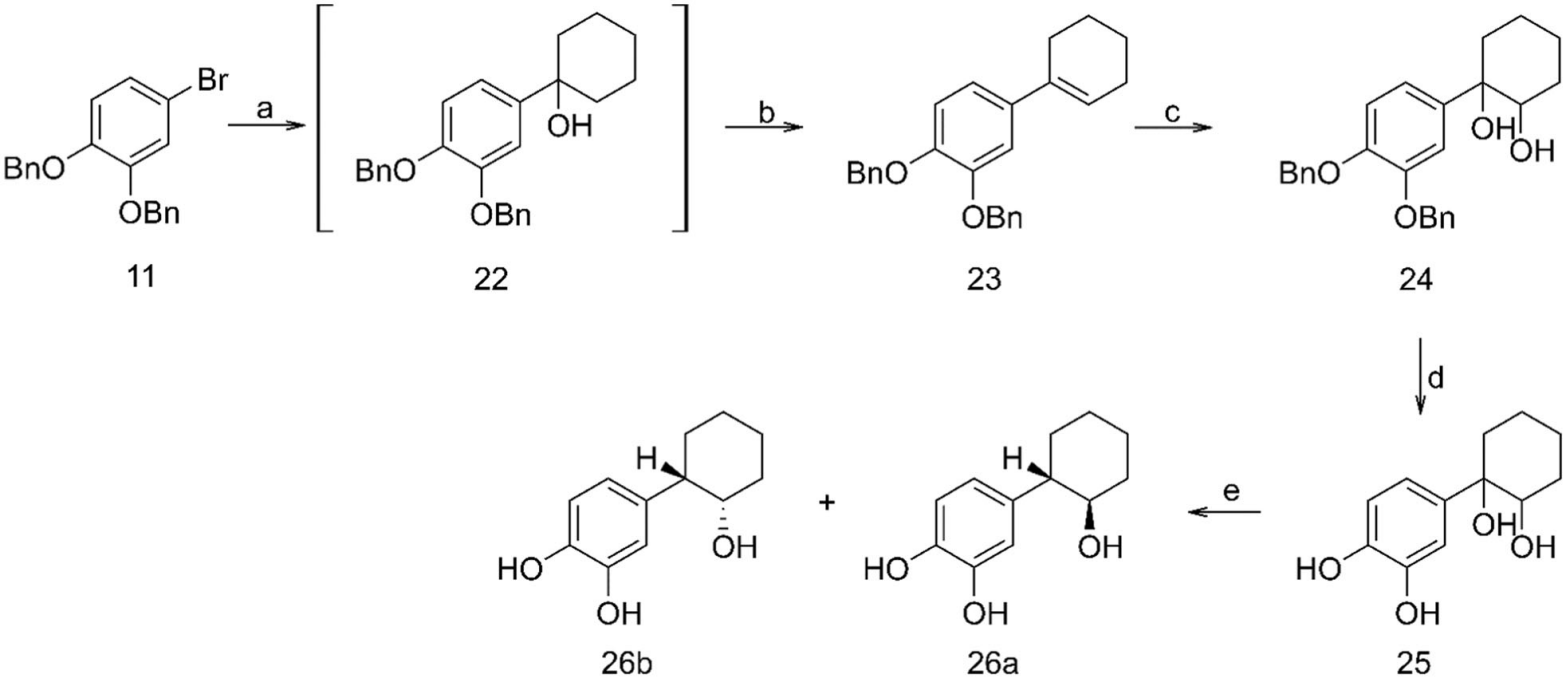
Reagents and conditions: (a) n‐BuLi, cyclohexanone, dry THF, −78°C; (b) p‐TsOH, THF; (c) OsO_4_, NMO, t‐BuOH, acetone, H_2_O; (d) H_2_, Pd/C, 50 psi, EtOH abs.; (e) CF_3_COOH, Et_3_SiH.

Regarding the synthesis of the corresponding cyclohexyl derivative **26**, an analogous procedure was followed, with minor modifications, due to the different stability of the intermediates. Thus, diol **24** was easily deprotected by hydrogenation to provide compound **25**, which upon reductive dihydroxylation, in the presence of triethylsilane in trifluoroacetic acid, yielded the desired alcohol **26** (Scheme 5).

**Scheme 6:**
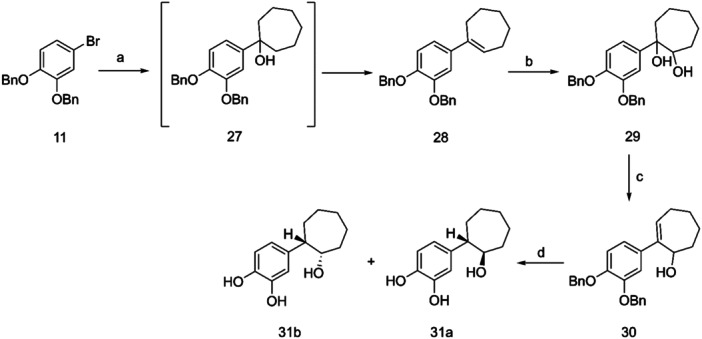
Reagents and conditions: (a) n‐BuLi, cycloeptanone, THF dry, −78°C; (b) OsO_4_, NMO, t‐BuOH, acetone, H_2_O; (c) p‐TsOH, THF; (d) H_2_, Pd/C, 50 psi, EtOH abs.

Regarding the synthesis of the cycloheptyl derivative **31** (Scheme 6), the intermediate compound **29** proved unstable; thus, we have developed a slightly different procedure, using olefin **28** as the starting material. *Syn*‐hydroxylation of compound **28**, upon reaction with OsO_4_, in the presence of *N*‐methyl‐morpholine *N*‐oxide, yielded diol **29**, which upon dehydration in the presence of *p*‐toluenesulfonic acid afforded alcohol **30**. Compound **30** was then easily reduced by hydrogenation over palladium on activated carbon to the desired compound **31**.

**Scheme 7:**
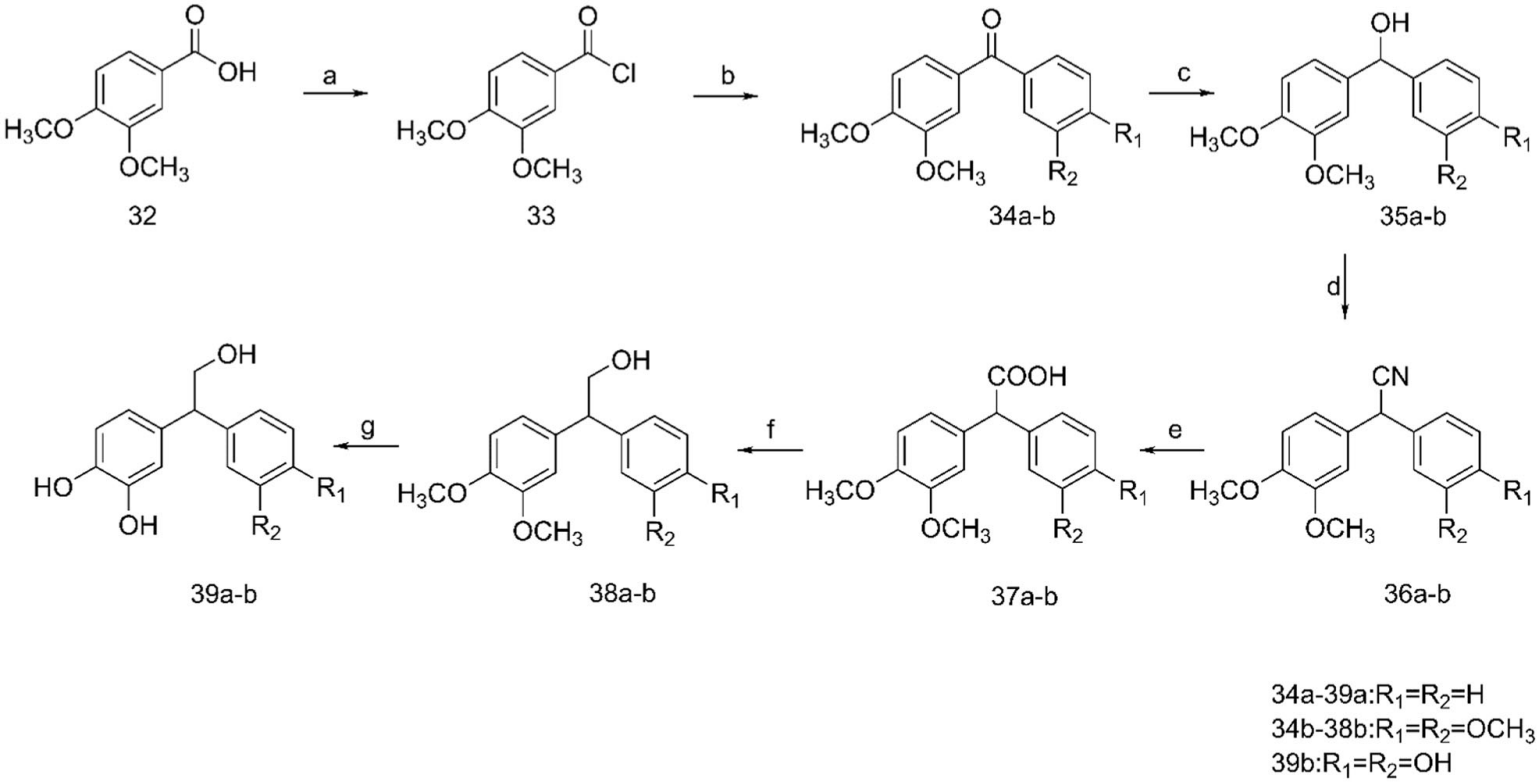
(a) SOCl_2_, rt; (b) AlCl_3_, benzene/dimethoxycatechol, ClCH_2_CH_2_Cl, rt; (c) ΝaΒΗ_4_, MeOH dry, 0°C to rt; (d) TMSCN, BF_3_. Et_2_Ο, CH_2_Cl_2_, rt; (e) ΝaΟΗ, EtOH, reflux; (f) LiAlH_4_, THF dry, rt; (g) pyrimidine hydrochloride, 170°C or BBr_3_, CH_2_Cl_2_, 0°C to rt.

Finally, for the preparation of derivatives **39a–b**, chloride **33** reacted with benzene or dimethoxycatechol, via a classic Friedel–Crafts reaction, in the presence of AlCl_3_, providing benzophenones **34a** and **34b**, respectively (Scheme 7). Compounds **34a–b** were then reduced using NaBH_4_, and the resulting alcohols **35a–b** were converted to the corresponding nitriles **36a–b** using trimethylsilyl cyanide and BF_3_.Et_2_O. Nitriles **36a–b** were then easily hydrolyzed by aqueous NaOH solution, resulting in carboxylic acids **37a–b**, which were further converted to carbinols **38a–b** upon reduction with LiAlH_4._ The target compound **39a** was prepared by treatment of **38a** with pyridine hydrochloride, while in the case of the catechol‐substituted compound **39b**, BBr_3_ was utilized.

### 2.2 Pharmacology/Biology

#### 2.2.1 Effect on cell proliferation

The biological activity of the new compounds was evaluated in vitro against human MG‐63 cells (osteosarcoma cell line with osteoblastic phenotype). The results of the MTT dye reduction assay, expressed as 50% inhibitory concentrations (IC_50_) in µM, are depicted in Table 1. Most of the new compounds show interesting activities, with IC_50_ values varying within the range of 4.3–30 µM with two of them being more potent than HT. In general, 1,1‐cyclobutano‐, cyclopentano‐, cyclohexano‐, and cycloheptano compounds **8**, **16a**, **16b**, and **16c**, respectively, appear considerably more active when compared with HT and the 1,2‐substituted analogs **21**, **26**, and **31**. Interestingly, in all cases, the cyclohexano‐substituted compound **16b** proved to be more potent. These data indicate that the a‐substitution on the HT side chain with a cyclo group affords higher activities against osteosarcoma cells. On the contrary, the monomethyl and dimethyl analogs **5a** and **5b**, respectively, proved to be less active than the 1,1‐cyclosubstituted compounds; nevertheless, their activity is comparable with HT.

**Table 1:** Accumulative results of the activities on cell proliferation of all compounds tested.

A/A	Compound	IC_50_ (μΜ)	A/A	Compound	IC_50_ (μΜ^a^)
1	**5a**	18.4	9	**25**	>40
2	**5b**	15	10	**26a**	10.8 ± 1.1
3	**8**	8	11	**26b**	30 ± 2.8
4	**16a**	10.5 ± 1.8	12	**31a**	28.3 ± 2.3
5	**16b**	4.3 ± 0.4	13	**31b**	24.8 ± 0.2
6	**16c**	7.8 ± 0.5	14	**39a**	25.4 ± 1
7	**21a**	30.1 ± 1.2	15	**39b**	6.4 ± 0.3
8	**21b**	20.9 ± 1.5	16	Hydroxytyrosol	14.7 μΜ

^a^ Each experiment was performed in triplicate and mean values ± SD are reported.

Notably, diol **25**, appears to be considerably less active when compared with the corresponding 1,1 and 1,2 substituted compounds. Regarding the diphenyl analogs **39a** and **39b**, the bis‐HT analog **39b** proved to be the most effective against MG‐63 cells when compared with the phenyl‐substituted compound **39a**. These results strongly suggest the importance of the substitution on HT's side chain, thus providing evidence regarding the favorable scaffold and substitution for the maximization of activity of these classes of compounds.

#### 2.2.2 Antioxidant evaluation

To evaluate the antioxidant capacity of the molecules, two assays were performed. The first assay examines the capacity of the molecules to scavenge free radicals (2,2‐diphenyl‐1‐picryhydrazyl [DPPH] assay). Initially, different concentrations (1–75 μΜ) of HT were tested and its EC_50_ was calculated at 55.0 ± 8.6. Afterward, the HT derivatives were tested and the % inhibition in each concentration in the range of 5–35 along with their EC_50_ values are presented in Table 2. In the low concentration of 5 μM, only the molecules **16b**, **16c**, **25**, **26a**, and **26b** were revealed to be more potent scavengers than the initial compound, while in the higher concentration, almost all the molecules revealed higher than HT scavenging capacity. This is also observed in EC_50_ values where all derivatives except compound **26b** display significantly lower values than the initial compound.

**Table 2:** Capacity of compounds to scavenge DPPH radical.

	% Scavenging of DPPH radical^a^					
A/A	Compound	5 μΜ	10 μΜ	20 μΜ	35 μΜ	EC_50_ (μΜ)^b^
1	**5a**	NT	NT	NT	NT	NT
2	**5b**	NT	NT	NT	NT	NT
3	**8**	16.2 ± 5.2	20.1 ± 4.5	30.2 ± 5.9	51.5 ± 5.8	32.9 ± 4.8
4	**16a**	17.3 ± 5.4	22.4 ± 5.8	30.1 ± 5.1	52.4 ± 16.4	32.7 ± 8.6
5	**16b**	28.1 ± 9.6	26.5 ± 5.2	35.7 ± 5.4	57.4 ± 11.9	28.7 ± 7.7
6	**16c**	20.2 ± 3.9	24.4 ± 4.1	36.3 ± 6.9	56.8 ± 10.2	29.3 ± 5.3
7	**21a**	19.1 ± 5.8	23.1 ± 4.5	29.1 ± 6.3	49.7 ± 13.0	34.3 ± 8.2
8	**21b**	19.3 ± 5.8	24.8 ± 6.8	37.9 ± 3.9	51.9 ± 21.5	32.2 ± 11.3
9	**25**	24.5 ± 6.5	30.3 ± 6.6	34.4 ± 7.9	60.2 ± 14.0	27.7 ± 8.2
10	**26a**	25.4 ± 3.5	25.8 ± 7.8	32.9 ± 5.9	56.0 ± 12.6	30.1 ± 7.8
11	**26b**	21.1 ± 7.3	17.6 ± 4.2	24.9 ± 3.9	42.5 ± 7.3	41.2 ± 9.3
12	**31a**	11.0 ± 6.0	15.5 ± 5.1	24.1 ± 3.7	47.0 ± 10.1	36.4 ± 5.8
13	**31b**	10.3 ± 3.4	19.6 ± 4.2	31.5 ± 4.5	55.7 ± 13.0	31.5 ± 5.4
14	**39a**	17.9 ± 6.7	25.1 ± 6.7	33.3 ± 8.5	56.2 ± 13.7	30.5 ± 7.2
15	**39b**	12.0 ± 4.4	27.6 ± 6.5	43.9 ± 1.7	70.8 ± 4.9	24.8 ± 3.7
16	Hydroxytyrosol	3.7 ± 3.8	6.4 ± 2.0	15.4 ± 2.9	22.6 ± 3.7	55.0 ± 8.6

Abbreviation: DPPH, 2,2‐diphenyl‐1‐picryhydrazyl.

^a^ Results are expressed as mean ± SD of at least five repetitions.

^b^ *p *< 0.05 versus HT based on a one‐way ANOVA test, post hoc analysis was carried out, where appropriate, with the Bonferroni correction.

The second assay evaluates the ability of molecules to inhibit the activity of lipoxygenase. Initially, the inhibitory ability of HT was evaluated and revealed a slight inhibition up to 200 μM. Therefore, only the concentration of 200 μΜ in the derivatives was tested and the results are presented in Table 3. Compounds **21b** and **16b** seem to be better inhibitors than HT and revealed almost double inhibitory activity.

**Table 3:** Capacity of compounds to inhibit lipoxygenase.^a^

A/A	Compound	% Inhibition of lipoxygenase^a^	A/A	Compound	% Inhibition of lipoxygenase^a^
1	**5a**	NT	9	**25**	26.0 ± 9.8
2	**5b**	NT	10	**26a**	17.4 ± 4.1
3	**8**	19.3 ± 3	11	**26b**	11.4 ± 6.0
4	**16a**	23.3 ± 10.1	12	**31a**	15.2 ± 3.6
5	**16b**	41.4 ± 10.4	13	**31b**	25.2 ± 9.8
6	**16c**	31.0 ± 10.3	14	**39a**	22.6 ± 5.9
7	**21a**	26.4 ± 3.2	15	**39b**	27.7 ± 12.9
8	**21b**	38.6 ± 6.5	16	Hydroxytyrosol	19.9 ± 4.3

^a^ *p* < 0.05 versus HT based on a one‐way ANOVA test, post hoc analysis was carried out, where appropriate, with the Bonferroni correction.

## 3 CONCLUSION

A library of 16 novel HT analogs bearing substitutions at C‐1 of the HT aliphatic side chain was prepared. Their cytostatic and cytotoxic properties were studied in vitro using MG‐63 human osteoblast‐like cells. Compared with HT, two of the compounds exhibited significant inhibition of cell proliferation, underscoring the critical role of substitution on the aliphatic side chain. Notably, compounds bearing cyclohexyl and cyclopentyl groups on the α‐carbon of the aliphatic side chain were markedly more active than the mono‐ and dimethyl‐substituted HT derivatives. Additionally, the antioxidant potential of the new compounds was assessed through two assays: interaction with the DPPH free radical and inhibition of lipoxygenase activity. The cyclo‐substituted compounds again demonstrated superior radical scavenging abilities, showing higher activity in both assays compared with HT. These results highlight that the cyclo‐substituted HT analogs are not only more potent than HT but also hold significant promise for the development of novel antioxidants with potential applications in bone physiology.

## 4 EXPERIMENTAL

### 4.1 Chemistry

#### 4.1.1 General

All commercially available chemicals were purchased from Alfa Aesar. DPPH, soybean lipoxygenase (type I‐B), linoleic acid, and all other chemicals were purchased from Sigma‐Aldrich. Melting points were determined on a Büchi apparatus and were uncorrected. ^1^H NMR, ^13^C NMR, and two‐dimensional (2D) spectra (see the Supporting Information) were recorded on a Bruker Avance III 600, 400 spectrometer and 200 (Bruker GmbH) using dimethyl sulfoxide (DMSO*‐d*_*6*_), methanol (CD_3_OD), and chloroform (CDCl_3_) as deuterated solvents and were referenced to tetramethylsilane (*δ* scale). The signals of ^1^H and ^13^C spectra were unambiguously assigned by using 2D NMR techniques: ^1^H^1^H homonuclear correlation spectrosopy, heteronuclear single quantum coherence, and heteronuclear multiple bond correlation. Flash chromatography was performed on Merck silica gel 60 (0.040–0.063 mm). Analytical thin layer chromatography (TLC) was carried out on precoated (0.25 mm) Merck KgaA silica gel F‐254 plates. Mass spectra were recorded on a UPLC Triple TOF‐MS {UPLC: Acquity of Waters (USA), SCIEX Triple TOF‐MS 5600+ (USA)}.

The InChI codes of the investigated compounds, together with some biological activity data, are provided as Supporting Information.

#### 4.1.2 Synthesis of ethyl 3,4‐dimethoxyphenylacetate (**2**)

To a solution of 3,4‐dimethoxyphenylacetic acid (10.8 g, 55.05 mmol, **1**) in absolute EtOH (150 mL), H_2_SO_4_ 97% (4 mL) was added, and the reaction mixture was refluxed for 4 h. After completion of the reaction, the mixture was vacuum evaporated, extracted with CH_2_Cl_2_ (3 × 60 mL). The organic layer was collected, dried (anhydrous Na_2_SO_4_), and concentrated under reduced pressure to obtain the desired ester **2** (10.2 g), which was used without further purification for the next step.

#### 4.1.3 Synthesis of ethyl 2‐(3,4‐dimethoxyphenyl)propanoate (**3a**)

NaH (60% in mineral oil) (620 mg, 15.47 mmol) was added portion‐wise, at 0°C, to a solution of ester **2** (3.15 g, 14.06 mmol) in anhydrous dimethylformmide (DMF) (15 mL) under argon atmosphere. The resulting suspension was stirred at 0°C for 10 min and then at room temperature for 30 min. Subsequently, the suspension was cooled to 0°C; CH_3_I (0.88 mL, 14.06 mmol) was added and the reaction mixture was stirred at room temperature for 12 h. After completion of the reaction, the excess of NaH was quenched by the addition of methanol, and the resulting mixture was poured into water (80 mL) and extracted with EtOAc (3 × 40 mL). The organic layer was collected, dried over anhydrous sodium sulfate (Na_2_SO_4_), and evaporated to dryness. The oily residue was further purified by column chromatography (silica gel) using cyclohexane as the eluent to provide compound **3** (1.9 g). Oil.^[^ ^25^ ^]^ Yield: 56.7%. ^1^H NMR (600 MHz, CDCl_3_) *δ* (ppm): 6.86–6.82 (m, 2H, H‐2,6) 6.78 (d, *J* = 7.8 Hz, 1H, H‐5), 4.08 (q, *J* = 7.1 Hz, 2H, OC*H*_2_), 3.84 (s, 3H, 3‐OC*H*_3_), 3.82 (s, 3H, 4‐OC*H*_3_), 3.61 (q, *J* = 7.1 Hz, 1H, C*H*CO), 1.44 (d, *J* = 7.8 Hz, 3H, C*H*_3_CH), 1.17 (t, *J* = 7.1 Hz, 3H, C*H*_3_CH_2_). ^13^C NMR (151 MHz, CDCl_3_) *δ* (ppm): 174.39 (*C*O), 148.71 (C‐3), 147.76 (C‐4), 133.02 (C‐1), 119.29 (C‐6), 110.99 (C‐5), 110.44 (C‐2), 60.37 (O*C*H_2_), 55.58 (3,4‐O*C*H_3_), 44.81 (*C*HCO), 18.42 (*C*H_3_CH), 13.86 (*C*H_3_CH_2_).

#### 4.1.4 Synthesis of ethyl 2‐methyl‐2‐(3,4‐dimethoxyphenyl)propanoate (**3b**)

Compound **13c** was prepared in a procedure analogous to that used for the synthesis of derivative **3a**, using ester **2** as the starting material and 3 equivalents of CH_3_I and NaH. Oil. Yield: 60.4%. ^1^H NMR (600 MHz, CDCl_3_) *δ* (ppm): 6.89–6.84 (m, 2H, H‐2, 6) 6.79 (d, *J* = 7.9 Hz, 1H, H‐5), 4.09 (q, *J* = 7.1 Hz, 2H, OC*H*_2_), 3.84 (s, 3H, 3‐OC*H*_3_), 3.82 (s, 3H, 4‐OC*H*_3_), 1.54 (s, 6H, C*H*_3_C), 1.16 (t, *J* = 7.1 Hz, 3H, CH_2_C*H*_3_). ^13^C NMR (151 MHz, CDCl_3_) *δ* (ppm): 176.55 (*C*O), 148.58 (C‐3), 147.69 (C‐4), 137.32 (C‐1), 117.54 (C‐6), 110.83 (C‐5), 109.45 (C‐2), 60.58 (O*C*H_2_), 55.73 (3‐O*C*H_3_), 55.69 (4‐O*C*H_3_), 45.84 (CH_3_ *C*), 26.45 (*C*H_3_C), 13.96 (CH_2_ *C*H_3_).

#### 4.1.5 Synthesis of 2‐(3,4‐dimethoxyphenyl)propan‐1‐ol (**4a**)

A solution of ester **3a** (630 mg, 2.65 mmol) in anhydrous tetrahydrofurane (THF) (10 mL) was added dropwise, at 0°C, to a suspension of LiAlH_4_ (201.4 mg, 5.3 mmol) in anhydrous THF (15 mL), under argon atmosphere. The mixture was stirred at room temperature for 30 min. After completion of the reaction, excess LiAlH_4_ was quenched by the addition of 5% aqueous NaOH solution and water (50 mL), and the resulting mixture was extracted with EtOAc (3 × 20 mL). The organic layer was dried (anhydrous Na_2_SO_4_), concentrated in vacuo, and the resulting oily residue was purified by column chromatography (silica gel) using a mixture of cyclohexane/EtOAc 5/1 as the eluent to afford 430 mg of the desired alcohol **4a**. Oil.^[^ ^26^ ^]^ Yield: 82.7%. ^1^H NMR (600 MHz, CDCl_3_) *δ* (ppm): 6.79 (d, *J* = 7.8 Hz, 1H, H‐5), 6.72–6.69 (m, 2H, H‐2,6), 3.81 (s, 3H, 3‐OC*H*_3_), 3.78 (s, 3H, 4‐OC*H*_3_), 3.60– 3.54 (m, 2H, C*H*_2_OH), 2.85–2.79 (m, 1H, CH_3_C*H*), 1.19 (d, *J* = 7.8 Hz, 3H, C*H*_3_CH). ^13^C NMR (151 MHz, CDCl_3_) *δ* (ppm): 148.98 (C‐3), 147.67 (C‐4), 136.54 (C‐1), 119.23 (C‐6), 111.45 (C‐5), 110.89 (C‐2), 68.58 (*C*H_2_OH), 55.88 (3‐O*C*H_3_), 55.81 (4‐O*C*H_3_), 42.19 (CH_3_ *C*H), 17.79 (*C*H_3_CH).

#### 4.1.6 Synthesis of 2‐methyl‐2‐(3,4‐dimethoxyphenyl)‐propan‐1‐ol (**4b**)

Compound **13c** was prepared in a procedure analogous to that used for the synthesis of derivative **4a**, using ester **3b** as the starting material. Pale yellow solid.^[^ ^27^ ^]^ Yield: 90.8%. Mp.: 69–70°C (CHCl_3_/*n*‐pentane). ^1^H NMR (600 MHz, CDCl_3_) *δ* (ppm): 6.95–6.91 (m, 2H, H‐2, 6), 6.85 (d, *J* = 7.9 Hz, 1H, H‐5), 3.90 (s, 3H, 3‐OC*H*_3_), 3.88 (s, 3H, 4‐OC*H*_3_), 3.59 (s, 2H, C*H*_2_OH), 1.34 (s, 6H, C*H*_3_C). ^13^C NMR (151 MHz, CDCl_3_) δ (ppm): 148.80 (C‐3), 147.48 (C‐4), 138.96 (C‐1), 118.49 (C‐6), 110.99 (C‐5), 109.98(C‐2), 73.14 (*C*H_2_OH), 55.92 (3‐O*C*H_3_), 55.88 (4‐O*C*H_3_), 39.78 (CH_3_ *C*), 25.49 (*C*H_3_C).

#### 4.1.7 Synthesis of 4‐(1‐hydroxypropan‐2‐yl)‐benzene‐1,2‐diol (**5a**)

A mixture of alcohol **4a** (370 mg, 1.89 mmol) and pyridine hydrochloride (4.37 g, 37.80 mmol) was heated at 170°C under argon for 2 h. After completion of the reaction, the resulting mixture was treated with 10 mL of water and washed with EtOAc (3 × 15 mL). The organic layer was collected, dried (anhydrous Na_2_SO_4_), and concentrated under reduced pressure. The residue was purified by column chromatography (silica gel) using a mixture of cyclohexane/EtOAc 3/1 as the eluent to afford 60 mg of compound **5a**. Oil.^[^ ^28^ ^]^ Yield: 19%. ^1^H NMR (600 MHz, CDCl_3_) *δ* (ppm): 6.69 (d, *J* = 8.1 Hz, 1H, H‐5), 6.66 (d, *J* = 2.1 Hz, 1H, H‐2), 6.54 (dd, *J* = 8.1, 2.1 Hz, 1H, H‐6), 3.61–3.58 (m, 1H, C*H*_2_OH), 3.49–3.45 (m, 1H, C*H*_2_OH), 2.74– 2.68 (m, 1H, CH_3_C*H*), 1.20 (d, *J* = 7.8 Hz, 3H, C*H*_3_CH). ^13^C NMR (151 MHz, MeOD) δ (ppm): 144.70 (C‐3), 143.15 (C‐4), 135.98 (C‐1), 118.07 (C‐6), 114.81 (C‐5), 113.96 (C‐2), 67.87 (*C*H_2_OH), 41.63 (CH_3_ *C*H), 17.02 (*C*H_3_CH). (+) ESI QqToF (*m/z*): Calcd. for C_9_H_12_O_3_^+^: [M + H]+ 168.0786, found 168.0784.

#### 4.1.8 Synthesis of 4‐(1‐hydroxy‐2‐methylpropan‐2‐yl)benzene‐1,2‐diol (**5b**)

Compound **13c** was prepared in a procedure analogous to that used for the synthesis of derivative **5a**, using ester **4b** as the starting material. Oil: Yield: 18.5%. ^1^H NMR (600 MHz, MeOD) *δ* (ppm): 6.83 (dd, *J* = 2.0 Hz, 1H, H‐2), 6.69 (d, *J* = 7.9 Hz, 2H, H‐5, H‐6), 3.48 (s, 2H, C*H*_2_OH), 1.23 (s, 6H, C*H*_3_C). ^13^C NMR (151 MHz, MeOD) *δ* (ppm): 145.74 01 (C‐3), 144.15 (C‐4), 140.42 (C‐1), 118.51 (C‐6), 115.98 (C‐5), 114.77 (C‐2), 73.35 (CH_2_OH), 40.14 (CH_3_C), 25.97(CH_3_C). (+) ESI QqToF (*m/z*): Calcd. for C_10_H_14_O_3_^+^: [M + H]+ 182.0943, found 182.0946.

#### 4.1.9 Synthesis of ethyl 1‐(3,4‐dimethoxyphenyl)cyclopentane‐1‐carboxylate (**6**)

Compound **13c** was prepared in a procedure analogous to that used for the synthesis of derivative **3a**, using 1,4‐dibromobutane as the starting material and 3 equivalents NaH. Oil.^[^ ^29^ ^]^ Yield: 50.4% ^1^H NMR (600 MHz, CDCl_3_) δ (ppm): 6.92 (dd, *J* = 8.3, 2.1 Hz, 1H, H‐6), 6.89 (d, *J* = 2.1 Hz, 1H, H‐2), 6.80 (d, *J* = 8.3 Hz, 1H, H‐5), 4.07 (q, *J* = 7.1 Hz, 2H, OC*H*_2_), 3.87 (s, 3H, 3‐OC*H*_3_), 3.86 (s, 3H, 4‐OC*H*_3_), 2.67–2.59 (m, 2H, H‐2′, 5′), 1.92–1.82 (m, 2H, H‐2′, 5′), 1.79–1.65 (m, 4H, H‐3′, 4′), 1.16 (t, *J* = 7.1 Hz, 3H, CH_2_C*H*_3_). ^13^C NMR (151 MHz, CDCl_3_) *δ* (ppm): 176.24 (*C*O), 148.81 (C‐3), 147.92 (C‐4), 136.31 (C‐1), 119.00 (C‐6), 110.89 (C‐5), 110.65 (C‐2), 60.90 (O*C*H_2_), 58.77 (C‐1′), 55.97 (3‐O*C*H_3_), 55.91 (4‐O*C*H_3_), 36.27 (C‐2′, 5′), 23.62 (C‐3′, 4′), 14.14 (CH_2_ *C*H_3_).

#### 4.1.10 Synthesis of [1‐(3,4‐dimethoxyphenyl)cyclopentyl]methanol (**7**)

Compound **13c** was prepared in a procedure analogous to that used for the synthesis of derivative **4a**, using ester **6** as the starting material. Oil.^[^ ^29^ ^]^ Yield: 65.3%. ^1^H NMR (600 MHz, CDCl_3_) *δ* (ppm): 6.85 (dd, *J* = 8.2, 2.1 Hz, 1H, H‐6), 6.83–6.80 (m, 2H, H‐2, 5), 3.87 (s, 3H, 3‐OC*H*_3_), 3.85 (s, 3H, 4‐OC*H*_3_), 3.49 (s, 2H, C*H*_2_OH), 2.01–1.94 (m, 2H, H‐2′, 5′), 1.89–1.81 (m, 2H, H‐2′, 5′), 1.76–1.67 (m, 4H, H‐3′, 4′). ^13^C NMR (151 MHz, CDCl_3_) δ (ppm): 148.76 (C‐3), 147.40 (C‐4), 139.26 (C‐1), 119.18 (C‐6), 110.93 (C‐5), 110.80 (C‐2), 70.27 (*C*H_2_OH), 55.84 (3 ‐ O*C*H_3_), 55.81 (4 ‐O*C*H_3_), 52.92 (C‐1′), 34.47 (C‐2′, 5′), 23.81(C‐3′, 4′).

#### 4.1.11 Synthesis of 4‐[1‐(hydroxymethyl)cyclopentyl]benzene‐1,2‐diol (**8**)

BBr_3_ (1.1 mL, 1.1 mmol, 1 M in Hexanes) was added dropwise at –40°C to a solution of alcohol **7** (100 mg, 0.44 mmol) in anhydrous CH_2_Cl_2_ (8 mL) and the mixture was stirred at –40°C for 4 h. The reaction was then quenched with saturated NaHCO_3_, extracted with EtOAc (3 × 15 mL), and the organic layer was dried over anh. Na_2_SO_4_ and evaporated to dryness. The residue was purified by column chromatography (silica gel), using a mixture of cyclohexane/EtOAc 5/1 as the eluent, to afford 20 mg of the title compound **8**. Pale yellow solid. Yield: 21.8%. M.p.: 113–114°C (EtOAc). ^1^H NMR (600 MHz, MeOD) *δ* (ppm): 6.81 (d, *J* = 2.2 Hz, 1H, H‐2), 6.72 (d, *J* = 8.2 Hz, 1H, H‐5), 6.67 (dd, *J* = 8.2, 2.2 Hz, 1H, H‐6), 3.50 (s, 2H, C*H*_2_OH), 1.96–1.90 (m, 2H, H‐2′, 5′), 1.87–1.81 (m, 2H, H‐2′, 5′), 1.77–1.65 (m, 4H, H‐3′, 4′). ^13^C NMR (151 MHz, MeOD) *δ* (ppm): 145.64 (C‐3), 144.07 (C‐4), 140.44 (C‐1), 119.52 (C‐6), 115.88 (C‐5), 115.76 (C‐2), 70.76 (*C*H_2_OH), 53.57 (C‐1′), 35.65 (C‐2′, 5′), 24.86 (C‐3′, 4′). (+) ESI QqToF (*m/z*): Calcd. for C_12_H_16_O_3_^+^: [M + H]+ 208.1099, found 208.1095.

#### 4.1.12 Synthesis of 1,2‐bis(benzyloxy)benzene (**10**)

A suspension of catechol (110.11 mg, 1 mmol, **9**), potassium carbonate (580.46 mg, 4.2 mmol), and benzyl bromide (329.72 mg, 2.1 mmol) in acetone (50 mL) was stirred under reflux for 12 h. After completion of the reaction, the resulting mixture was filtered through a celite pad, and the filtrate was evaporated to dryness. The residue was purified by column chromatography on silica gel, using cyclohexane as the eluent, to afford 262.8 mg of the title compound **10**.^[^ ^30^ ^]^ Oil. Yield: 90.5%. ^1^H NMR (600 MHz, CDCl_3_) *δ* (ppm): 7.50 (d, 4H, H‐2′, 6′, 2”, 6”), 7.41 (t, *J* = 7.3 Hz, 4H, H‐3′, 5′, 3”, 5”), 7.35 (t, *J* = 7.3 Hz, 2H, H‐4′, 4”), 7.02–6.99 (m, 2H, H‐4, 5), 6.95–6.91 (m, 2H, H‐3, 6), 5.21 (s, 4H, 1, 2‐OC*H*_2_). ^13^C NMR (151 MHz, CDCl_3_) *δ* (ppm): 149.13 (C‐1, 2), 137.45 (C‐1′, 1”), 128.49 (C‐3′, 5′, 3”,5”), 127.78 (C‐4′, 4”), 127.34 (C‐2′, 6′, 2”, 6”), 121.70 (C‐4, 5), 115.37 (C‐3, 6), 71.37 (1, 2 ‐O*C*H_2_).

#### 4.1.13 Synthesis of {[(4‐bromo‐1,2‐phenylene)bis(oxy)]bis(methylene))}dibenzene (**11**)

A mixture of compound **10** (3.53 g, 12.17 mmol) and N‐bromosuccinimide (NBS) (2.31 g, 13 mmol) in acetonitrile (ACN) (70 mL) was stirred at room temperature for 2.5 h. After completion of the reaction, the volatiles were removed under reduced pressure. The residue was dissolved in dichloromethane (100 mL), washed with aqueous sodium thiosulfate (Na_2_S_2_O_3_) 5% and water (3 × 40 mL), dried over anhydrous Na_2_SO_4_ and concentrated to dryness. The residue was recrystallized from cyclohexane to afford 4.1 g of the title bromide **11**.^[^ ^31^ ^]^ Oil. Yield: 91.2%. ^1^H NMR (600 MHz, CDCl_3_) *δ* (ppm): 7.48 (d, *J* = 7.0 Hz, 2H, H‐2′, 6′), 7.45 (d, *J* = 7.0 Hz, 2H, H‐2”, 6”), 7.43–7.32 (m, 6H, H‐3′, 5′, 3”, 5”, 4”, 4”), 7.11 (d, *J* = 2.3 Hz, 1H, H‐3), 7.03 (dd, *J* = 8.6, 2.3 Hz, 1H, H‐5), 6.82 (d, *J* = 8.6 Hz, 1H, H‐6), 5.16 (s, 4H, OC*H*_2_). ^13^C NMR (151 MHz, CDCl_3_) *δ* (ppm): 149.81 (C‐2), 148.13 (C‐1), 136.83 (C‐1′), 136.55 (C‐1”), 128.50 (C‐3′, 5′), 128.46 (C‐3”, 5”), 127.95 (C‐4′), 127.86 (C‐4”), 127.29 (C‐2′, 6′), 127.23 (C‐2”, 6”), 124.14 (C‐5), 118.15 (C‐6), 116.49 (C‐3), 113.40 (C‐4), 71.46 (2‐O*C*H_2_), 71.36 (1‐O*C*H_2_).

#### 4.1.14 Synthesis of 1‐(3,4‐bis(benzyloxy)phenyl)cyclobutan‐1‐ol (**12a**)

A solution of *n*‐butyllithium (10.86 mL, 17.37 mmol) was added dropwise to a solution of bromide **11** (4.76 g, 13.03 mmol) in dry THF (90 mL) at –78°C and the resulting mixture was stirred for 15 min. Then, a solution of cyclobutanone (1.95 mL, 26.06 mmol) in dry THF (5 mL) was added dropwise and the resulting mixture was stirred at –78°C for 2 h. After completion of the reaction, the mixture was left to warm at 0°C, saturated ammonium chloride solution was added, and the mixture was stirred for 20 min at room temperature. The residue was extracted with EtOAc (3 × 40 mL), dried over anh. Na_2_SO_4_ and the organic layer was concentrated under reduced pressure. The residue was purified by column chromatography (silica gel), using a mixture of cyclohexane/EtOAc 50/1 as the eluent, to afford 4.1 g of the title alcohol **12a**, which was used directly for the next step, due to its instability. ^1^H NMR (600 MHz, CDCl_3_): *δ* (ppm): 7.51–7.47 (m, 4H), 7.39 (m, 4H), 7.35–7.31 (m, 2H), 7.15 (d, *J* = 2.2 Hz, 1H), 7.03 (dd, *J* = 8.4, 2.2 Hz, 1H), 6.96 (d, *J* = 8.4 Hz, 1H), 5.20 (s, 2H), 5.19 (s, 1H), 2.54–2.47 (m, 2H), 2.38–2.29 (m, 2H), 2.19–2.11 (m, 1H), 2.02–1.96 (m, 1H).

#### 4.1.15 Synthesis of 1‐[3,4‐(dibenzyloxy)phenyl]cyclohexanol (**12b**)

Compound **12b** was prepared in a procedure analogous to that used for the synthesis of derivative **12a**, using cyclohexanone as the starting material. Oil.^[^ ^32^ ^]^ Yield: 78.3%. ^1^H NMR (600 MHz, CDCl_3_) *δ* (ppm): 7.51–7.46 (m, 4H), 7.39 (, *J* = 7.3 Hz, 4H), 7.35–7.31 (t, *J* = 7.3 Hz, 2H), 7.21 (d, *J* = 2.2 Hz, 1H), 7.03 (dd, *J* = 8.4, 2.2 Hz, 1H), 6.94 (d, *J* = 8.4 Hz, 1H), 5.20 (s, 2H), 5.18 (s, 2H), 1.83–1.72 (m, 7H), 1.67–1.60 (m, 2H), 1.32–1.27 (m, 1H).

#### 4.1.16 Synthesis of 1‐[3,4‐(dibenzyloxy)phenyl]cycloheptanol (**12c**)

This compound was prepared by an analogous procedure as described for the synthesis of derivative **12a**, using cycloheptanone as the starting material. Oil. Yield: 82.5%. ^1^H NMR (600 MHz, CDCl_3_) *δ* (ppm): 7.50–7.46 (m, 4H), 7.42–7.37 (m, 4H), 7.36–7.31 (m, 2H), 7.17 (d, *J* = 2.2 Hz, 1H), 7.01 (dd, *J* = 8.4, 2.2 Hz, 1H), 6.92 (d, *J* = 8.2 Hz, 1H), 5.20 (s, 2H), 5.18 (s, 2H), 2.05–1.98 (m, 2H), 1.91–1.84 (m, 2H), 1.81–1.68 (m, 4H), 1.59–1.51 (m, 4H).

#### 4.1.17 Synthesis of 1‐[3,4‐bis(benzyloxy)phenyl]cyclobutane‐1‐carbonitrile (**13a**)

A mixture of trimethylsilyl cyanide (TMSCN) (2.85 mL, 22.78 mmol), indium(III) chloride (252 mg, 1.14 mmol), and alcohol **12a** (4.1 g, 11.39 mmol) in dry CH_2_Cl_2_ (20 mL) was stirred at room temperature for 2 h. After completion of the reaction, the mixture was quenched with water (70 mL) and stirred at room temperature for 20 min, extracted with CH_2_Cl_2_ (3 × 30 mL); the organic layers were collected, dried over anh. Na_2_SO_4,_ and evaporated to dryness. The residue was purified by column chromatography (silica gel), using a mixture of cyclohexane/EtOAc 27/1 as the eluent, to afford 2.92 g of the title compound **13a**. White solid. Yield: 69.5%. M.p.: 88–89°C (EtOAc). ^1^H NMR (600 MHz, CDCl_3_) *δ* (ppm): 7.55 (d, *J* = 7.0 Hz, 2H, H‐2”, 6”), 7.52 (d, *J* = 7.0 Hz, 2H, H‐2”’, 6”’), 7.47–7.42 (m, 4H, H‐3”, 5”, 3”’, 5”’), 7.41–7.35 (m, 2H, H‐4”, 4”’), 7.08 (s, 1H, H‐2), 7.03–6.97 (m, 2H, Η‐5, 6), 5.25 (s, 2H, 3‐OC*H*_2_), 5.22 (s, 2H, 4‐OC*H*_2_), 2.85–2.78 (m, 2H, Η‐2′, 4′), 2.60–2.53 (m, 2H, Η‐2′, 4′), 2.47–2.38 (m, 1H, Η‐3′), 2.10–2.01 (m, 1H, Η‐3′). ^13^C NMR (151 MHz, CDCl_3_) *δ* (ppm): 149.19 (C‐3), 148.80 (C‐4), 137.16 (C‐1”), 137.09 (C‐1”’), 133.05 (C‐1), 128.61 (C‐3”, 5”, 3”’, 5”’), 128.04 (C‐4”), 127.97 (C‐4”’), 127.62 (C‐2”, 6”), 127.36 (C‐2”’, 6”’), 124.60 (*C*N), 118.79 (C‐6), 115.05 (C‐5), 113.22 (C‐2), 71.69 (3 ‐ O*C*H_2_), 71.33 (4 ‐ O*C*H_2_), 39.88 (C‐1′), 34.78 (C‐2′, 4′), 17.03 (C‐3′).

#### 4.1.18 Synthesis of 1‐[3,4‐bis(benzyloxy)phenyl]cyclohexane‐1‐carbonitrile (**13b**)

Compound **13b** was prepared in a procedure analogous to that used for the synthesis of derivative **13a**, using **12b** as the starting material. Pale yellow solid. Yield: 42.4%. Mp.: 90–91°C (c‐Hex).^[^ ^32^ ^]^^1^H NMR (600 MHz, CDCl_3_) δ (ppm): 7.51–7.43 (m, 4H, H‐2”, 6”, 2”’, 6”’), 7.40 (t, *J* = 7.5 Hz, 4H, H‐3”, 5”, 3”’, 5”’), 7.34 (t, *J* = 7.3 Hz, 2H, H‐4”, 4”’), 7.11 (d, *J* = 2.1 Hz, 1H, H‐2), 7.01 (dd, *J* = 8.4, 2.1 Hz, 1H, H‐6), 6.95 (d, *J* = 8.4 Hz, 1H, H‐5), 5.20 (s, 2H, 3‐OC*H*_2_), 5.18 (s, 2H, 4‐OC*H*_2_), 2.14‐2.09 (m, 2H, H‐2′, 6′), 1.90 – 1.78 (m, 5H, H‐3′, 4′, 5′), 1.72–1.64 (m, 2H, H‐2′, 6′), 1.32–1.22 (m, 1H, H‐4′). ^13^C NMR (151 MHz, CDCl_3_) *δ* (ppm): 148.92 (C‐3), 148.70 (C‐4), 137.13 (C‐1”), 137.05 (C‐1”’), 134.75 (C‐1), 128.53 (C‐3”, 5”), 128.51 (C‐3”’, 5”’), 127.93 (C‐4”), 127.87 (C‐4”’), 127.59 (C‐2”, 6”), 127.27 (C‐2”’, 6”’), 122.84 (*C*N), 118.59 (C‐6), 114.90 (C‐5), 113.55 (C‐2), 71.75 (3‐O*C*H_2_), 71.28 (4‐O*C*H_2_), 43.73 (C‐1′), 37.44 (C‐2′, 6′), 25.00 (C‐4′), 23.61 (C‐3′, 5′).

#### 4.1.19 Synthesis of 1‐[3,4‐bis(benzyloxy)phenyl]cycloheptane‐1‐carbonitrile (**13c**)

Compound **13c** was prepared in a procedure analogous to that used for the synthesis of derivative **13a**, using **12c** as the starting material. Oil. Yield: 71.4%. ^1^H NMR (600 MHz, CDCl_3_) *δ* (ppm): 7.50–7.45 (m, 4H, H‐2”, 6”, 2”’, 6”’), 7.43–7.37 (m, 4H, H‐3”, 5”, 3”’, 5”’), 7.36–7.32 (m, 2H, H‐4”, 4”’), 7.09 (d, *J* = 2.4 Hz, 1H, H‐2), 7.02 (dd, *J* = 8.2, 2.4 Hz, 1H, H‐6), 6.93 (d, *J* = 8.2 Hz,1H, H‐5), 5.20 (s, 1H, 3‐OC*H*_2_), 5.18 (s, 1H, 4‐OC*H*_2_), 2.19–2.16 (m, 2H, H‐2′, 7′), 1.97–1.90 (m, 2H, H‐2′, 7′), 1.89–1.77 (m, 6H, H‐3′, 4′, 5′, 6′), 1.67–1.59 (m, 2H, H‐4′, 5′). ^13^C NMR (151 MHz, CDCl_3_) δ (ppm): 148.71 (C‐3), 148.37 (C‐4), 137.06 (C‐1”), 136.99 (C‐1”’), 136.21 (C‐1), 128.44 (C‐3”, 5”, 3”’, 5”’), 127.84 (C‐4”), 127.79 (C‐4”’), 127.51 (C‐2”, 6”), 127.20 (C‐2”’,6”’), 123.64 (*C*N), 118.31 (C‐6), 114.77 (C‐5), 113.35 (C‐2), 71.66 (3‐O*C*H_2_), 71.13 (4‐O*C*H_2_), 46.98 (C‐1′), 40.70 (C‐2′, 7′), 27.33 (C‐4′, 5′), 23.97 (C‐3′, 6′).

#### 4.1.20 Synthesis of 1‐[3,4‐bis(benzyloxy)phenyl]cyclobutane‐1‐carbaldehyde (**14a**)

A solution of diisobutylaluminum hydride (DIBAL) (1 M in CH_2_Cl_2_, 2.7 mL) was added dropwise to a solution of carbonitrile **13a** (430 mg, 1.1 mmol) in dry CH_2_Cl_2_ (30 mL) at –78°C and the resulting mixture was stirred for 1 h. After completion of the reaction, the mixture was left to warm at room temperature, quenched with saturated NH_4_Cl solution (5 mL) and methanol (5 mL), and stirred at room temperature. After 2 h, the mixture was extracted with CH_2_Cl_2_ (3 × 25 mL); the organic layers were collected, dried over anh. Na_2_SO_4,_ and evaporated to dryness. The residue was purified by column chromatography (silica gel), using a mixture of cyclohexane/EtOAc 20/1 as the eluent, to afford 280 mg of the title aldehyde **14a**. Pale yellow solid. Yield: 63.6%. M.p.: 75–76°C (EtOAc). ^1^H NMR (600 MHz, CDCl_3_) *δ* (ppm): 9.35 (s, 1H, C*H*O), 7.54–7.49 (m, 4H, H‐2”, 6”, 2”’, 6”’), 7.46–7.40 (m, 4H, H‐3”, 5”, 3”’, 5”’), 7.39– 7.35 (m, 2H, H‐4”, 4”’), 7.00 (d, *J* = 8.4 Hz, 1H, H‐5), 6.98 (d, *J* = 2.0 Hz, 1H, H‐2), 6.92 (dd, *J* = 8.4, 2.0 Hz, 1H, H‐6), 5.22 (s, 2H, 3‐OC*H*_2_), 5.21 (s, 2H, 4‐OC*H*_2_), 2.33–2.24 (m, 2H, H‐2′, 6′), 1.85–1.78 (m, 2H, H‐3′, 5′), 1.73–1.60 (m, 3H, H‐3′, 4′, 5′), 1.56–1.45 (m, 2H, H‐2′, 6′), 1.41–1.31 (m, 1H, H‐4′). ^13^C NMR (151 MHz, CDCl_3_): *δ* (ppm) 202.08 (*C*HO), 149.02 (C‐3), 148.50 (C‐4), 137.30 (C‐1”), 137.27 (C‐1”’), 132.74 (C‐1), 128.57 (C‐3”, 5”), 128.54 (C‐3”’, 5”’), 127.93 (C‐4”), 127.89 (C‐4”’), 127.62 (C‐2”, 6”), 127.33 (C‐2”’, 6”’), 120.53 (C‐6), 115.06 (C‐2), 114.98 (C‐5), 71.73 (3 ‐ O*C*H_2_), 71.36 (4 ‐ O*C*H_2_), 53.85 (C‐1′), 31.34 (C‐2′, 6′), 25.69 (C‐4′), 22.88 (C‐3′, 5′).

#### 4.1.21 Synthesis of 1‐[3,4‐bis(benzyloxy)phenyl]cyclohexane‐1‐carbaldehyde (**14b**)

Compound **14b** was prepared in a procedure analogous to that used for the synthesis of derivative **14a**, using **13b** as the starting material. Pale yellow solid. Yield: 63.6%. Mp.: 75–76°C (EtOAc).^[^ ^32^ ^]^^1^H NMR (600 MHz, CDCl_3_) δ (ppm): 9.35 (s, 1H, C*H*O), 7.54–7.49 (m, 4H, H‐2”,6”,2”’,6”’), 7.46 ‐ 7.40 (m, 4H, H‐3”, 5”, 3”’, 5”’), 7.39–7.35 (m, 2H, H‐4”, 4”’), 7.00 (d, *J* = 8.4 Hz, 1H, H‐5), 6.98 (d, *J* = 2.0 Hz, 1H, H‐2), 6.92 (dd, *J* = 8.4 Hz, 2.0 Hz, 1H, H‐6), 5.22 (s, 2H, 3‐OC*H*_2_), 5.21 (s, 2H, 4‐OC*H*_2_), 2.33–2.24 (m, 2H, H‐2′, 6′), 1.85–1.78 (m, 2H, H‐3′, 5′), 1.73–1.60 (m, 3H, H‐3′, 4′, 5′), 1.56–1.45 (m, 2H, H‐2′, 6′), 1.41–1.31 (m, 1H, H‐4′). ^13^C NMR (151 MHz, CDCl_3_) δ (ppm): 202.08 (*C*HO), 149.02 (C‐3), 148.50 (C‐4), 137.30 (C‐1”), 137.27 (C‐1”’), 132.74 (C‐1), 128.57 (C‐3”, 5”), 128.54 (C‐3”’, 5”’), 127.93 (C‐4”), 127.89 (C‐4”’), 127.62 (C‐2”, 6”), 127.33 (C‐2”’, 6”’), 120.53 (C‐6), 115.06 (C‐2), 114.98 (C‐5), 71.73 (3‐O*C*H_2_), 71.36 (4‐O*C*H_2_), 53.85 (C‐1′), 31.34(C‐2′, 6′), 25.69 (C‐4′), 22.88 (C‐3′, 5′).

#### 4.1.22 Synthesis of 1‐[3,4‐bis(benzyloxy)phenyl]cycloheptane‐1‐carbaldehyde (**14c**)

Compound **14c** was prepared in a procedure analogous to that used for the synthesis of derivative **14a**, using **13c** as the starting material. Oil. Yield: 87.7%. ^1^H NMR (600 MHz, CDCl_3_) δ (ppm): 9.29 (s, 1H, C*H*O), 7.50–7.46 (m, 4H, H‐2”, 6”, 2”’, 6”’), 7.43–7.38 (m, 4H, H‐3”, 5”, 3”’, 5”’), 7.37–7.33 (m, 2H, H‐4”, 4”’), 6.96 (d, *J* = 8.4 Hz, 1H, H‐5), 6.85 (d, *J* = 2.2 Hz, 1H, H‐2), 6.83 (dd, *J* = 8.4, 2.2 Hz, 1H, H‐6), 5.19 (s, 4H, 3,4‐OC*H*_2_), 2.21–2.14 (m, 2H, H‐2′, 7′), 2.04– 1.98 (m, 2H, H‐2′, 7′), 1.67–1.48 (m, 8H, H‐3′, 4′, 5′, 6′). ^13^C NMR (151 MHz, CDCl_3_) *δ* (ppm): 201.48 (*C*HO), 148.95 (C‐3), 148.35 (C‐4), 137.34 (C‐1”), 137.31 (C‐1”’), 134.06 (C‐1), 128.62 (C‐3”, 5”), 128.59 (C‐3”’, 5”’), 127.96 (C‐4”), 127.94 (C‐4”’), 127.63 (C‐2”, 6”), 127.38 (C‐2”’, 6”’), 120.73 (C‐6), 115.50 (C‐2), 114.97 (C‐5), 71.82 (3‐O*C*H_2_), 71.30 (4‐O*C*H_2_), 57.33 (C‐1′), 33.03 (C‐2′, 7′), 29.39 (C‐4′, 5′), 23.37 (C‐3′, 6′).

#### 4.1.23 Synthesis of {1‐[3,4‐bis(benzyloxy)phenyl]cyclobutyl}methanol (**15a**)

To a solution of aldehyde **14a** (300 mg, 0.80 mmol) in abs. methanol (25 mL), at 0°C, NaBH_4_ (56.75 mg, 1.5 mmol) was added, and the mixture was stirred at room temperature for 2 h. After completion of the reaction, the mixture was acidified with aq. HCl 9%, poured into water (60 mL) and washed with EtOAc (3 × 25 mL). The organic layers were collected, dried over anh. Na_2_SO_4,_ and evaporated to dryness. The residue was purified by column chromatography (silica gel), using a mixture of cyclohexane/EtOAc 6/1 as the eluent, to afford 250 mg of the title alcohol **15a**. White solid. Yield: 83.5%. M.p.: 63–64°C (CH_2_Cl_2_/*n* ‐ Pentane). ^1^H NMR (600 MHz, CDCl_3_) *δ* (ppm): 7.50–7.46 (m, 4H, H‐2”, 6”, 2”’, 6”’), 7.42–7.37 (m, 4H, H‐3”, 5”, 3”’, 5”’), 7.36–7.32 (m, 2H, H‐4”, 4”’), 6.94 (d, *J* = 8.4 Hz, 1H, H‐5), 6.75 (d, *J* = 2.1 Hz, 1H, H‐2), 6.69 (dd, *J* = 8.4, 2.1 Hz, 1H, H‐6), 5.19 (s, 2H, 3 ‐OC*H*_2_), 5.18 (s, 2H, 4‐OC*H*_2_), 3.69 (s, 2H, C*H*_2_OH), 2.31–2.23 (m, 2H, Η‐2′, 4′), 2.22–2.16 (m, 2H, Η‐2′, 4′), 2.11–2.02 (m, 1H, Η‐3′), 1.93–1.85 (m, 1H, Η‐3′). ^13^C NMR (151 MHz, CDCl_3_) *δ* (ppm): 148.87 (C‐3), 147.61 (C‐4), 140.98 (C‐1), 137.59 (C‐1”), 137.43 (C‐1”’), 128.59 (C‐3”, 5”, 3”’, 5”’), 127.96 (C‐4”), 127.89 (C‐4”’), 127.67 (C‐2”, 6”), 127.43 (C‐2”’, 6”’), 119.11 (C‐6), 115.20 (C‐5), 113.80 (C‐2), 71.63 (3‐O*C*H_2_), 71.61 (4‐O*C*H_2_), 70.97 (*C*H_2_OH), 47.64 (C‐1′), 29.70 (C‐2′, 4′), 15.85 (C‐3′).

#### 4.1.24 Synthesis of {1‐[3,4‐bis(benzyloxy)phenyl]cyclohexyl}methanol (**15b**)

Compound **15b** was prepared in a procedure analogous to that used for the synthesis of derivative **15a**, using **14b** as the starting material. White solid. Yield: 82.8% Mp.: 88–89°C (EtOAc).^[^ ^32^ ^]^^1^H NMR (600 MHz, CDCl_3_) δ (ppm): 7.52 (dd, *J* = 7.9, 0.9 Hz, 2H, H‐2”, 6”), 7.48 (dd, *J* = 7.9, 0.9 Hz, 2H, H‐2”’, 6”’), 7.45–7.38 (m, 4H, H‐3”, 5”, 3”’, 5”’), 7.37–7.32 (m, 2H, H‐4”, 4”’), 6.99–6.96 (m, 2H, H‐2, 5), 6.93 (dd, *J* = 8.5, 2.2 Hz, 1H, H‐6), 5.23 (s, 2H, 3‐OC*H*_2_), 5.20 (s, 2H, 4‐OC*H*_2_), 3.43 (s, 2H, C*H*_2_OH), 2.13–2.00 (m, 2H, H‐2′, 6′), 1.62–1.46 (m, 5H, H‐2′, 3′, 4′, 5′, 6′), 1.42–1.26 (m, 3H, H‐3′, 4′, 5′). ^13^C NMR (151 MHz, CDCl_3_) *δ* (ppm): 148.49 (C‐3), 147.57 (C‐4), 137.41 (C‐1”), 137.37 (C‐1”’), 136.79 (C‐1), 128.45 (C‐3”, 5”), 128.41 (C‐3”’, 5”’), 127.76 (C‐4”, 4”’), 127.52 (C‐2”, 6”), 127.27 (C‐2”’, 6”’), 120.66 (C‐6), 115.70 (C‐5), 114.76 (C‐2), 72.97 (*C*H_2_OH), 71.73 (3‐O*C*H_2_), 71.17 (4‐O*C*H_2_), 43.47 (C‐1′), 32.60 (C‐2′,6′), 26.53 (C‐4′), 21.91 (C‐3′, 5′).

#### 4.1.25 Synthesis of {1‐[3,4‐bis(benzyloxy)phenyl]cycloheptyl}methanol (**15c**)

Compound **15c** was prepared in a procedure analogous to that used for the synthesis of derivative **15a**, using **14c** as the starting material. Oil. Yield: 72.6%. ^1^H NMR (600 MHz, CDCl_3_) *δ* (ppm): 7.48 (d, *J* = 7.5 Hz, 2H, H‐2”, 6”), 7.44 (d, *J* = 7.5 Hz, 2H, H‐2”’, 6”’), 7.41–7.33 (m, 4H, H‐3”, 5”, 3”’, 5”’), 7.33–7.28 (m, 2H, H‐4”, 4”’), 6.94–6.90 (m, 2H, H‐2, 5), 6.88 (dd, *J* = 8.4, 2.3 Hz, 1H, H‐6), 5.19 (s, 2H, 3‐OC*H*_2_), 5.17 (s, 2H, 4‐OC*H*_2_), 3.39 (s, 2H, C*H*_2_OH), 2.07–1.99 (m, 2H, H‐2′, 7′), 1.71–1.62 (m, 2H, H‐2′, 7′), 1.61–1.48 (m, 4H, H‐3′, 6′), 1.47–1.38 (m, 4H, H‐4′, 5′). ^13^C NMR (151 MHz, CDCl_3_) δ (ppm): 148.48 (C‐3), 147.66 (C‐4), 138.65 (C‐1), 137.56 (C‐1”, 1”’), 128.61 (C‐3”, 5”), 128.57 (C‐3”’, 5”’), 127.91 (C‐4”, 4”’), 127.65 (C‐2”, 6”), 127.43 (C‐2”’, 6”’), 120.67 (C‐6), 115.92 (C‐5), 114.75 (C‐2), 73.29 (*C*H_2_OH), 71.89 (3‐O*C*H_2_), 71.34 (4‐O*C*H_2_), 46.66 (C‐1′), 35.19 (C‐2′, 7′), 29.86 (C‐4′, 5′), 23.31 (C‐3′, 6′).

#### 4.1.26 Synthesis of 4‐[1‐(hydroxymethyl)cyclobutyl]benzene‐1,2‐diol (**16a**)

A suspension of alcohol **15a** (450 mg, 1.20 mmol) in absolute ethanol (30 mL) was hydrogenated in the presence of 10% Pd/C (30 mg) under a pressure of 50 psi, at room temperature, for 4 h. The resulting mixture was filtered through a celite pad, and the filtrate was evaporated to dryness. The oily residue was purified by column chromatography (silica gel), using a mixture of cyclohexane/EtOAc 2/1 as the eluent, to afford 200 mg of the title compound **16a**. White solid. Yield: 85.8%. M.p.: 137–138°C (EtOAc). ^1^H NMR (600 MHz, MeOD) *δ* (ppm): 6.74 (d, *J* = 8.4 Hz, 1H, H‐5), 6.64 (d, *J* = 1.9 Hz, 1H, H‐2), 6.51 (dd, *J* = 8.4, 1.9 Hz, 1H, H‐6), 3.62 (s, 2H, C*H*_2_OH), 2.28–2.20 (m, 4H, Η‐2′, 4′), 2.06–1.97 (m, 1H, Η‐3′), 1.87–1.80 (m, 1H, Η‐3′). ^13^C NMR (151 MHz, MeOD) δ (ppm): 145.69 (C‐3), 144.00 (C‐4), 141.32 (C‐1), 118.41 (C‐6), 115.95 (C‐5), 114.49 (C‐2), 71.33 (*C*H_2_OH), 48.54 (C‐1′), 30.49 (C‐2′, 4′), 16.25 (C‐3′). (+) ESI QqToF (*m/z*): Calcd. for C11H14O3^+^: [M + H]+ 194.0943, found 194.0939.

#### 4.1.27 Synthesis of 4‐[1‐(hydroxymethyl)cyclohexyl]benzene‐1,2‐diol (**16b**)

Compound **16b** was prepared in a procedure analogous to that used for the synthesis of derivative **16a**, using **15b** as the starting material. White solid. Yield: 79%. Mp.: 137–138°C (EtOAc – n‐pentane). ^1^H NMR (600 MHz, MeOD) *δ* (ppm): 6.87 (d, *J* = 2.2 Hz, 1H, H‐2), 6.77 (d, *J* = 8.4 Hz, 1H, H‐5), 6.72 (dd, *J* = 8.4, 2.2 Hz, 1H, H‐6), 3.37 (s, 2H, C*H*_2_OH), 2.10–2.02 (m, 2H, H‐2′, 6′), 1.64–1.57 (m, 2H, H‐2′, 6′), 1.57–1.50 (m, 3H, H‐3′, 4′, 5′), 1.45– 1.29 (m, 3H, H‐3′, 4′, 5′). ^13^C NMR (151 MHz, MeOD) δ (ppm): ^3^C NMR (151 MHz, CDCl3) δ (ppm): 144.27, 142.34, 136.16, 119.45, 115.26, 114.54, 73.14, 43.44, 32.86, 29.82, 26.74, 22.14. (+) ESI QqToF (m/z): Calcd. for C_13_H_18_O_3_^+^: [M + H]+ 222.1256, found 222.1254.

#### 4.1.28 Synthesis of 4‐[1‐(hydroxymethyl)cycloheptyl]benzene‐1,2‐diol (**16c**)

Compound **16c** was prepared in a procedure analogous to that used for the synthesis of derivative **16a**, using **15c** as the starting material. Pale yellow solid. Yield: 82.2%. Mp.: 134–135°C (EtOAc– n‐pentane). ^1^H NMR (600 MHz, MeOD) *δ* (ppm): 6.82 (d, *J* = 2.2 Hz, 1H, H‐2), 6.72 (d, *J* = 8.4, 1H, H‐5), 6.67 (dd, *J* = 8.4, 2.2 Hz, 1H, H‐6), 3.38 (s, 2H, C*H*_2_OH), 2.09– 2.03 (m, 2H, H‐2′, 7′), 1.76–1.70 (m, 2H, H‐2′, 7′), 1.66–1.58 (m, 2H, H‐3′, 6′), 1.57–1.45 (m, 6H, H‐3′, 4′, 5′, 6′). ^13^C NMR (151 MHz, MeOD) δ (ppm): 145.75 (C‐3), 143.94 (C‐4), 139.16 (C‐1), 119.72 (C‐6), 115.98 (C‐5), 115.90 (C‐2), 74.41 (*C*H_2_OH), 47.23 (C‐1′), 36.35 (C‐2′, 7′), 30.83 (C‐4′, 5′), 24.50 (C‐3′, 6′). (+) ESI QqToF (*m/*z): Calcd. for C_14_H_20_O_3_^+^: [M + H]+ 236.1412, found 236.1416.

#### 4.1.29 Synthesis of 4‐(cyclopent‐1‐en‐1‐yl)‐1,2‐(dibenzyloxy)benzene (**18**)

Derivative **18** was obtained after spontaneous dehydration of alcohol **17** at room temperature, which was prepared in a procedure analogous to that used for the synthesis of derivative **12**, using cyclopentanone as the starting material. Pale yellow solid. Mp.: 76.5–78°C (EtOAc). ^1^H NMR (600 MHz, CDCl_3_) *δ* (ppm): 7.47 (d, *J* = 7.5 Hz, 2H, H‐2”, 6”), 7.45 (d, *J* = 7.5 Hz, 2H, H‐2”’, 6”’), 7.39–7.34 (m, 4H, H‐3”, 5”, 3”’, 5”’), 7.34–7.28 (m, 2H, H‐4”, 4”’), 7.09 (d, *J* = 2.0 Hz, 1H, H‐2), 6.95 (dd, *J* = 8.3, 2.0 Hz, 1H, H‐6), 6.88 (d, *J* = 8.3 Hz, 1H, H‐5), 6.02–5.99 (m, 1H, H‐2′), 5.17 (s, 2H, 3‐OC*H*_2_), 5.16 (s, 2H, 4‐OC*H*_2_), 2.66–2.61 (m, 2H, H‐5′), 2.53–2.48 (m, 2H, H‐3′), 2.02–1.96 (m, 2H, H‐4′). ^13^C NMR (151 MHz, CDCl_3_) *δ* (ppm): 148.79 (C‐3), 148.25 (C‐4), 141.92 (C‐1′), 137.47 (C‐1”), 137.41 (C‐1”’), 130.85 (C‐1), 128.48 (C‐3”, 5”, 3”’, 5”’), 127.80 (C‐4”), 127.77 (C‐4”’), 127.43 (C‐2”, 6”), 127.32 (C‐2”’, 6”’), 124.66 (C‐2′), 119.01 (C‐6), 114.96 (C‐5), 112.98 (C‐2), 71.57 (3‐O*C*H_2_), 71.40 (4‐O*C*H_2_), 33.30 (C‐3′, 5′), 23.39 (C‐4′).

#### 4.1.30 Synthesis of 2‐[3,4‐(dihydroxy)phenyl]cyclopentanone (**20**)

To a solution of osmium tetroxide (490 μL, 2.5% w\t in t‐BuOH) and *N*‐methylmorpholine *N*‐oxide (490 mg, 4.20 mmol) in a mixture of t‐BuOH/Acetone/water (7.5 mL/7.5 mL/1.5 mL) was added olefin **19** (500 mg, 1.40 mmol) and the resulting mixture was stirred at room temperature for 16 h. A saturated NaHSO_3_ solution (5 mL) was then added, and the resulting mixture was stirred for 30 min. The reaction mixture was then extracted with EtOAc (3 × 40 mL); the organic phase was washed with water and brine, dried over anhydrous Na_2_SO_4,_ and concentrated to dryness. The oily residue was passed through a short column (silica gel, elution system c‐Hex/EtoAc 10/1) to afford 410 mg of cyclopentanediol **19**, which was dissolved in absolute EtOH (30 mL) and hydrogenated at a pressure of 50 psi in the presence of Pd/C (20 mg), at room temperature, for 4 h. The resulting mixture was filtered through a celite pad, and the filtrate was evaporated to dryness. The oily residue was purified by column chromatography (silica gel), using a mixture of cyclohexane/EtOAc 2/1 as the eluent, to afford 170 mg of the title compound **20**. Oil. Yield: 75.7%. ^1^H NMR (600 MHz, CDCl_3_) *δ* (ppm): 6.73 (d, *J* = 8.1 Hz, 1H, H‐5), 6.66 (d, *J* = 2.0 Hz, 1H, H‐2), 6.56 (dd, *J* = 8.1, 2.0 Hz, 1H, H‐6), 3.25–3.18 (m, 1H, H‐1′), 2.53–2.43 (m, 2H, H‐3′,5′), 2.33–2.25 (m, 1H, H‐3′), 2.18–2.11 (m, 1H, H‐4′), 2.09–2.00 (m, 1H, H‐5′), 1.96–1.86 (m, 1H, H‐4′). ^13^C NMR (151 MHz, CDCl_3_) δ (ppm): 220.57 (*C*O), 144.00 (C‐3), 142.96 (C‐4), 130.93 (C‐1), 120.64 (C‐6), 115.63 (C‐5), 115.39 (C‐2), 55.24 (C‐1′), 38.45 (C‐3′), 32.01 (C‐5′), 20.87 (C‐4′).

#### 4.1.31 Synthesis of 4‐(2‐hydroxycyclopentyl)benzene‐1,2‐diol (**21**)

To a solution of aldehyde **20** (170 mg, 0.45 mmol) in dry methanol (25 mL), NaBH_4_ (34.2 mg, 0.9 mmol) was added portion‐wise at 0°C. The resulting mixture was stirred at room temperature for 2 h. After completion of the reaction, the mixture was carefully acidified with 9% HCl, poured into water (60 mL), and washed with EtOAc (3 × 25 mL). The combined organic layers were dried over anhydrous Na_2_SO_4_ and evaporated to dryness. Preparative high‐performance liquid chromatography (HPLC) system (for the first 40 min, a H_2_O/ACN 90/10 to H_2_O/ACN 70/30 system was used as the mobile phase; for the next 5 min, the composition of the mobile phase remained constant, and finally, in the last 15 min, the solvent system was changed again gradually from H_2_O/ACN 70/30 to H_2_O/ACN 40/60) afforded 4‐[(1S,2 R)*‐2‐hydroxycyclopentyl]benzo‐1,2‐diol (**21a**) and 4‐[(1S,2S)*‐2‐hydroxycyclopentyl]benzo‐1,2‐diol (**21b**) in 1/3 ratio, respectively.

4‐[(1*S*,2 *R*)*‐2‐hydroxycyclopentyl]benzene‐1,2‐diol (**21a**): Oil. Yield: 32.7%. ^1^H NMR (600 MHz, MeOD) *δ* (ppm): 6.70 (d, *J* = 1.8 Hz, 1H, H‐2), 6.68 (d, *J* = 8.1 Hz, 1H, H‐5), 6.57 (dd, *J* = 8.1, 1.8 Hz, 1H, H‐6), 4.07–4.02 (m, 1H, H‐2′), 2.77–2.70 (m, 1H, H‐1′), 2.11–2.04 (m, 1H, H‐5′), 2.04–1.97 (m, 1H, H‐3′), 1.85–1.72 (m, 2H, H‐4′), 1.68–1.59 (m, 2H, H‐3′, 5′). ^13^C NMR (151 MHz, MeOD) δ (ppm): 146.15 (C‐3), 144.49 (C‐4), 136.98 (C‐1), 119.72 (C‐6), 116.24 (C‐5), 115.51 (C‐2), 81.04 (C‐2′), 54.65 (C‐1′), 35.19 (C‐3′), 33.37 (C‐5′), 22.93 (C‐4′). (+) ESI QqToF (*m/z*): Calcd. for C_11_H_14_O_3_^+^: [M + H]+ 194.0943, found 194.0945.

4‐[(1*S*,2*S*)*‐2‐hydroxycyclopentyl]benzene‐1,2‐diol (**21b**): White solid. Yield: 13%. Mp.: 106–107°C (EtOAc). ^1^H NMR (600 MHz, MeOD) δ (ppm): 6.77 (d, *J* = 1.9 Hz, 1H, H‐2), 6.70 (d, *J* = 8.1 Hz, 1H, H‐5), 6.63 (dd, *J* = 8.1, 1.9 Hz, 1H, H‐6), 4.20–4.15 (m, 1H, H‐2′), 2.89–2.80 (m, 1H, H‐1′), 2.03–1.94 (m, 2H, H‐3′, 5′), 1.94–1.83 (m, 2H, H‐4′, 5′), 1.78–1.71 (m, 1H, H‐3′), 1.70–1.64 (m, 1H, H‐4′). ^13^C NMR (151 MHz, MeOD) δ (ppm): 145.85 (C‐3), 144.55 (C‐4), 133.46 (C‐1), 121.11 (C‐6), 117.03 (C‐2), 116.03 (C‐5), 76.52 (C‐2′), 52.33 (C‐1′), 35.07 (C‐3′), 29.02 (C‐5′), 22.84 (C‐4′). (+) ESI QqToF (m/z): Calcd. for C_11_H_12_O_3_^+^: [M + H]+ 194.0943, found 194.0947.

#### 4.1.32 Synthesis of 4‐(cyclohex‐1‐en‐1‐yl)‐1,2‐(dibenzyloxy)benzene (**23**)

1‐(3,4‐bis(benzyloxy)phenyl)cyclohexan‐1‐ol **(22)** was prepared in a procedure analogous to that used for the synthesis of derivative **12**, using cyclohexanone as the starting material. Then, due to the instability of alcohol **22**, without any purification, a solution of **22** (100 g, 2.84 mmol) in THF (70 mL), p‐toluenesulfonic acid (53.9 mg, 0.28 mmol) was added and the reaction mixture was stirred at room temperature for 16 h. After completion of the reaction, the volatiles were removed under reduced pressure and the residue was extracted with EtOAc, washed with a saturated NaHCO_3_ solution, water, and brine, dried (anh. Na_2_SO_4_), and concentrated to dryness. The crude product was purified by column chromatography (silica gel), using a mixture of cyclohexane/EtOAc 55/1 as the eluent, to afford 750 mg of olefin **23**. Pale yellow solid. Yield: 71.5%. Mp.: 73–74°C (c‐Ηex). ^1^H NMR (600 MHz, CDCl_3_) δ (ppm): 7.56 (d, *J* = 7.5 Hz, 2H, H‐2”, 6”), 7.53 (d, *J* = 7.5 Hz, 2H, H‐2”’, 6”’), 7.48–7.42 (m, 4H, H‐3”, 5”, 3”’, 5”’), 7.41–7.36 (m, 2H, H‐4”, 4”’), 7.14 (d, *J* = 2.1 Hz, 1H, H‐2), 7.01 (dd, *J* = 8.4, 2.1 Hz, 1H, H‐6), 6.98 (d, *J* = 8.4 Hz, 1H, H‐5), 6.12–6.09 (m, 1H, H‐2′), 5.25 (s, 2H, 3‐OC*H*_2_), 5.23 (s, 2H, 4‐OC*H*_2_), 2.46–2.41 (m, 2H, H‐6′), 2.30–2.25 (m, 2H, H‐3′), 1.87–1.82 (m, 1H, H‐5′), 1.75–1.70 (m, 2H, H‐4′). ^13^C NMR (151 MHz, CDCl_3_) *δ* (ppm): 148.84 (C‐3), 148.10 (C‐4), 137.59 (C‐1”), 137.57 (C‐1”’), 136.62 (C‐1), 136.01 (C‐1′), 128.52 (C‐3”, 5”, 3”’, 5”’), 127.82 (C‐4”), 127.79 (C‐4”’), 127.51 (C‐2”, 6”), 127.40 (C‐2”’, 6”’), 123.72 (C‐2′), 118.16 (C‐6), 115.02 (C‐5), 112.60 (C‐2), 71.62 (3‐O*C*H_2_), 71.48 (4‐O*C*H_2_), 27.50 (C‐6′), 25.92 (C‐3′), 23.16 (C‐5′), 22.27 (C‐4′).

#### 4.1.33 Synthesis of 1‐[3,4‐(dibenzyloxy)phenyl]cyclohexane‐1,2‐diol (**24**)

Compound **24** was prepared in a procedure analogous to that used for the synthesis of derivative **19**, using compound **23** as the starting material. Pale yellow solid. Yield: 83.7%. Mp.: 138–139°C (EtOAc). ^1^H NMR (600 MHz, CDCl_3_) *δ* (ppm): 7.47–7.42 (m, 4H, H‐2”, 6”, 2”’, 6”’), 7.39–7.33 (m, 4H, H‐3”, 5”, 3”’, 5”’), 7.33–7.29 (m, 2H, H‐4”, 4”’), 7.15 (d, *J* = 2.1 Hz, 1H, H‐2), 6.98 (dd, *J* = 8.4, 2.1 Hz, 1H, H‐6), 6.93 (d, *J* = 8.4 Hz, 1H, H‐5), 5.18 (s, 2H, 3‐OC*H*_2_), 5.15 (s, 2H, 4‐OC*H*_2_), 3.91–3.86 (m, 1H, H‐2′), 1.87–1.78 (m, 3H, H‐3′, 6′), 1.72–1.62 (m, 2H, H‐4′, 6′), 1.59–1.48 (m, 2H, H‐4′, 5′), 1.41–1.31 (m, 1H, H‐5′). ^13^C NMR (151 MHz, CDCl_3_) *δ* (ppm): 148.94 (C‐3), 148.29 (C‐4), 139.85 (C‐1), 137.52 (C‐1”), 137.44 (C‐1”’), 128.63 (C‐3”, 5”), 128.60 (C‐3”’, 5”’), 128.00 (C‐4”), 127.93 (C‐4”’), 127.71 (C‐2”, 6”), 127.43 (C‐2”’, 6”’), 118.19 (C‐6), 115.09 (C‐5), 113.32 (C‐2), 75.64 (C‐1′), 74.55 (C‐2′), 71.71 (3‐O*C*H_2_), 71.48 (4‐O*C*H_2_), 38.66 (C‐6′), 29.34 (C‐3′), 24.44 (C‐4′), 21.26 (C‐5′).

#### 4.1.34 Synthesis of 4‐(1,2‐dihydroxycyclohexyl)benzene‐1,2‐diol (**25**)

Compound **25** was prepared in a procedure analogous to that used for the synthesis of derivative **16**, using compound **24** as the starting material. White solid. Yield: 93.4%. Mp.: 131–132°C (CH_2_Cl_2_). ^1^H NMR (600 MHz, MeOD) *δ* (ppm): 7.00 (d, *J* = 2.1 Hz, 1H, H‐2), 6.86 (dd, *J* = 8.3, 2.1 Hz, 1H, H‐6), 6.78 (d, *J* = 8.3 Hz, 1H, H‐5), 3.90–3.82 (m, 1H, H‐2′), 1.84–1.72 (m, 4H, H‐3′, 4′, 6′), 1.71–1.63 (m, 2H, H‐5′, 6′), 1.51–1.36 (m, 2H, Η‐ 4′, 5′). ^13^C NMR (151 MHz, MeOD) *δ* (ppm): 145.67 (C‐3), 144.60 (C‐4), 140.25 (C‐1), 117.75 (C‐6), 115.94 (C‐5), 114.02 (C‐2), 76.71 (C‐1′), 75.31 (C‐2′), 40.22 (C‐6′), 31.09 (C‐3′), 25.51 (C‐4′), 22.28 (C‐5′).

#### 4.1.35 Synthesis of 4‐(2‐Hydroxycyclohexyl)benzene‐1,2‐diol (**26**)

To a suspension of compound **26** (186 mg, 0.89 mmol) in CF_3_COOH (0.22 mL, 3 mmol), Et_3_SiH (0.32 mL, 2 mmol) was added dropwise, at 0°C and the resulting mixture was stirred at room temperature for 3 h. After completion of the reaction, the volatiles were vacuum evaporated and the residue was dissolved in CH_2_Cl_2_, washed with 10% NaHCO_3_, water, and brine, dried (anh. Na_2_SO_4_), and concentrated to dryness. Preparative high‐performance liquid chromatography (HPLC) system (H_2_O/MeOH 80/20 to H_2_O/MeOH 20/80) afforded 4‐[(1S,2 R)*‐2‐hydroxycyclohexyl]benzene‐1,2‐diol (**26a**) and 4‐[(1S,2S)*‐2‐hydroxycyclohexyl]benzne‐1,2‐diol (**26b**) in a ratio of 3/2, respectively.

4‐[(1*S*,2 *R*)*‐2‐hydroxycyclohexyl]benzene‐1,2‐diol (**26a**): White solid. Yield: 35.5%. Mp.: 160–161°C (EtOAc). ^1^H NMR (600 MHz, MeOD) *δ* (ppm): 6.68 (d, *J* = 8.1 Hz, 1H, Η‐5), 6.66 (d, *J* = 2.1 Hz, 1H, Η‐3, 6.54 (dd, *J* = 8.1, 2.1 Hz, 1, Η‐6), 3.50–3.55 (m, 1H, Η‐2′), 2.28–2.23 (m, 1H, Η‐1′), 2.11–2.02 (m, 1H, H‐3′), 1.84–1.78 (m, 2H, H‐4′, 6′), 1.76–1.71 (m, 1H, H‐5′), 1.50–1.41 (m, 2H, H‐4′, 6′), 1.39–1.29 (m, 2H, H‐3′, 5′). ^13^C NMR (151 MHz, MeOD) *δ* (ppm): 146.13 (C‐3), 144.38 (C‐4), 140.29 (C‐1), 119.89 (C‐6), 116.26 (C‐5), 115.66 (C‐2), 78.49 (C‐2′), 55.87 (C‐1′), 37.17 (C‐3′), 33.96 (C‐6′), 29.19 (C‐5′), 28.34 (C‐4′). (+) ESI QqToF (m/z): Calcd. for C_12_H_16_O_3_^+^: [M + H]+ 208.1099, found 208.1096.

4‐[(1*S*,2*S*)*‐2‐hydroxycyclohexyl]benzene‐1,2‐diol (**26b**): White solid. 12.6%. Mp.: 149–150°C (EtOAc). ^1^H NMR (600 MHz, MeOD) *δ* (ppm): 6.73 (d, *J* = 1.9 Hz, 1H, H‐2), 6.67 (d, *J* = 8.1 Hz, 1H, H‐5), 6.57 (dd, *J* = 8.1, 1.9 Hz, 1H, H‐6), 3.97–3.94 (m, 1H, H‐2′), 2.58–2.51 (m, 1H, H‐1′), 2.02–1.92 (m, 1H, H‐6′), 1.92–1.80 (m, 2H, H‐3′, 5′), 1.73–1.60 (m, 2H, H‐3′, 4′), 1.57 ‐ 1.45 (m, 2H, H‐4′, 6′), 1.45–1.37 (m, 1H, H‐5′). ^13^C NMR (151 MHz, MeOD) *δ* (ppm): 145.78 (C‐3), 144.21 (C‐4), 139.63 (C‐1), 120.33 (C‐6), 116.41 (C‐2), 115.97 (C‐5), 76.11 (C‐2′), 51.65 (C‐1′), 37.06 (C‐3′), 28.98 (C‐5′), 28.76 (C‐6′), 23.20 (C‐4′). (+) ESI QqToF (*m/z*): Calcd. for C_12_H_16_O_3_^+^: [M + H]+ 208.1099, found 208.1093.

#### 4.1.36 Synthesis of 4‐(cyclohept‐1‐en‐1‐yl)‐1,2‐(dibenzyloxy)benzene (**28**)

Derivative **28** was obtained after spontaneous dehydration of alcohol **27** which was prepared in a procedure analogous to that used for the synthesis of derivative **17**, using cycloheptanone as the starting material. White solid. Yield: 34%. Mp.: 59–60°C (EtOAc). ^1^H NMR (600 MHz, CDCl_3_) *δ* (ppm): 7.50 (d, *J* = 7.9 Hz, 2H, H‐2”,6”), 7.47 (d, *J* = 7.9 Hz, 2H, H‐2”’,6”’), 7.42–7.36 (m, 4H, H‐3”,5”,3”’,5”’), 7.36–7.31 (m, 2H, H‐4”,4”’), 6.98 (d, *J* = 2.0 Hz, 1H, H‐2), 6.90 (d, *J* = 8.3 Hz, 1H, H‐5), 6.88 (dd, *J* = 8.3, 2.0 Hz, 1H, H‐6), 6.02 (t, *J* = 6.8 Hz, 1H, H‐2′), 5.19 (s, 2H, 3‐OC*H*_2_), 5.18 (s, 2H, 4‐OC*H*_2_), 2.58–2.54 (m, 2H, H‐7′), 2.31–2.26 (m, 2H, H‐3′), 1.88–1.81 (m, 2H, H‐5′), 1.67–1.61 (m, 2H, H‐6′), 1.59–1.54 (m, 2H, H‐4′). ^13^C NMR (151 MHz, CDCl_3_) *δ* (ppm): 148.78 (C‐3), 147.95 (C‐4), 144.55 (C‐1′), 138.93 (C‐1), 137.65 (C‐1”,1”’), 129.42 (C‐2′), 128.59 (C‐3”,5”, 3”’,5”’), 127.90 (C‐4”), 127.86 (C‐4”’), 127.62 (C‐2”,6”), 127.46 (C‐2”’,6”’), 118.89 (C‐6), 115.10 (C‐5), 113.42 (C‐2), 71.70 (3‐O*C*H_2_), 71.64 (4‐O*C*H_2_), 32.88 (C‐5′,7′), 28.92 (C‐3′), 27.02 (C‐6′), 26.94 (C‐4′).

#### 4.1.37 Synthesis of 1‐[3,4‐(dibenzyloxy)phenyl]cycloheptane‐1,2‐diol (**29**)

Compound **29** was prepared in a procedure analogous to that used for the synthesis of derivative **24**, using compound **28** as the starting material. Pale yellow solid. Yield: 68.7%. Mp.: 118–119°C (EtOAc). ^1^H NMR (600 MHz, CDCl_3_) *δ* (ppm): 7.51–7.45 (m, 4H, H‐2”, 6”, 2”’, 6”’), 7.39 (td, *J* = 7.5, 2.2 Hz, 4H, H‐3”, 5”, 3”’, 5”’), 7.37–7.32 (m, 2H, H‐4”, 4”’), 7.18 (d, *J* = 2.1 Hz, 1H, H‐2), 7.01 (dd, *J* = 8.3, 2.1 Hz, 1H, H‐6), 6.95 (d, *J* = 8.3 Hz, 1H, H‐5), 5.21 (s, 2H, 3‐OC*H*_2_), 5.17 (s, 2H, 4‐OC*H*_2_), 3.90 (dd, *J* = 10.5, 2.4 Hz, 1H, H‐2′), 2.04–1.95 (m, 1H, H‐3′), 1.94–1.85 (m, 2H, H‐3′, 7′), 1.83–1.78 (m, 2H, H‐4′, 7′), 1.76–1.68 (m, 1H, H‐4′), 1.66–1.60 (m, 2H, H‐6′), 1.59– 1.53 (m, 1H, H‐5′), 1.51–1.44 (m, 1H, H‐5′). ^13^C NMR (151 MHz, CDCl_3_) *δ* (ppm): 148.78 (C‐3), 148.04 (C‐4), 141.69 (C‐1), 137.39 (C‐1”), 137.33 (C‐1”’), 128.49 (C‐3”, 5”), 128.47 (C‐3”’, 5”’), 127.86 (C‐4”), 127.80 (C‐4”’), 127.57 (C‐2”, 6”), 127.31 (C‐2”’, 6”’), 117.76 (C‐6), 114.91 (C‐5), 112.93 (C‐2), 78.71 (C‐1′), 77.30 (C‐2′), 71.60 (3‐O*C*H_2_), 71.35 (4‐O*C*H_2_), 39.24 (C‐7′), 29.64 (C‐3′), 26.76 (C‐5′), 22.88 (C‐4′), 20.09 (C‐6′).

#### 4.1.38 Synthesis of 2‐[3,4‐(dibenzyloxy)phenyl]cyclohept‐2‐en‐1‐ol (**30**)

Compound **30** was prepared in a procedure analogous to that used for the synthesis of derivative **23**, using compound **29** as the starting material. Oil. Yield: 67.7%. ^1^H NMR (600 MHz, CDCl_3_) *δ* (ppm): 7.54–7.47 (m, 4H, H‐2”, 6”, 2”’, 6”’), 7.43–7.38 (m, 4H, H‐3”, 5”, 3”’, 5”’), 7.37–7.33 (m, 2H, H‐4”, 4”’), 7.04 (d, *J* = 1.6 Hz, 1H, H‐2), 6.95–6.90 (m, 2H, H‐5, 6), 5.98 (t, *J* = 6.8 Hz, 1H, H‐7′), 5.21 (s, 2H, 3‐OC*H*_2_), 5.19 (s, 2H, 4‐OC*H*_2_), 4.76 (dd, *J* = 7.8, 1.9 Hz, 1H, H‐2′), 2.48–2.40 (m, 1H, H‐6′), 2.30–2.24 (m, 1H, H‐6′), 2.13–2.06 (m, 1H, H‐3′), 2.05–1.98 (m, 1H, H‐3′), 1.93–1.86 (m, 1H, H‐4′), 1.84–1.76 (m, 1H, H‐4′), 1.75–1.68 (m, 1H, H‐5′), 1.68–1.59 (m, 1H, H‐5′). ^13^C NMR (151 MHz, CDCl_3_) *δ* (ppm): 148.69 (C‐3), 148.22 (C‐4), 145.74 (C‐1′), 137.46 (C‐1”), 137.43 (C‐1”’), 136.56 (C‐1), 131.25 (C‐7′), 128.52 (C‐3”, 5”, 3”’, 5”’), 127.86 (C‐4”), 127.82 (C‐4”’), 127.57 (C‐2”, 6”), 127.37 (C‐2”’, 6”’), 120.00 (C‐6), 114.96 (C‐5), 114.42 (C‐2), 72.78 (C‐2′), 71.54 (3‐O*C*H_2_), 71.45 (4‐O*C*H_2_), 33.78 (C‐6′), 27.66 (C‐3′), 26.82 (C‐5′), 25.00 (C‐4′).

#### 4.1.39 Synthesis of 4‐(2‐hydroxycycloheptyl)benzene‐1,2‐diol (**31**)

Compounds **31a** and **31b** were prepared in a procedure analogous to that used for the synthesis of derivatives **21a** and **21b**, using compound **30** as the starting material.

4‐[(1*S*,2 *R*)*‐2‐hydroxycycleptyl]benzene‐1,2‐diol (**31a**): Oil. Yield: 58.3%. ^1^H NMR (600 MHz, MeOD) *δ* (ppm): 6.68 (d, *J* = 8.1 Hz, 1H, H‐5), 6.66 (d, *J* = 2.0 Hz, 1H, H‐2), 6.54 (dd, *J* = 8.1, 2.0 Hz, 1H, H‐6), 3.76–3.71 (m, 1H, H‐2′), 2.43–2.36 (m, 1H, H‐1′), 1.96–1.92 (m, 1H, H‐3′), 1.86–1.80 (m, 1H, H‐6′), 1.79–1.68 (m, 3H, H‐3′, 4′, 5′), 1.67–1.63 (m, 2H, H‐7′), 1.62 – 1.50 (m, 3H, H‐4′, 5′, 6′). ^13^C NMR (151 MHz, MeOD) *δ* (ppm): 146.13 (C‐3), 144.38 (C‐2), 140.29 (C‐1), 119.90 (C‐6), 116.26 (C‐5), 115.66 (C‐2), 78.49 (C‐2′), 55.87 (C‐1′), 37.17 (C‐3′), 33.95 (C‐7′), 29.19 (C‐5′), 28.34 (C‐6′), 23.01 (C‐4′). (+) ESI QqToF (*m/z*): Calcd. for C_13_H_18_O_3_^+^: [M + H]+ 222.1256, found 222.1255.

4‐[(1*S*,2*S*)*‐2‐hydroxycycloheptyl]benzene‐1,2‐diol (**31b**): Pale yellow solid. Yield: 20.6%. Mp.: 116–117°C (EtOAc). ^1^H NMR (600 MHz, MeOD) *δ* (ppm): 6.73 (d, *J* = 2.0 Hz, 1H, H‐2), 6.67 (d, *J* = 8.1 Hz, 1H, H‐5), 6.57 (dd, *J* = 8.1, 2.0 Hz, 1H, H‐6), 3.98–3.93 (m, 1H, H‐2′), 2.70–2.65 (m, 1H, H‐1′), 2.12–2.00 (m, 1H, H‐7′), 1.88–1.75 (m, 4H, H‐3′, 4′, 6′), 1.75–1.68 (m, 1H, H‐ 5′), 1.67–1.57 (m, 2H, H‐5′, 7′), 1.57–1.47 (m, 2H, H‐4′, 6′). ^13^C NMR (151 MHz, MeOD) *δ* (ppm): 145.79 (C‐3), 144.21 (C‐2), 139.64 (C‐1), 120.34 (C‐6), 116.42 (C‐2), 115.98 (C‐5), 75.12 (C‐2′), 51.65 (C‐1′), 37.06 (C‐3′), 29.42 (C‐5′), 28.98 (C‐7′), 28.76 (C‐6′), 23.20 (C‐4′). (+) ESI QqToF (*m/z*): Calcd. for C_13_H_18_O_3_^+^: [M + H]+ 222.1256, found 222.1256.

#### 4.1.40 Synthesis of 3,4‐dimethoxybenzophenone (**34a**)

A suspension of **32** (4.0 g, 21.98 mmol) in SOCl_2_ (10 mL, 137.7 mmol) was stirred at room temperature for 1 h. After completion of the reaction, the excess of SOCl_2_ was removed under reduced pressure. Without further purification, the corresponding chloride was dissolved in benzene (10 mL) and this solution was added dropwise to a suspension of AlCl_3_ (28 mmol, 3.72 g) in benzene (30 mL). The resulting mixture was stirred at room temperature for 3 h. The mixture was then poured into ice water, extracted with CH_2_Cl_2_ (3 × 40 mL), and the organic layer was dried (anh. Na_2_SO_4_) and evaporated to dryness. The residue was purified by column chromatography (silica gel), using a mixture of cyclohexane/CH_2_Cl_2_ 1/1 as the eluent, to afford 3.51 g of the compound **34a**.^[^ ^33^ ^]^ Oil. Yield: 87.75%. ^1^H NMR (600 MHz, CDCl_3_) *δ* (ppm): 7.79 (dd, *J* = 8.0, 2.0 Hz, 1H), 7.60 (td, *J* = 8.0, 2.0 Hz, 1H), 7.52 (d, *J* = 2.0 Hz, 1H), 7.50 (t, *J* = 8.0 Hz, 1H), 7.41 (dd, *J* = 8.0, 2.0 Hz, 1H), 6.92 (d, *J* = 8.0 Hz, 1H), 3.98 (s, 3H), 3.97 (s, 3H). ^13^C NMR (151 MHz, CDCl_3_) *δ* (ppm): 195.56, 153.05, 149.03, 138.30, 131.87, 130.24, 129.71, 128.17, 126.23, 125.49, 113.53, 112.16, 111.80, 109.77, 56.10, 56.06.

#### 4.1.41 Synthesis of bis(3,4‐dimethoxyphenyl)methanone (**34b**)

Compound **34b** was prepared in a procedure analogous to that used for the synthesis of derivative **34a**, using 1,2‐dimethoxybenzene as the starting material. Oil.^[^ ^34^ ^]^ Yield: 30%. ^1^H NMR (600 MHz, CDCl_3_) δ (ppm): 7.46 (d, *J* = 2.0 Hz, 1H), 7.41 (dd, *J* = 8.3, 2.0 Hz, 1H), 6.93 (d, *J* = 8.3 Hz, 1H), 3.99 (s, 3H), 3.96 (s, 3H). ^13^C NMR (151 MHz, CDCl_3_) δ (ppm): 194.60, 152.76, 149.04, 130.95, 124.89, 112.49, 109.91, 56.20.

#### 4.1.42 Synthesis of (3,4‐dimethoxyphenyl)phenylmethanol (**35a**)

To a solution of compound **34a** (214 mg, 1 mmol) in dry methanol (25 mL), NaBH_4_ (46 mg, 1.2 mmol) was added portion‐wise at 0°C. The resulting mixture was stirred at room temperature for 30 min. After completion of the reaction, the mixture was carefully acidified with 9% HCl, poured into water (60 mL), and washed with EtOAc (3 × 40 mL). The combined organic layers were dried over anhydrous Na_2_SO_4_ and evaporated to dryness. The residue was purified by recrystallization with EtOAc – *n*‐Pentane. White solid.^[^ ^35^ ^]^ Yield: 84%. Mp.: 121°C (EtOAc – *n*‐Pentane). ^1^H NMR (600 MHz, CDCl_3_) δ (ppm): 7.79 (dd, *J* = 8.0, 2.0 Hz, 1H), 7.60 (td, *J* = 8.0, 2.0 Hz, 1H), 7.52 (d, *J* = 2.0 Hz, 1H), 7.50 (t, *J* = 8.0 Hz, 1H), 7.41 (dd, *J* = 8.0, 2.0 Hz, 1H), 6.92 (d, *J* = 8.0 Hz, 1H), 5.83 (s, 1H), 3.98 (s, 3H), 3.97 (s, 3H). ^13^C NMR (151 MHz, CDCl_3_) δ (ppm): 149.06, 148.51, 143.90, 136.58, 129.74, 128.33, 127.53, 127.35, 127.08, 126.45, 119.91, 118.99, 80.00, 55.87.

#### 4.1.43 Synthesis of bis(3,4‐dimethoxyphenyl)methanol (**35b**)

Compound **35b** was prepared in a procedure analogous to that used for the synthesis of derivative **35a**, using **34b** as the starting material. Oil.^[^ ^36^ ^]^ Yield: 79%. ^1^H NMR (600 MHz, CDCl_3_) δ (ppm): 6.91 (d, *J* = 8.0, 2H), 6.85 (dd, *J* = 8.0, 2.0 Hz, 8H), 6.80 (d, *J* = 8.0, 2H), 5.70 (s, 1H), 3.84 (s, 6H), 3.82 (s, 6H), 1.42 (s, 1H). ^13^C NMR (151 MHz, CDCl_3_) δ (ppm): 148.96, 148.39, 134.95, 119.68, 110.88, 110.46, 79.93, 55.86

#### 4.1.44 Synthesis of 2‐(3,4‐dimethoxyphenyl)‐2‐phenylacetonitrile (**36a**)

A mixture of compound **35a** (2.38 g, 9.75 mmol), trimethylsilylcyanide (1.83 mL, 14.6 mmol), and BF_3_.Et_2_O (3.7 mL, 29 mmol) in anhydrous CH_2_Cl_2_ (10 mL) was stirred at room temperature for 40 h. The mixture was then poured into ice water, extracted with CH_2_Cl_2_ (3 × 40 mL), and the organic layer was dried (anh. Na_2_SO_4_) and evaporated to dryness, to afford 1.73 g of the title compound **36a**.^[^ ^37^ ^]^ Oil. Yield: 72%. ^1^H NMR (600 MHz, CDCl_3_) *δ* (ppm): 7.79 (dd, *J* = 8.0, 2.0 Hz, 1H), 7.60 (td, *J* = 8.0, 2.0 Hz, 1H), 7.52 (d, *J* = 2.0 Hz, 1H), 7.50 (t, *J* = 8.0 Hz, 1H), 7.41 (dd, *J* = 8.0, 2.0 Hz, 1H), 6.92 (d, *J* = 8.0 Hz, 1H), 5.12 (s, 1H), 3.98 (s, 3H), 3.97 (s, 3H). ^13^C NMR (151 MHz, CDCl_3_) *δ* (ppm): 149.52, 149.05, 136.09, 129.17, 128.44, 128.23, 127.63, 120.22, 119.86, 111.44, 110.81, 55.98, 42.17.

#### 4.1.45 Synthesis of 2,2‐bis(3,4‐dimethoxyphenyl)acetonitrile (**36b**)

Compound **36b** was prepared in a procedure analogous to that used for the synthesis of derivative **36a**, using **35b** as the starting material. Oil.^[^ ^38^ ^]^ Yield: 68%. ^1^H NMR (600 MHz, CDCl_3_) *δ* (ppm): 6.90 (d, *J* = 8.0, 2H), 6.86 (dd, *J* = 8.0 Hz, 2H), 6.82 (d, *J* = 8.0 Hz, 2H), 5.07 (s, 1H), 3.89 (s, 6H), 3.86 (s, 6H). ^13^C NMR (151 MHz, CDCl_3_) δ (ppm): 149.46, 148.99, 128.37, 120.09, 120.04, 111.38, 110.74, 55.98, 41.69.

#### 4.1.46 Synthesis of 2‐(3,4‐dimethoxyphenyl)‐2‐phenylacetic acid (**37a**)

A suspension of compound **36a** (320 mg, 1.26 mmol) in ethanol (10 mL) and 20% NaOH solution (10 mL) was stirred at 100°C for 10 h. After completion of the reaction, the volatiles were removed under reduced pressure and the aqueous solution was washed with ether (2 × 10 mL), acidified with 9% HCl (pH~2), and then extracted with CH_2_Cl_2_ (3 × 20 mL). The organic layer was dried over anhydrous Na_2_SO_4_ and evaporated to dryness to deliver 330 mg of compound **37a**. Oil. Yield: 96%. ^1^H NMR (600 MHz, CDCl_3_) *δ* (ppm): 10.82 (s, 1H, D_2_O exchange), 7.38 (dd, *J* = 8.0, 1H), 7.33 (td, *J* = 8.0, 2.0 Hz, 1H), 6.88 (d, *J* = 8.0 Hz, 1H), 5.08 (s, 1H), 3.90 (s, 3H), 3.87 (s, 3H). ^13^C NMR (151 MHz, CDCl_3_) *δ* (ppm): 178.80, 149.08, 148.55, 138.25, 130.43, 128.64, 128.46, 127.52, 121.10, 112.16, 111.27, 56.59, 55.93.

#### 4.1.47 Synthesis of 2,2‐bis(3,4‐dimethoxyphenyl)acetic acid (**37b**)

Compound **37b** was prepared in a procedure analogous to that used for the synthesis of derivative **37a**, using **36b** as the starting material. Oil.^[^ ^39^ ^]^ Yield: 85%. ^1^H NMR (600 MHz, CDCl_3_) *δ* (ppm): 6.91 (d, *J* = 8.0 Hz, 2H), 6.88 (dd, *J* = 8.0, 2.0 Hz, 2H), 6.85 (d, *J* = 8.0 Hz, 2H), 4.94 (s, 1H), 3.88 (s, 6H), 3.85 (s, 6H). ^13^C NMR (151 MHz, CDCl_3_) *δ* (ppm): 178.24, 148.94, 148.39, 130.48, 120.73, 111.85, 111.07, 65.77, 55.82.

#### 4.1.48 Synthesis of 2‐(3,4‐dimethoxyphenyl)‐2‐phenylethanol (**38a**)

Compound **38a** was prepared in a procedure analogous to that used for the synthesis of derivative **4a**, using **37a** as the starting material. Oil.^[^ ^40^ ^]^ Yield: 35%. ^1^H NMR (600 MHz, CDCl_3_) δ (ppm): 7.36–7.32 (m, 2H), 7.30–7.27 (m, 2H), 7.26–7.23 (m, 1H), 6.85 (d, *J* = 1.1 Hz, 2H), 6.80 (d, *J* = 1.2 Hz, 1H), 4.19–4.12 (m, 3H), 3.87 (s, 3H), 3.85 (s, 3H). ^13^C NMR (151 MHz, CDCl_3_) *δ* (ppm): 149.14, 147.93, 141.70, 134.00, 128.68, 128.20, 126.76, 120.12, 111.98, 111.41, 66.19, 55.92, 55.89, 53.20.

#### 4.1.49 Synthesis of 2,2‐bis(3,4‐dimethoxyphenyl)ethanol (**38b**)

Compound **38b** was prepared in a procedure analogous to that used for the synthesis of derivative **38a**, using **37b** as the starting material. Oil. Yield: 32%. ^1^H NMR (600 MHz, CDCl_3_) *δ* (ppm): 6.82 (s, 2H), 6.81 (dd, *J* = 8.0, 2.0 Hz, 2H), 6.77 (d, *J* = 8.0 Hz, 2H), 4.09 (d, *J* = 8.0 Hz, 2H), 3.84 (s, 12H), 3.82 (t, 1H). ^13^C NMR (151 MHz, CDCl_3_) *δ* (ppm): 149.08, 147.86, 134.19, 120.55, 119.97, 111.84, 111.33, 66.30, 55.88, 52.68.

#### 4.1.50 Synthesis of 2‐(3,4‐dihydroxyphenyl)‐2‐phenylethanol (**39a**)

Compound **39a** was prepared in a procedure analogous to that used for the synthesis of derivative **5a**, using **38a** as the starting material. Oil. Yield: 60.66%. ^1^H NMR (600 MHz, MeOD) *δ* (ppm): 7.28–7.23 (m, 4H), 7.18–7.13 (m, 1H), 6.71 (d, *J* = 8.1 Hz, 1H), 6.69 (d, *J* = 2.1 Hz, 1H), 6.60 (dd, *J* = 8.2, 2.1 Hz, 1H), 4.07–4.00 (m, 3H). ^13^C NMR (151 MHz, MeOD) *δ* (ppm): 146.14, 144.72, 144.19, 135.38, 129.27, 127.18, 120.60, 116.52, 116.26, 66.65, 54.40. (+) ESI QqToF (*m/z*): Calcd. for C_14_H_14_O_3_^+^: [M + H]+ 230.0943, found 230.0937.

#### 4.1.51 Synthesis of 2,2‐bis(3,4‐dihydroxyphenyl)ethanol (**39b**)

Compound **39b** was prepared in a procedure analogous to that used for the synthesis of derivative **8**, using **38b** as the starting material. Oil. Yield: 61%. ^1^H NMR (600 MHz, MeOD) *δ* (ppm): 6.72 (d, *J* = 8.0 Hz, 2H), 6.70 (d, *J* = 8.0 Hz, 2H), 6.61 (dd, *J* = 8.0, 2.0 Hz, 2H), 3.98 (d, *J* = 8.0 Hz, 2H), 3.87 (t, *J* = 7.5 Hz, 1H). ^13^C NMR (151 MHz, MeOD) *δ* (ppm): 146.11, 144.63, 135.99, 120.68, 116.46, 66.99, 61.68, 53.77. (+) ESI QqToF (*m/z*): Calcd. for C_14_H_14_O_5_^+^: [M + H]+ 262.0844, found 262.0849.

### 4.2 Pharmacological/biological assays

#### 4.2.1 Cell line, cell viability assay

MG63 cells, an osteoblast‐like human osteosarcoma cell line, are widely used for the evaluation of the biological activity of various compounds in the osteoblastic cellular microenvironment. MG‐63 cells were obtained from the American Type Culture Collection (ATCC). To evaluate the effect of the novel analogs on cell proliferation using a cell‐based protocol, an MTT assay was performed. Briefly, MG‐63 cells were cultured in RPMI‐1640 (Gibco) containing 10% fetal bovine serum (FBS, Gibco), at 37°C in 5% CO2. MG‐63 cells were plated at a density of 500 per well in a 96‐well plate and after 24 h, cells were treated with the compound in a dose‐dependent manner for 96 h. Dimethyl sulfoxide (DMSO) was used as vehicle control. MTT [3‐(4,5‐dimethylthiazol‐2‐yl)‐2,5‐diphenyltetrazolium bromide] (Sigma M‐5655) was added at a concentration of 5 mg/mL directly to each well for 4 h at 37°C. The medium was aspirated, and the blue MTT formazan precipitate was dissolved in DMSO. Absorbance was determined in a Powerwave microplate spectrophotometer (Biotek Instruments, Inc.) at 540 nm. Viable cell numbers were determined by tetrazolium conversion to its formazan dye. Each experiment was performed in triplicate mean values ± SD are reported.^[^ ^41^ ^]^

#### 4.2.2 Determination of DPPH radical‐scavenging activity

The DPPH assay was used to measure the free radical‐scavenging capacity of the molecules, according to a previously reported method,^[^ ^42^ ^]^ with modifications. The appropriate amount of each molecule, diluted in ethanol, was mixed with 35 μL of a freshly prepared ethanolic solution of 0.4 mg/mL DPPH in microplate wells. The concentrations used were in the range of 1–75 μΜ and the total volume of the assay was 0.2 mL. The solutions were incubated at 37°C for 30 and the absorbance was measured at 492 nm using a microplate reader. The results were expressed as the IC_50_ values (μΜ in the reaction mixture), giving the molecule concentration needed to achieve 50% scavenging of DPPH radical.

#### 4.2.3 Soybean lipoxygenase inhibition assay

The assay was performed according to a previously described procedure,^[^ ^43^ ^]^ with some modifications. The incubation mixture consisted of the appropriate amount of each molecule diluted in ethanol (and add up to 40 μL water), 5 μL of the enzyme solution (60 units/assay in boric acid buffer), and 165 μL of 0.2 M boric acid buffer, pH 9.0. After incubation at room temperature for 5 min in the dark, the reaction was started by adding 40 μL of linoleic acid solution (937 μM in 25 μL dimethyl sulfoxide and 1225 μL buffer). The total volume of the reaction solution was 250 μL and the final concentration of linoleic acid was 150 μM in the reaction mixture. The conversion of linoleic acid to 13‐hydroperoxylinoleic acid was recorded at 234 nm (room temperature) and compared with the appropriate standard solution, which did not contain the molecules. Every sample was tested at least five times at 200 μM concentration and the percentage of inhibition was calculated.

#### 4.2.4 Statistical analysis

Normality was tested with the Kolmogorov–Smirnoff criterion. All the variables had normal distribution and the results were expressed as mean values ± SEM. For the comparison of the molecules, one‐way analysis of variance (ANOVA) was performed and a post hoc analysis was carried out, where appropriate, with the Bonferroni correction. The level of significance used was 5%. SPSS v18 software was used for statistical analysis.

## CONFLICTS OF INTEREST STATEMENT

The authors declare no conflicts of interest.

## Supporting information

Supporting information.

Supporting information.

## Data Availability

The data that support the findings of this study are available on request from the corresponding author. The data are not publicly available due to privacy or ethical restrictions.
