# Supplementary material for: Synthesis of hydroxytyrosol analogs with enhanced antioxidant and cytostatic properties against MG‐63 human osteoblast‐like cells and their potential implications for bone health
Source: Arch Pharm (Weinheim). 2024 Nov 16;358(1):e2400469. doi: 10.1002/ardp.202400469 (PMC11726141; doi:10.1002/ardp.202400469)
Supplement: Supplementary file 1 — Supporting information. [file ARDP-358-e2400469-s002.doc]

**Supplemental Material: Novel Compounds and Biological Screening Results**

Synthesis of Hydroxytyrosol Analogues with Enhanced Antioxidant and Cytostatic Properties Against MG-63 Human Osteoblast-Like Cells and Their Potential Implications for Bone Health.

Georgiou A. Eleftheria^1^, Kalpaktsi Ioanna^1^, Gioti Katerina^2^, Choleva Maria^2^, Fragopoulou Elizabeth^2^, Alexios-Leandros Skaltsounis^3^, Tenta Roxane^2^, Kostakis K. Ioannis^1,*^

1 Division of Pharmaceutical Chemistry, Department of Pharmacy, National and Kapodistrian University of Athens, Panepistimiopolis Zografou 15771, Athens, Greece

2 Department of Nutrition & Dietetics, School of Health Sciences and Education, Harokopio University, Athens, Greece

3 Division of Pharmacognosy and Natural Products Chemistry, Department of Pharmacy, National and Kapodistrian University of Athens, Panepistimiopolis Zografou 15771, Athens, Greece

*Correspondence:

Dr. Kostakis K. Ioannis, Division of Pharmaceutical Chemistry, Department of Pharmacy, National and Kapodistrian University of Athens, Panepistimiopolis Zografou 15771, Athens, Greece

Email: ikkostakis@pharm.uoa.gr

| **Compound No.** | **InChI** | human MG-63 cells IC_50_ (μΜ) | Scavenging of DPPH radical EC_50_ (μΜ) | % Inhibition of lipoxygenase |
| --- | --- | --- | --- | --- |
| 1 | InChI=1S/C9H12O3/c1-6(5-10)7-2-3-8(11)9(12)4-7/h2-4,6,10-12H,5H2,1H3 | 18.4 | NT | NT |
| 2 | InChI=1S/C10H14O3/c1-10(2,6-11)7-3-4-8(12)9(13)5-7/h3-5,11-13H,6H2,1-2H3 | 15 | NT | NT |
| 3 | InChI=1S/C12H16O3/c13-8-12(5-1-2-6-12)9-3-4-10(14)11(15)7-9/h3-4,7,13-15H,1-2,5-6,8H2 | 8 | 32.9±4.8 | 19.3±3 |
| 4 | InChI=1S/C11H14O3/c12-7-11(4-1-5-11)8-2-3-9(13)10(14)6-8/h2-3,6,12-14H,1,4-5,7H2 | 10.5 ± 1.8 | 32.7±8.6 | 23.3±10.1 |
| 5 | InChI=1S/C13H18O3/c14-9-13(6-2-1-3-7-13)10-4-5-11(15)12(16)8-10/h4-5,8,14-16H,1-3,6-7,9H2 | 4.3 ± 0.4 | 28.7±7.7 | 41.4±10.4 |
| 6 | InChI=1S/C14H20O3/c15-10-14(7-3-1-2-4-8-14)11-5-6-12(16)13(17)9-11/h5-6,9,15-17H,1-4,7-8,10H2 | 7.8 ± 0.5 | 29.3±5.3 | 31.0±10.3 |
| 7 | InChI=1S/C11H14O3/c12-9-3-1-2-8(9)7-4-5-10(13)11(14)6-7/h4-6,8-9,12-14H,1-3H2/t8-,9+/m0/s1 | 30.1 ± 1.2 | 34.3±8.2 | 26.4±3.2 |
| 8 | OC1=CC=C([C@H]2[C@@H](O)CCC2)C=C1O | 20.9 ± 1.5 | 32.2±11.3 | 38,6±6.5 |
| 9 | InChI=1S/C12H16O4/c13-9-5-4-8(7-10(9)14)12(16)6-2-1-3-11(12)15/h4-5,7,11,13-16H,1-3,6H2 | > 40 | 27.7±8.2 | 26.0±9.8 |
| 10 | InChI=1S/C12H16O3/c13-10-4-2-1-3-9(10)8-5-6-11(14)12(15)7-8/h5-7,9-10,13-15H,1-4H2/t9-,10+/m0/s1 | 10.8 ± 1.1 | 30.1±7.8 | 17.4±4.1 |
| 11 | InChI=1S/C12H16O3/c13-10-4-2-1-3-9(10)8-5-6-11(14)12(15)7-8/h5-7,9-10,13-15H,1-4H2/t9-,10-/m0/s1 | 30 ± 2.8 | 41.2±9.3 | 11.4±6.0 |
| 12 | InChI=1S/C13H18O3/c14-11-5-3-1-2-4-10(11)9-6-7-12(15)13(16)8-9/h6-8,10-11,14-16H,1-5H2/t10-,11+/m0/s1 | 28.3 ± 2.3 | 36.4±5.8 | 15.2±3.6 |
| 13 | InChI=1S/C13H18O3/c14-11-5-3-1-2-4-10(11)9-6-7-12(15)13(16)8-9/h6-8,10-11,14-16H,1-5H2/t10-,11-/m0/s1 | 24.8 ± 0.2 | 31.5±5.4 | 25.2±9.8 |
| 14 | InChI=1S/C14H14O3/c15-9-12(10-4-2-1-3-5-10)11-6-7-13(16)14(17)8-11/h1-8,12,15-17H,9H2 | 25.4 ± 1 | 30.5±7.2 | 22.6±5.9 |
| 15 | InChI=1S/C14H14O5/c15-7-10(8-1-3-11(16)13(18)5-8)9-2-4-12(17)14(19)6-9/h1-6,10,15-19H,7H2 | 6.4 ± 0.3 | 24.8±3.7 | 27.7±12.9 |

Cell line - Cell viability assay

MG63 cells, an osteoblast - like human osteosarcoma cell line, are widely used for the evaluation of the biological activity of various compounds on the osteoblastic cellular microenvironment. MG-63 cells were obtained from the American Type Culture Collection (ATCC, Bethesda, MD). In order to evaluate the effect of the novel analogues on cell proliferation using a cell-based protocol, MTT assay was performed. Briefly, MG-63 cells were cultured in RPMI-1640 (Gibco, USA) containing 10% fetal bovine serum (FBS, Gibco, USA), at 37°C in 5% CO2. MG-63 cells were plated at a density of 500 per well in a 96-well plate and after 24 h, cells were treated with the compound in a dose-dependent manner for 96h. Dimethyl sulfoxide (DMSO) was used as vehicle control. MTT [3-(4,5-dimethylthiazol-2-yl)-2,5-diphenyltetrazolium bromide] (Sigma M-5655) was added at a concentration of 5 mg/ml directly to each well for 4 h at 37oC. The medium was aspirated, and the blue MTT formazan precipitate was dissolved in DMSO. Absorbance was determined in a Powerwave microplate spectrophotometer (Biotek Instruments, Inc., Vermont, USA) at 540 nm. Viable cell numbers were determined by tetrazolium conversion to its formazan dye. Each experiment was performed in triplicate mean values ± SD are reported.

Determination of DPPH Radical-Scavenging Activity

The DPPH assay was used to measure the free radical-scavenging capacity of the molecules, according to a previously reported method [?], with modifications. The appropriate amount of each molecule, diluted in ethanol, was mixed with 35 μL of a freshly prepared ethanolic solution of 0.4 mg/mL DPPH in microplate wells. The concentrations used were in the range of 1 το 75 μΜ and the total volume of the assay was 0.2 mL. The solutions were incubated at 37 °C for 30 and the absorbance were measured at 492 nm with a microplate reader. The results were expressed as the IC_50_ values (μΜ in the reaction mixture), giving the molecule concentration needed to achieve 50% scavenging of DPPH radical.

Soybean Lipoxygenase Inhibition Assay

The assay was performed according to a previously described procedure [?], with some modifications. The incubation mixture consisted of the appropriate amount of each molecule diluted in ethanol (and add up to 40 μL water), 5 μL of the enzyme solution (60 units/assay in boric acid buffer) and 165 μL of 0.2 M boric acid buffer, pH 9.0. After incubation at room temperature for 5 min in the dark, the reaction was started by adding 40 μL of linoleic acid solution (937 μM in 25 μL dimethyl sulfoxide and 1225 μL buffer). The total volume of the reaction solution was 250 μL and the final concentration of linoleic acid was 150 μM in the reaction mixture. The conversion of linoleic acid to 13-hydroperoxylinoleic acid was recorded at 234 nm (room temperature) and compared to the appropriate standard solution, which did not contain the molecules. Every sample was tested at least five times at 200 μM concentration and the percentage of inhibition was calculated.
