# Supplementary material for: Synthesis of hydroxytyrosol analogs with enhanced antioxidant and cytostatic properties against MG‐63 human osteoblast‐like cells and their potential implications for bone health
Source: Arch Pharm (Weinheim). 2024 Nov 16;358(1):e2400469. doi: 10.1002/ardp.202400469 (PMC11726141; doi:10.1002/ardp.202400469)
Supplement: Supplementary file 2 — Supporting information. [file ARDP-358-e2400469-s001.pdf]

# Supplementary Material

# Synthesis and Characterization of Hydroxytyrosol Derivatives with Enhanced Biological Activity

Georgiou A. Eleftheria[1], Kalpaktsi Ioanna [1], Gioti Katerina[2], Alexios-Leandros Skaltsounis[3],Tenta Roxane[2], Kostakis K. Ioannis[1,*]

1 Division of Pharmaceutical Chemistry, Department of Pharmacy, National and Kapodistrian University of Athens, Panepistimiopolis Zografou 15771, Athens, Greece
2 Department of Nutrition & Dietetics, School of Health Sciences and Education, Harokopio University, Athens, Greece
3 Division of Pharmacognosy and Natural Products Chemistry, Department of Pharmacy, National and Kapodistrian University of Athens, Panepistimiopolis Zografou 15771, Athens, Greece

*Correspondence:
Dr. Kostakis K. Ioannis, Division of Pharmaceutical Chemistry, Department of Pharmacy, National and Kapodistrian University of Athens, Panepistimiopolis Zografou 15771, Athens, Greece
Email: ikkostakis@pharm.uoa.gr

# Contents


Figure S1: $^{1}$H NMR of compound **5b** 3

Figure S2: $C^{13}$ NMR of compound **5b** 4

Figure S3: $^{1}$H NMR of compound **8** 4

Figure S4: $C^{13}$ NMR of compound **8** 5

Figure S5: $^{1}$H NMR of compound **16b** 5

Figure S6: $C^{13}$ NMR of compound **16b** 6

Figure S7: $^{1}$H NMR of compound **16c** 6

Figure S8: $C^{13}$ NMR of compound **16c** 7

Figure S9: $^{1}$H NMR of compound **21a** 7

Figure S10: $C^{13}$ NMR of compound **21a** 8

Figure S11: $^{1}$H NMR of compound **21b** 8

Figure S12: $C^{13}$ NMR of compound **21b** 9

Figure S13: $^{1}$H NMR of compound **25** 9

Figure S14: $C^{13}$ NMR of compound **25** 10

Figure S15: $^{1}$H NMR of compound **26a** 10

Figure S16: $C^{13}$ NMR of compound **26a** 11

Figure S17: $^{1}$H NMR of compound **26b** 11

Figure S18: $C^{13}$ NMR of compound **26b** 12

Figure S19: $^{1}$H NMR of compound **39a** 12

Figure S20: $C^{13}$ NMR of compound **39a** 12

Figure S21: $^{1}$H NMR of compound **39b** 13

Figure S22: $C^{13}$ NMR of compound **39b** 13

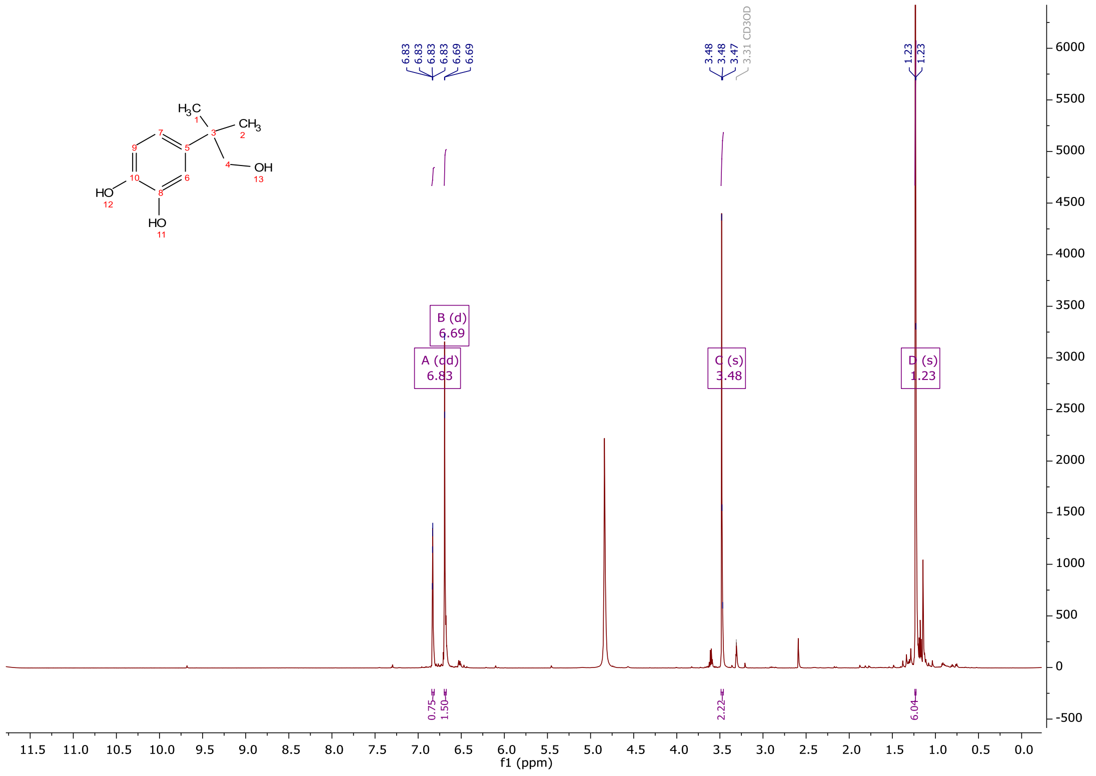


Figure S1: $^{1}$H NMR of compound **5b** in MeOD

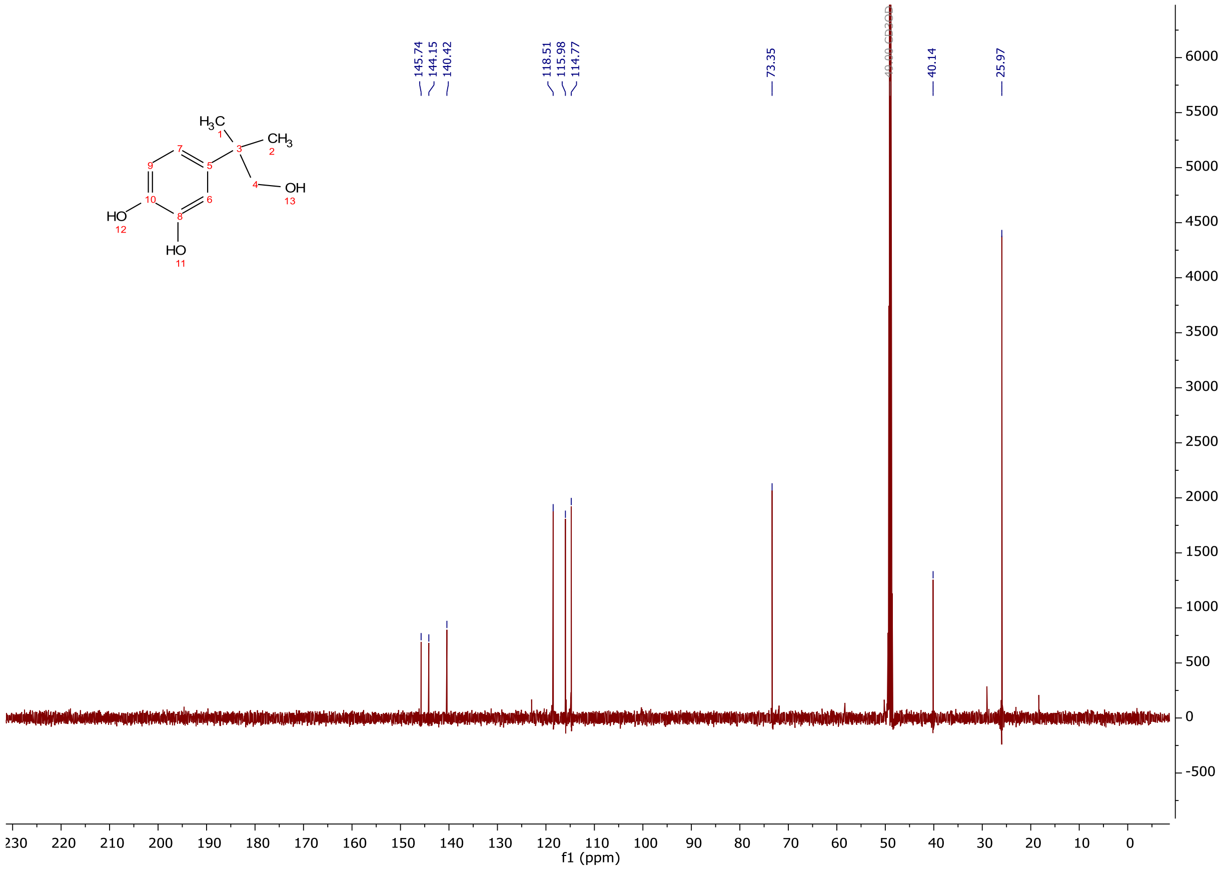

Figure S2: C[13] NMR of compound **5b** in MeOD

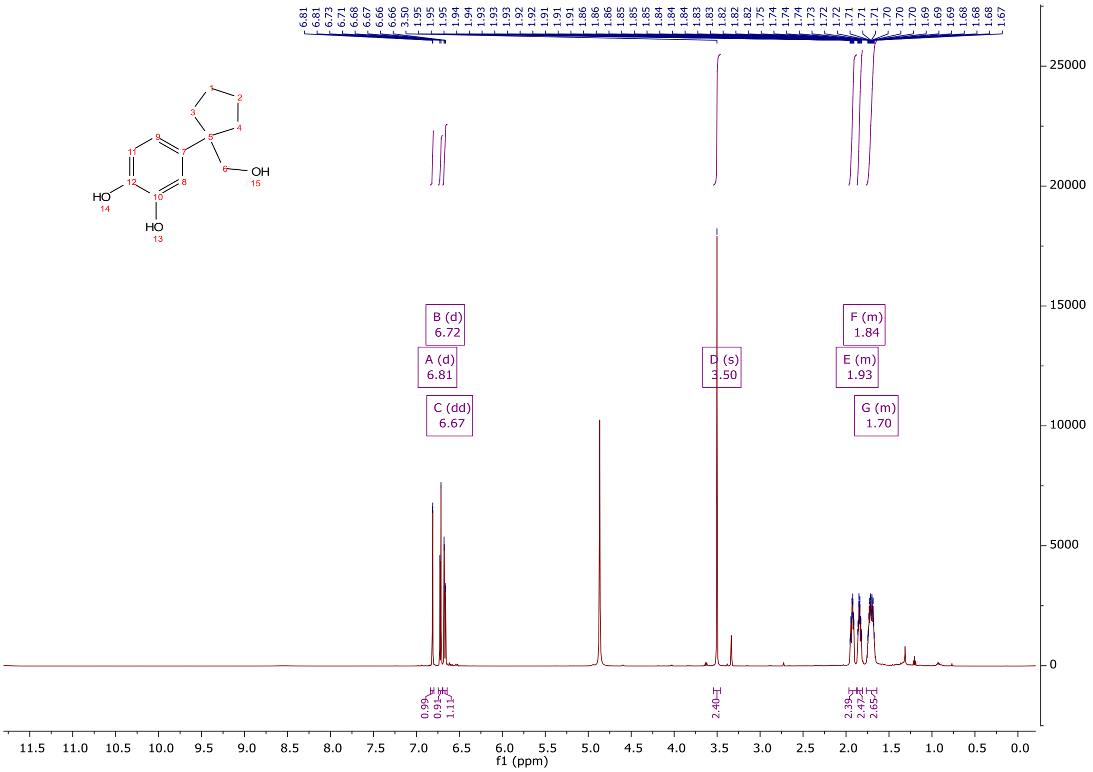


Figure S3: $^{1}$H NMR of compound **8** in MeOD

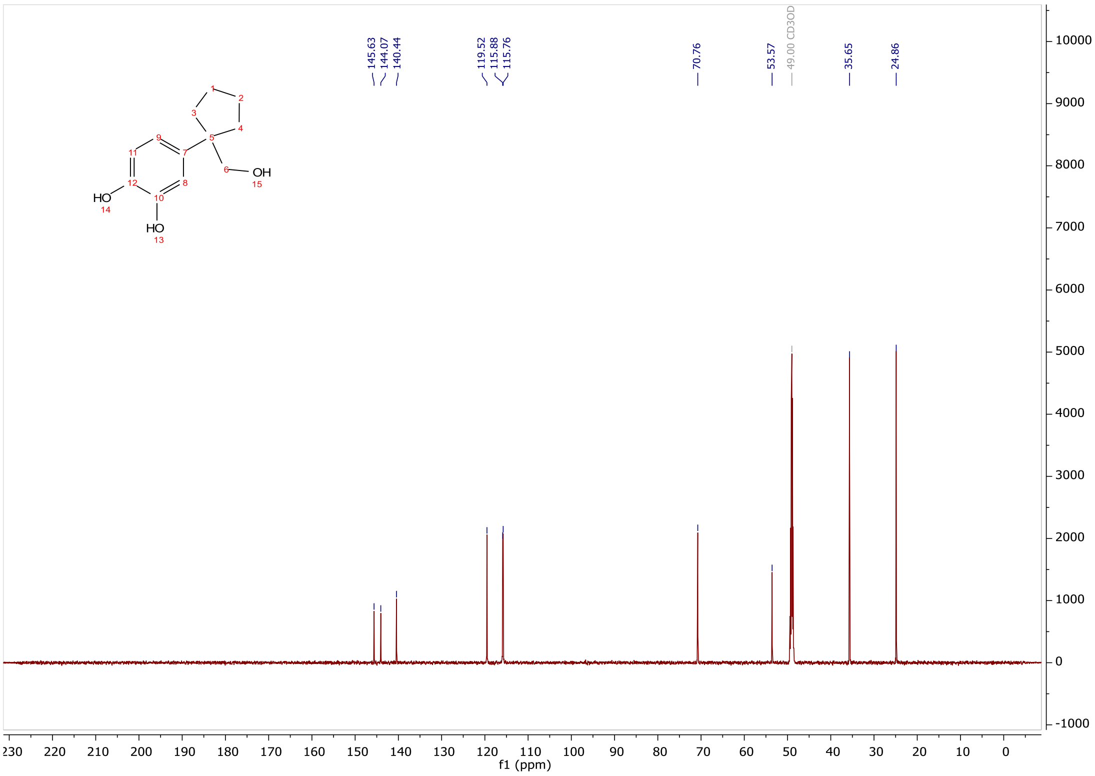

Figure S4: C[13] NMR of compound **8** in MeOD

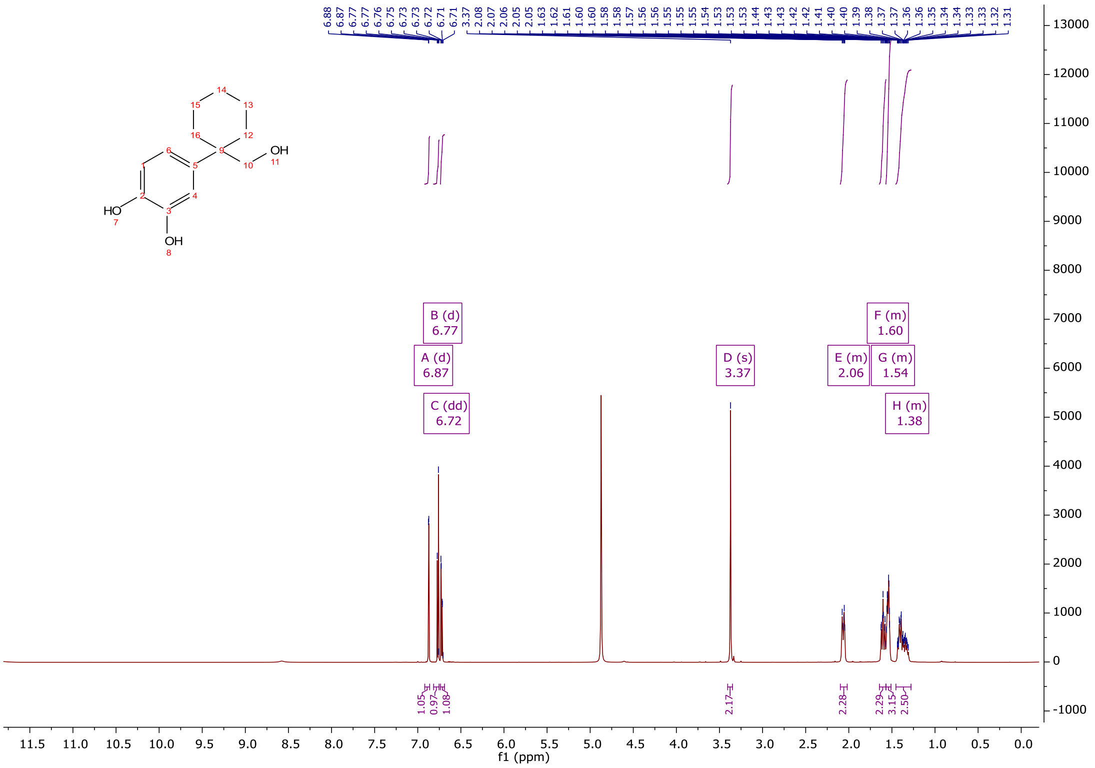

Figure S5: $^{1}$H NMR of compound **16b** in MeOD

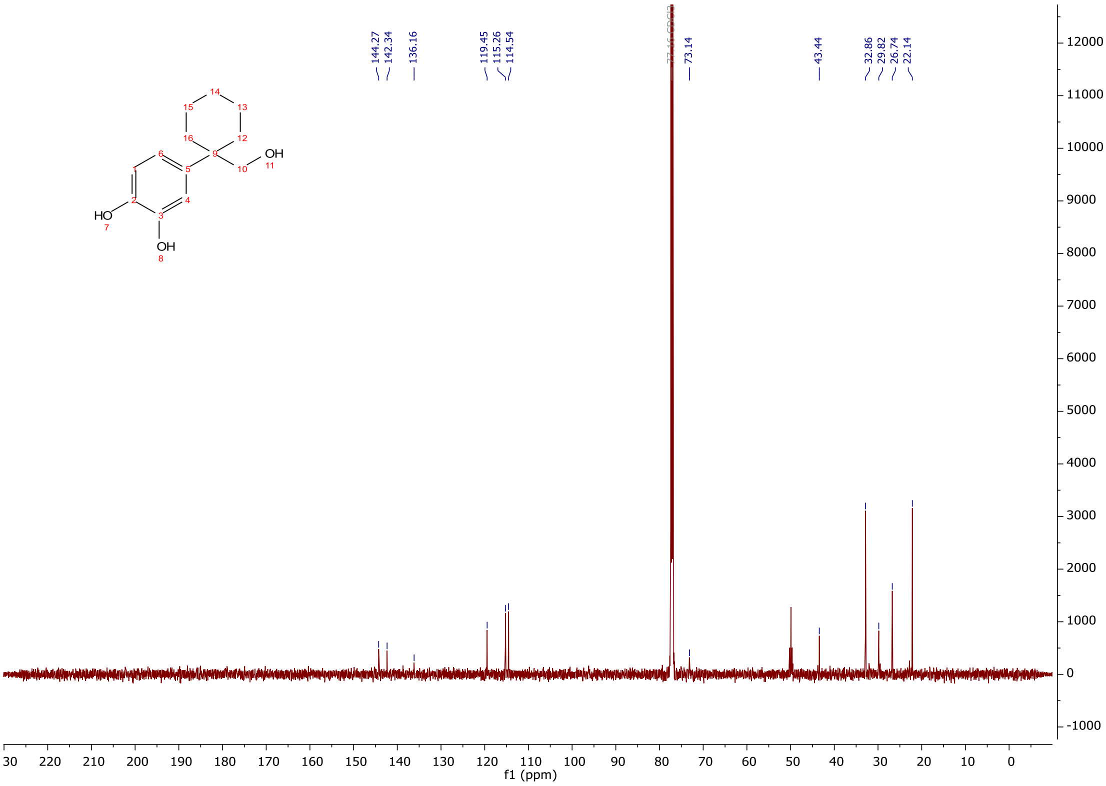

## Figure S6: C$^{13}$ NMR of compound **16b** in MeOD

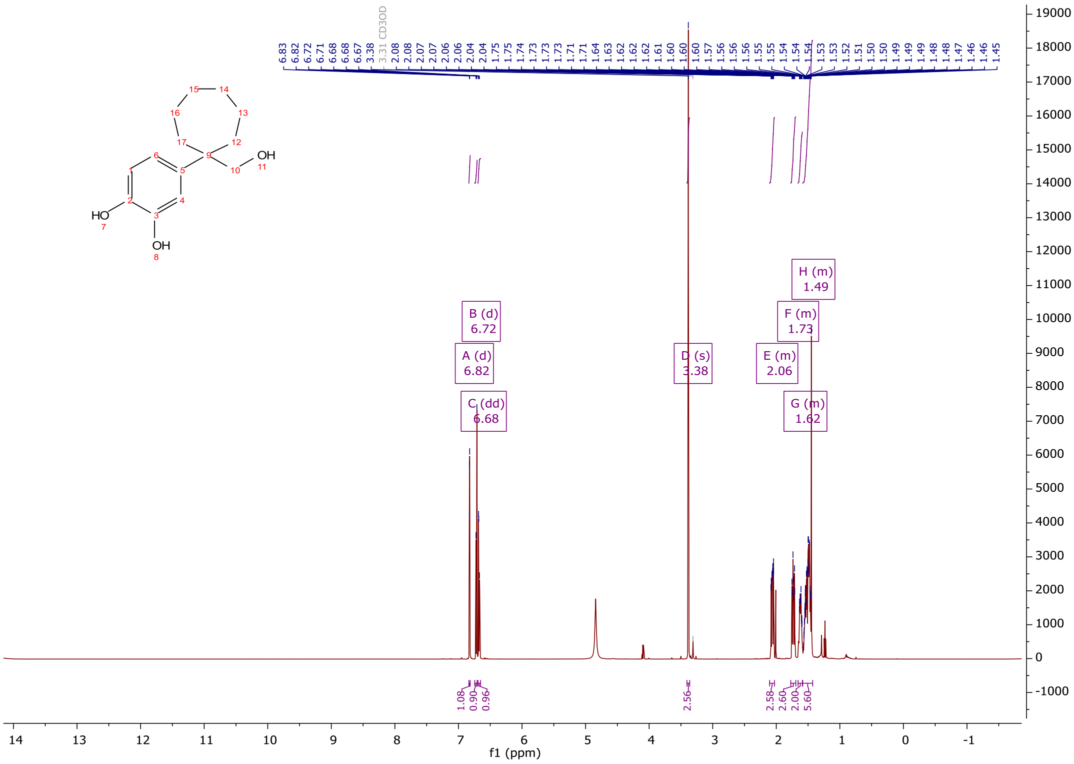


## Figure S7: $^{1}$H NMR of compound **16c** in MeOD

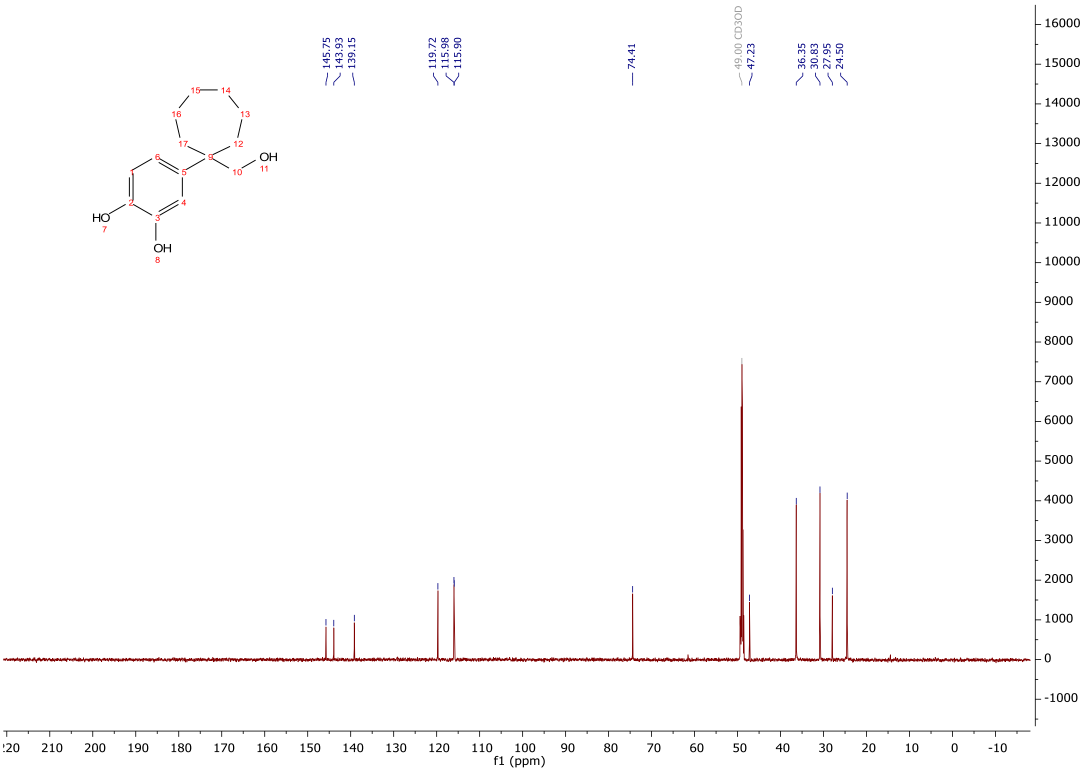

Figure S8: C[13] NMR of compound **16c** in MeOD

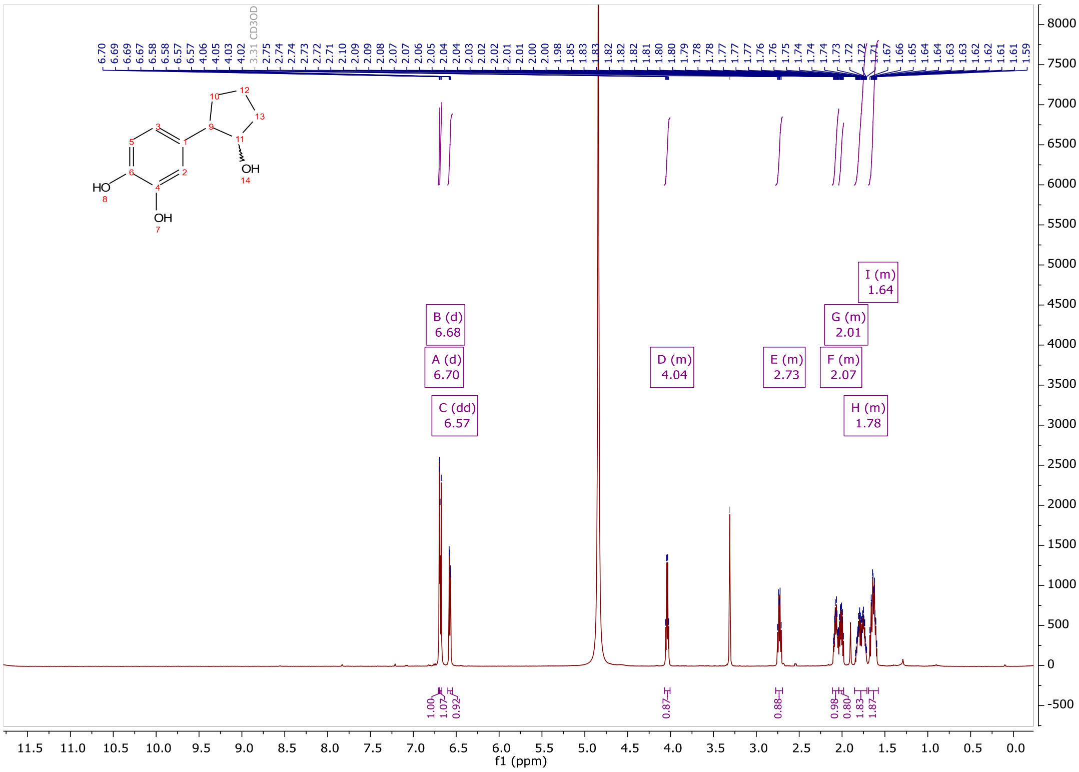


Figure S9: $^{1}$H NMR of compound **21a** in MeOD

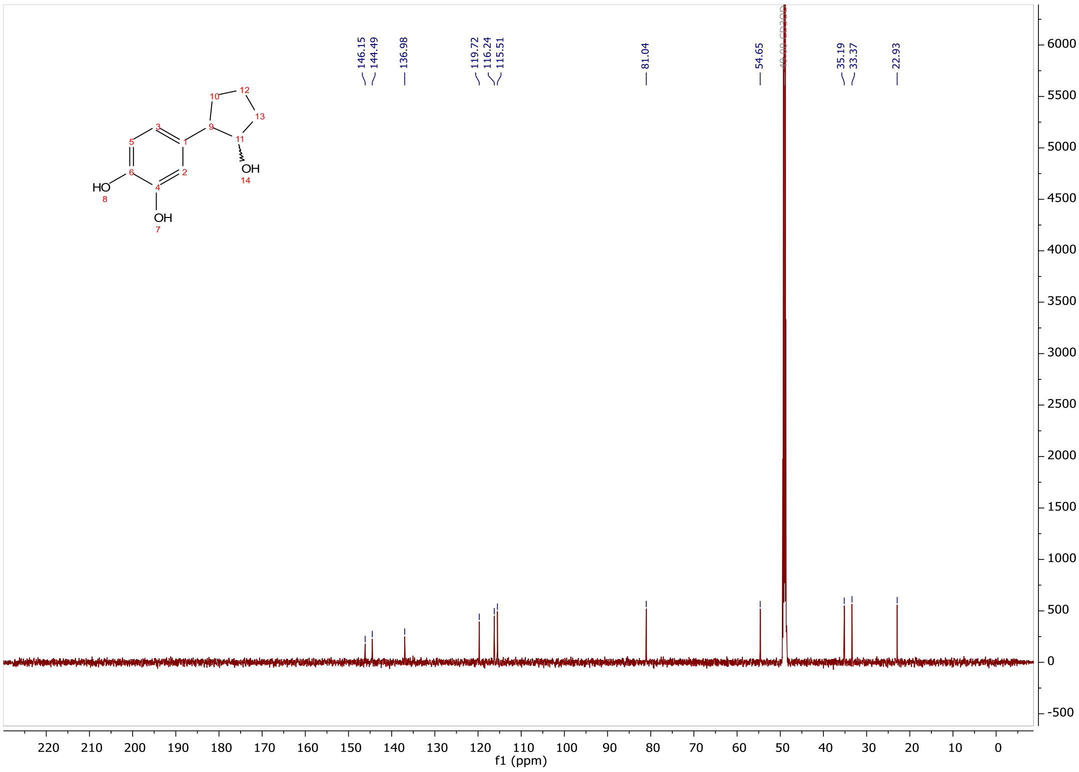

Figure S10: C[13] NMR of compound **21a** in MeOD

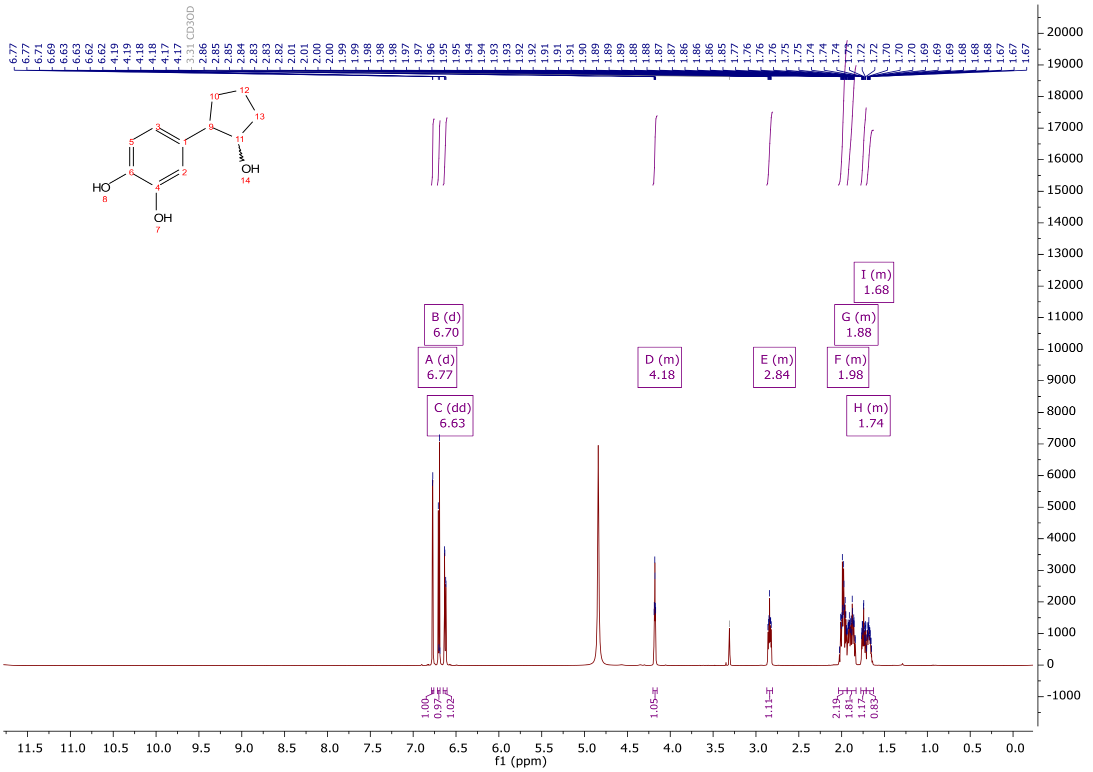


Figure S11: $^{1}$H NMR of compound **21b** in MeOD

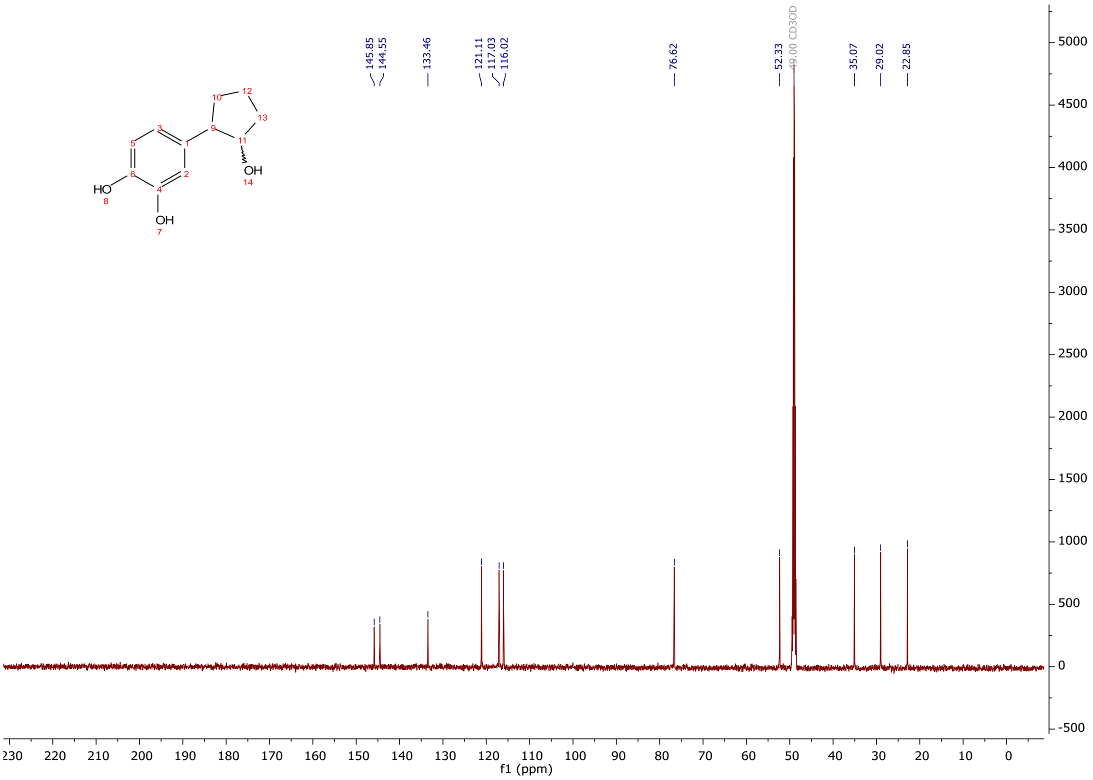

Figure S12: C[13] NMR of compound **21b** in MeOD

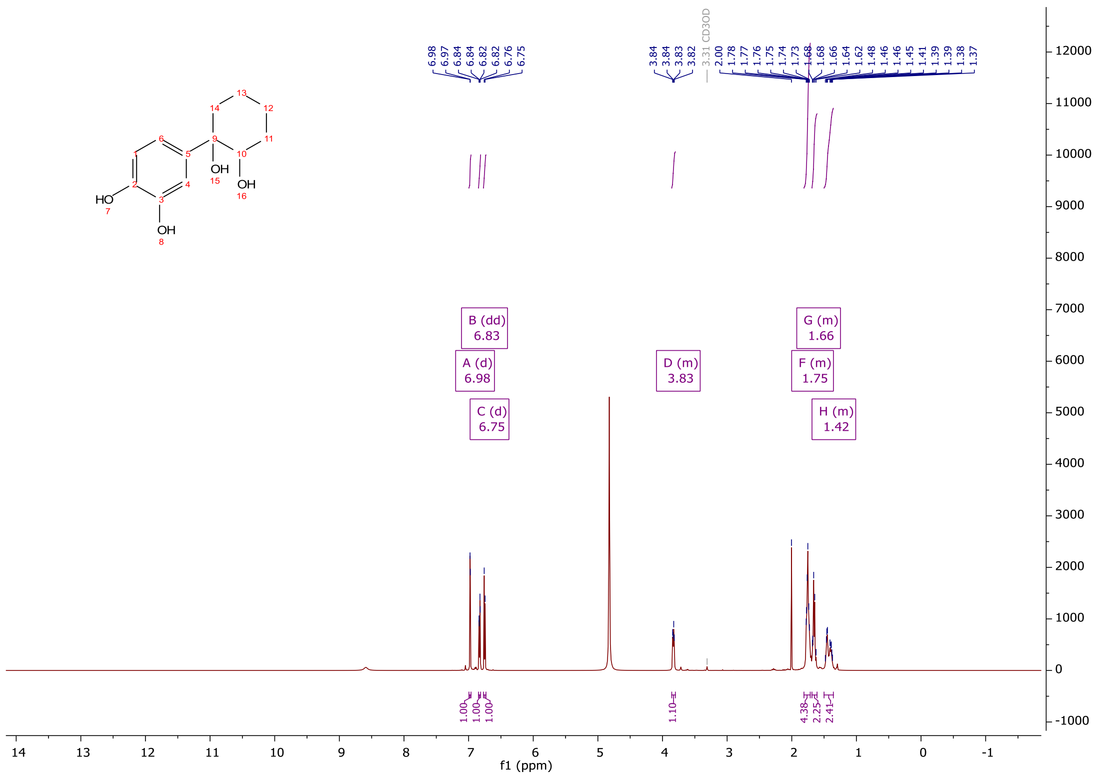


Figure S13: $^{1}$H NMR of compound **25** in MeOD

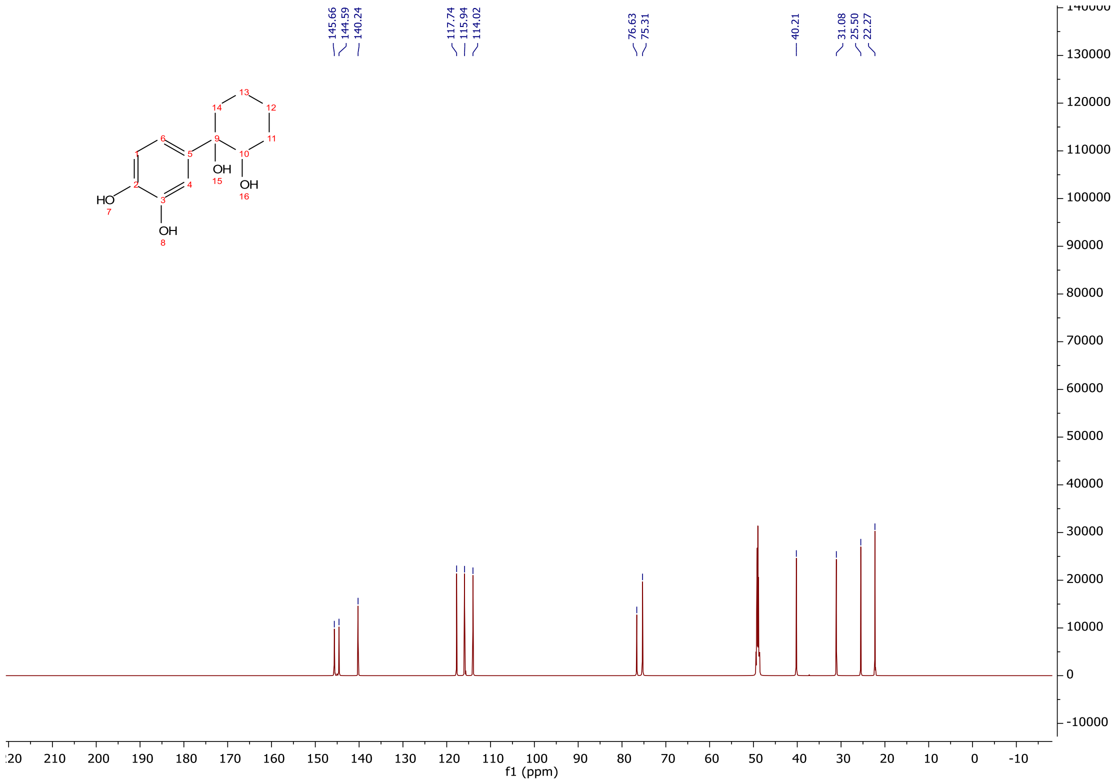

Figure S14: C[13] NMR of compound **25** in MeOD

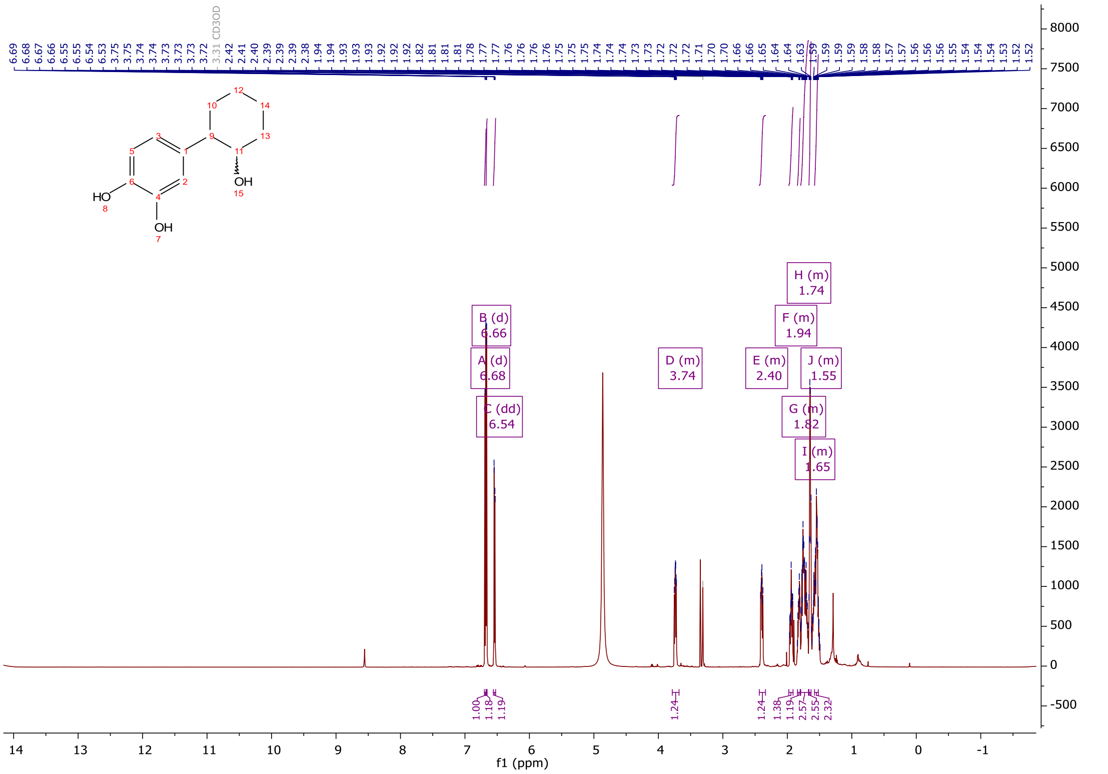

Figure S15: $^{1}$H NMR of compound **26a** in MeOD

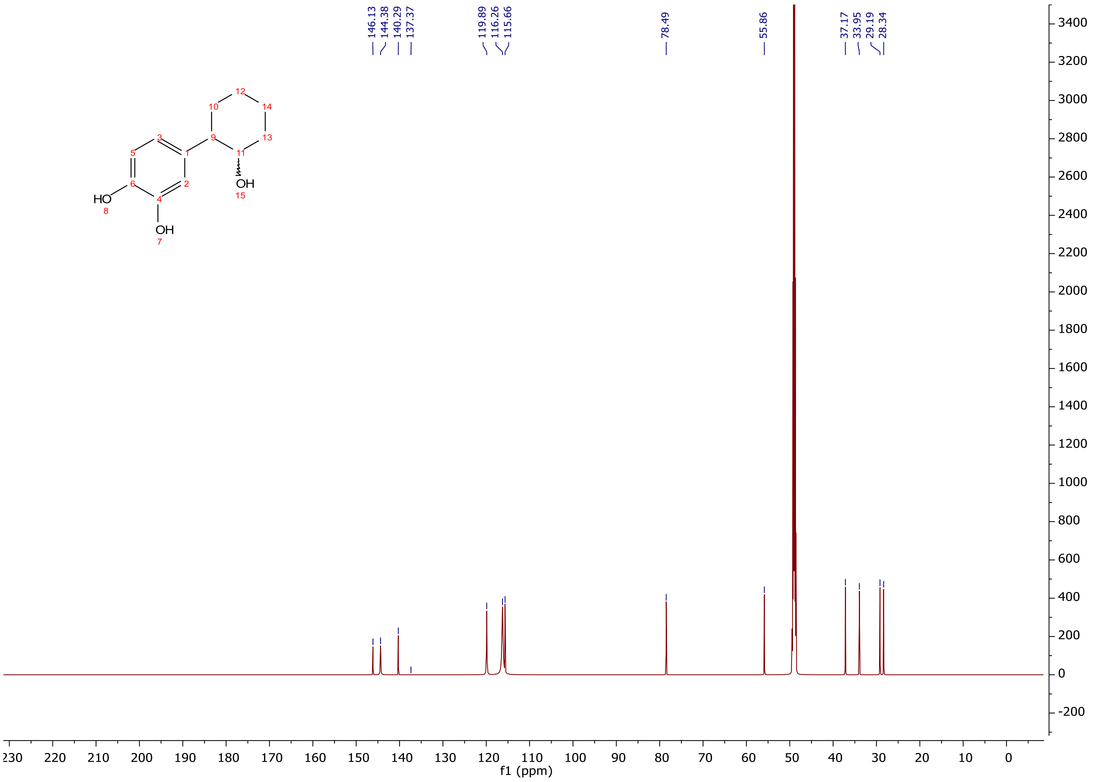

Figure S16: C[13] NMR of compound **26a** in MeOD

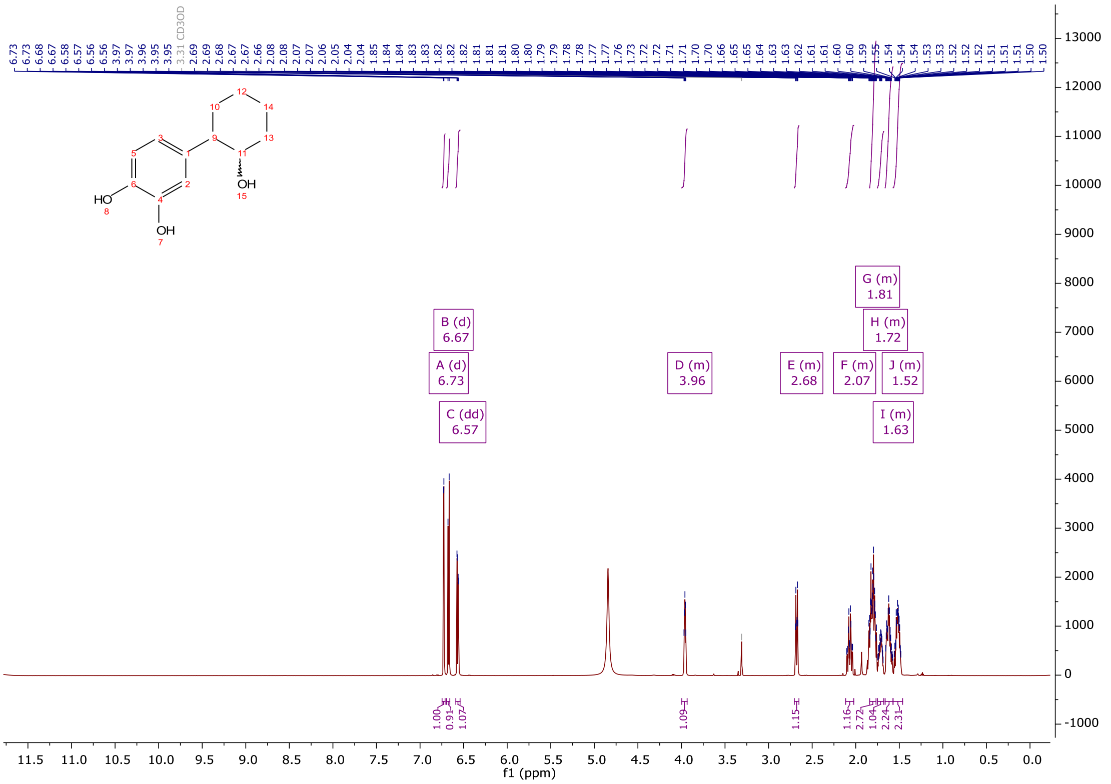


Figure S17: $^{1}$H NMR of compound **26b** in MeOD

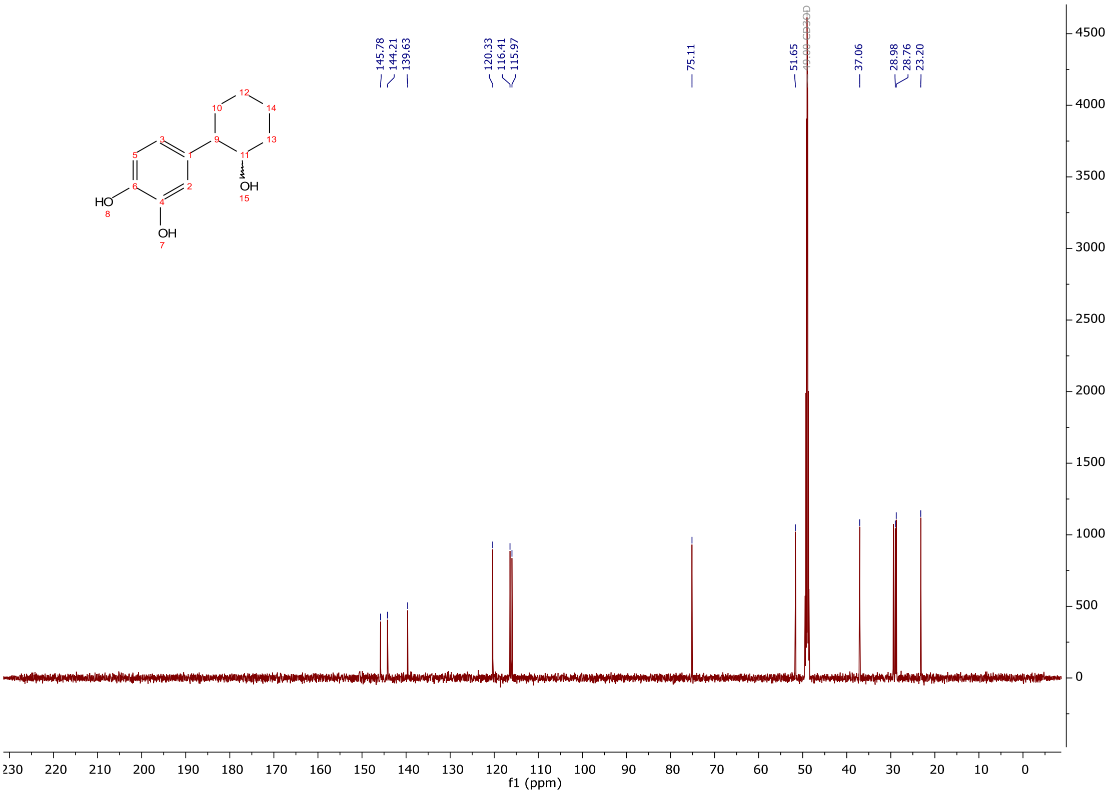

Figure S18: C[13] NMR of compound **26b** in MeOD

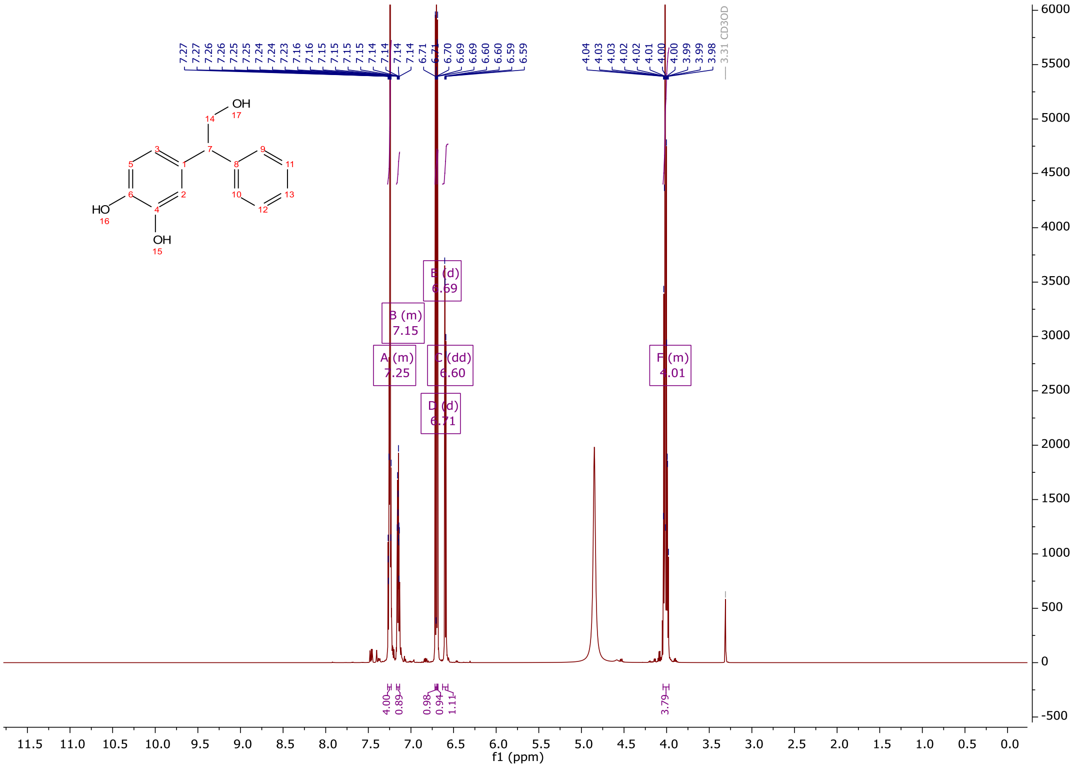


Figure S19: $^{1}$H NMR of compound **39a** in MeOD

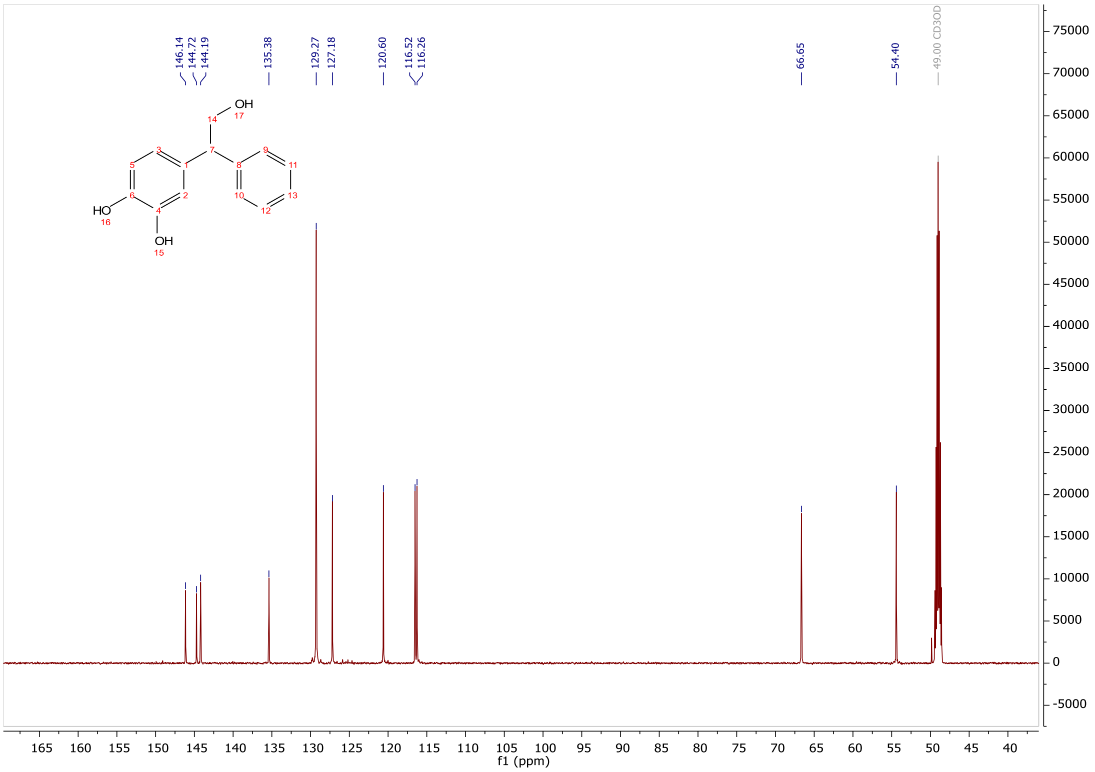


Figure S20: C[13] NMR of compound **39a** in MeOD

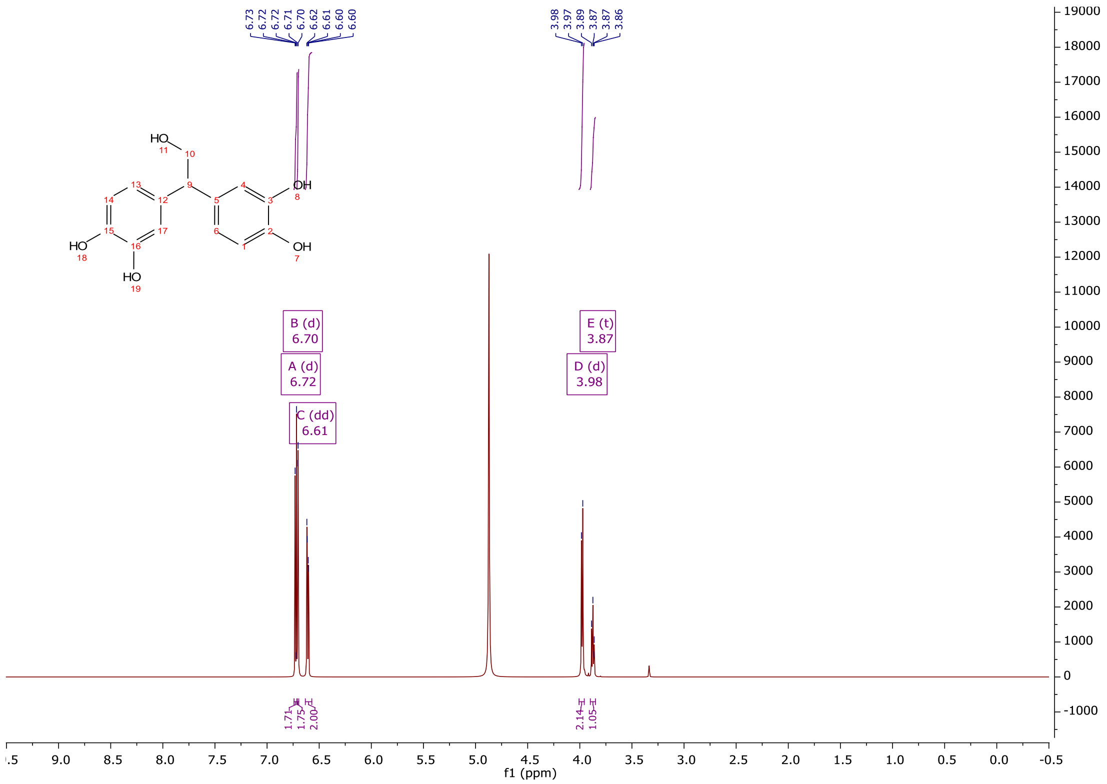


<u>Figure S21</u>: $^{1}$H NMR of compound **39b** in MeOD

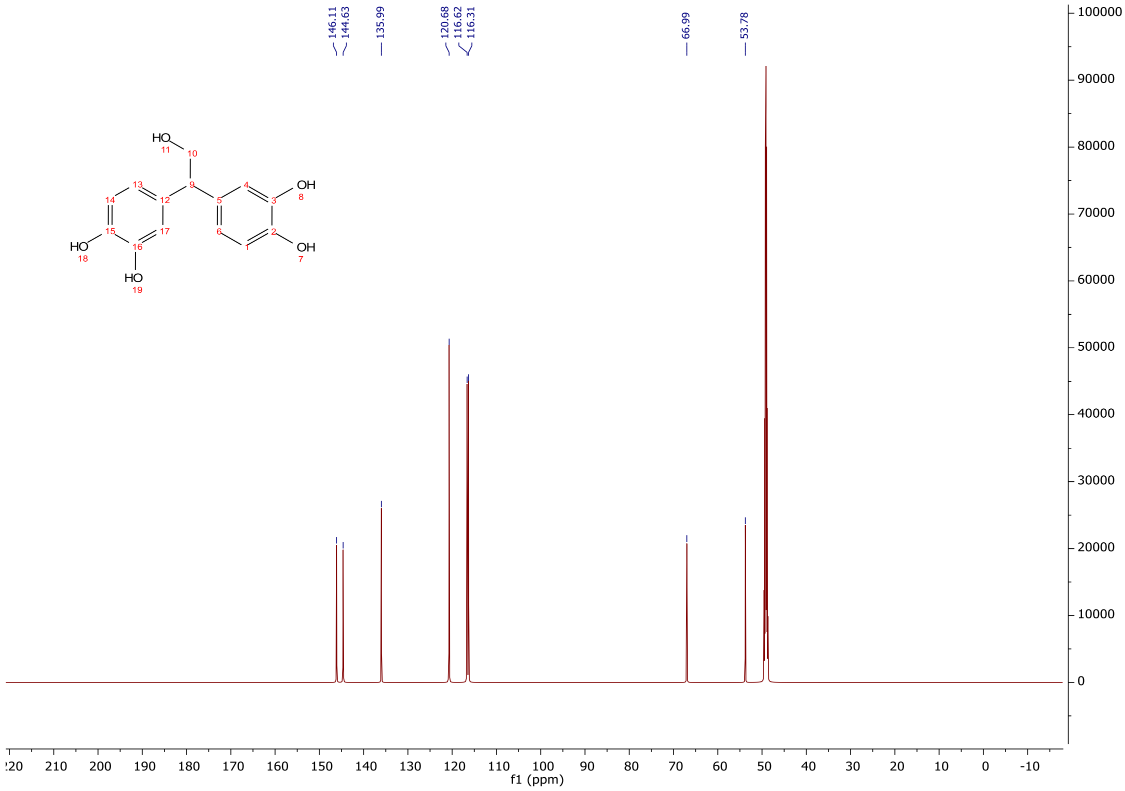


<u>Figure S22:</u> C$^{13}$ NMR of compound **39b** in MeOD
